# Emotional and behavioral problems change the development of cerebellar gray matter volume, thickness, and surface area from childhood to adolescence: A longitudinal cohort study

**DOI:** 10.1111/cns.14286

**Published:** 2023-06-08

**Authors:** Yanpei Wang, Leilei Ma, Rui Chen, Ningyu Liu, Haibo Zhang, Yuanyuan Li, Jiali Wang, Mingming Hu, Gai Zhao, Weiwei Men, Shuping Tan, Jia‐Hong Gao, Shaozheng Qin, Yong He, Qi Dong, Sha Tao

**Affiliations:** ^1^ State Key Laboratory of Cognitive Neuroscience and Learning Beijing Normal University Beijing China; ^2^ IDG/McGovern Institute for Brain Research Beijing Normal University Beijing China; ^3^ Center for MRI Research, Academy for Advanced Interdisciplinary Studies Peking University Beijing China; ^4^ Psychiatry Research Center, Beijing HuiLongGuan Hospital Peking University Beijing China

**Keywords:** cerebellum, cortical thickness, developmental trajectory, emotional and behavioral problems, gray matter volume, surface area

## Abstract

**Aims:**

Increasing evidence indicates that major neurodevelopmental disorders have potential links to abnormal cerebellar development. However, the developmental trajectories of cerebellar subregions from childhood to adolescence are lacking, and it is not clear how emotional and behavioral problems affect them. We aim to map the developmental trajectories of gray matter volume (GMV), cortical thickness (CT), and surface area (SA) in cerebellar subregions from childhood to adolescence and examine how emotional and behavioral problems change the cerebellar development trajectory in a longitudinal cohort study.

**Method:**

This population‐based longitudinal cohort study used data on a representative sample of 695 children. Emotional and behavioral problems were assessed at baseline and at three annual follow‐ups with the Strengths and Difficulties Questionnaire (SDQ).

**Results:**

Using an innovative automated image segmentation technique, we quantified the GMV, CT, and SA of the whole cerebellum and 24 subdivisions (lobules I‐VI, VIIB, VIIIA&B, and IX‐X plus crus I‐II) with 1319 MRI scans from a large longitudinal sample of 695 subjects aged 6–15 years and mapped their developmental trajectories. We also examined sex differences and found that boys showed more linear growth, while girls showed more nonlinear growth. Boys and girls showed nonlinear growth in the cerebellar subregions; however, girls reached the peak earlier than boys. Further analysis found that emotional and behavioral problems modulated cerebellar development. Specifically, emotional symptoms impede the expansion of the SA of the cerebellar cortex, and no gender differences; conduct problems lead to inadequate cerebellar GMV development only in girls, but not boys; hyperactivity/inattention delays the development of cerebellar GMV and SA, with left cerebellar GMV, right VIIIA GMV and SA in boys and left V GMV and SA in girls; peer problems disrupt CT growth and SA expansion, resulting in delayed GMV development, with bilateral IV, right X CT in boys and right Crus I GMV, left V SA in girls; and prosocial behavior problems impede the expansion of the SA and lead to excessive CT growth, with bilateral IV, V, right VI CT, left cerebellum SA in boys and right Crus I GMV in girls.

**Conclusions:**

This study maps the developmental trajectories of GMV, CT, and SA in cerebellar subregions from childhood to adolescence. In addition, we provide the first evidence for how emotional and behavioral problems affect the dynamic development of GMV, CT, and SA in the cerebellum, which provides an important basis and guidance for the prevention and intervention of cognitive and emotional behavioral problems in the future.

## Graphical abstract

This study maps the developmental trajectories of gray matter volume (GMV), cortical thickness (CT), and surface area (SA) in cerebellar subregions from childhood to adolescence and provides the first evidence for how emotional and behavioral problems affect the dynamic development of GMV, CT, and SA, which provides an important basis and guidance for the prevention and intervention of cognitive and emotional and behavioral problems in the future.

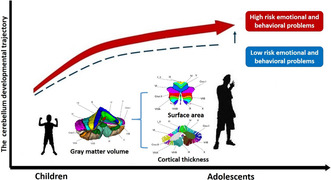

## 1 INTRODUCTION

The cerebellum lies beneath the cerebrum and is an important bilateral neuroanatomical structure connected to the brainstem and cerebrum by two bundles of white matter fibers known as cerebellar peduncles. The cerebellum's unique protracted developmental trajectories, gender dimorphism, preferential vulnerability to environmental influences, and frequent impact on childhood developmental disorders such as autism and attention deficit hyperactivity disorder (ADHD) make it a major target for pediatric neuroimaging studies.^1^ Knowledge of the developing cerebellum from childhood to adolescence is invaluable in understanding the neural basis of the development of various cognitive, emotional, and behavioral problems and neurological disorders.^2^

To date, only a few studies^3^, ^4^, ^5^ have examined age‐related differences or longitudinal changes in cerebellar volume in children and adolescents. Wierenga et al.^4^ explored the typical development of cerebellar whole volume from age 7 to 24 with a longitudinal sample. While some other studies have examined the development of the cerebellar subregions, understanding of the developmental trajectories at the level of subregions of the cerebellum in children and adolescents is still limited. Tiemeier et al.^3^ examined the developmental trajectories of five subregions (inferior posterior lobe, superior posterior lobe, anterior lobe, corpus medullare, and vermis) in only 50 children. While Romero et al.^5^ examined the development of 12 cerebellar subregions with a large cross‐sectional dataset (*N* = 2515 images), the developmental trajectories among children and adolescents were not depicted.

All of these cerebellar studies on cerebellar development mainly focused on gray matter volume (GMV). However, the brain GMV consists of surface area (SA) and cortical thickness (CT), each exhibiting differentiated developmental patterns during adolescence.^6^ Therefore, it is important to investigate the developmental curve of the cerebellum in three dimensions, GMV, CT, and SA. A few recent studies have begun to extend to the CT of the cerebellum cortex.^7^, ^8^ To our knowledge, the different developmental trajectories of cerebellar CT and SA during childhood and adolescence have not been revealed. CT is the distance from the boundary of gray/white matter to the boundary of the outer surface of the brain, partly indicative of the number of cells within cortical columns.^9^ The SA describes the 2D extent of the cortex compared with a standard brain, and it seems more closely related to the number in the cortical column.^9^ CT and SA are genetically unrelated^10^ and may show distinct and heterogeneous development patterns during childhood.^6^, ^11^ Thus, this study aims to reveal the developmental trajectory of cerebellar GMV, CT, and SA with a large longitudinal study.

The human cerebellum (11th postnatal month) has a longer developmental timeline than the neocortex (prenatal period),^12^, ^13^ which expands the window of vulnerability to neurological diseases.^14^ The cerebellum has long been thought to play a role only in motor coordination. However, research studies over the past two decades have shown that the cerebellum also plays a key role in many motor, cognitive, and emotional processes. Recently, evidence has suggested that major neurodevelopmental disorders such as intellectual disability (ID), autism spectrum disorder (ASD), attention deficit hyperactivity disorder (ADHD), schizophrenia, bipolar disorder, major depression, and anxiety disorders have potential links to abnormal cerebellar development.^14^, ^15^ Brain imaging studies have revealed neurodevelopmental disorders of the cerebellum, including ASD, ADHD, ID, communication disorders, and childhood motor disorders.^14^, ^16^, ^17^ However, many cerebellar‐related disorders are continuous rather than dichotomous,^18^ and based on the comparisons of group differences, dividing continuous variables into dichotomous variables does not provide a good insight of the causes of disease formation. For example, both cerebellar studies based on autism and attentional disorders have found developmental delays in the cerebellum, which means that the results of comparisons between groups may not be same at different developmental stages.^14^, ^17^, ^19^ It is possible to observe the cumulative effects of cognitive, emotional, and behavioral problems on cerebellar and neuropsychological problems through long‐term longitudinal cohort studies. However, as the most critical period for cerebellar development in childhood and adolescence, the developmental trajectory based on GMV, CT, and SA of cerebellar fine subareas and the influence of emotional and behavioral problems on them are still lacking.

Thus, our first aim was to delineate the development of cerebellar subregions from childhood to adolescence using the GMV, CT and SA profile to describe the trajectories. Second, we sought to explore how emotional and behavioral problems affect cerebellar development trajectory and eventually lead to cerebellar‐related neurodevelopmental disorders to provide evidence support for understanding the biological mechanisms underlying the behavior. We used a novel automated segmentation pipeline applied to 1319 MRI scans from 695 children and adolescents aged 6–15 years, drew the development trajectory of cerebellar GMV, CT, and SA, and investigated the effects of emotional and behavioral problems on cerebellar developmental trajectory from five aspects: emotional symptoms, conduct problems, hyperactivity/inattention, peer problems, and prosocial behaviors.

## 2 MATERIALS AND METHODS

### 2.1 Participants

We used a longitudinal structural MRI dataset comprising 770 normally developing children aged 6–15 years (F/M = 334/436) from the Children School Functions and Brain Development project (CBD, Beijing Cohort). Participants underwent 2–4 repeated MRI scans at approximately 1‐year intervals, leading to 1623 structural MRI scans in total. All participants were recruited from primary schools in Beijing with normal cognitive ability, assessed by a well‐validated Chinese standardized cognitive ability test.^20^ Exclusion criteria included notable physical illness or head trauma and a history of neurological/psychiatric disorders. None of the children had taken any drugs or consumed caffeine on the day of the behavioral tests and MRI scans. In total, 97 structural MRI scans were excluded due to artifacts after data quality control, and 207 structural MRI scans were removed for not completing assessments of emotional and behavioral problems. Finally, structural MRI data of 695 children (6–15 years old at baseline, F/M = 316/379, 1319 scans in total) remained, wherein four scans were available for 42 children (F/M = 21/21), three scans were available for 149 children (F/M = 68/81), two scans were available for 200 children (F/M = 87/113), and one scan was available for 304 children (F/M = 140/164). All children's parents/guardians signed an informed consent form approved by the Ethics Committee of Beijing Normal University.

### 2.2 Assessments of emotional and behavioral problems

The parent‐reported version of the Strengths and Difficulties Questionnaire (SDQ) was applied to assess emotional and behavioral problems in children and adolescents. The Chinese version was retrieved from the SDQ website (https://www.sdqinfo.org/py/sdqinfo/b0.py), which includes multiple language versions. The SDQ included 25 items that comprised five subscales (emotional symptoms, conduct problems, hyperactivity or peer problems, and a prosocial behavior scale).^21^

The emotional and behavioral problems were often caused by a combination of environment and genetics.^22^, ^23^

### 2.3 Image acquisition

All structural T1 images were obtained at Peking University, Huilongguan Hospital and Beijing Normal University, Beijing. The three sites used three identical Siemens Prisma 3T MRI scanners with 64‐channel head coils and the same parameters. T1‐weighted anatomical scans with the following parameters were obtained at each time point: TR = 2530 ms, TE = 2.98 ms, inversion time = 1100 ms, FA = 7°, FOV = 256 × 224 mm^2^, matrix size = 256 × 224, slice thickness = 1 mm, and scan time = 5 min and 58 s.

### 2.4 MRI quality assurance

All MRI scan quality control procedures are described below. (i) Individual images were carefully visually examined by an experienced radiologist to rule out incidental abnormalities, such as neuroepithelial cysts, arachnoid cysts, and other intracranial space‐occupying lesions. (ii) Five experienced raters each performed careful visual inspections with a scan rating procedure, with a protocol similar to that of the Human Connectome Project.^24^ (iii) Images that received high‐quality ratings from all raters were retained.

### 2.5 Data preprocessing

**FIGURE 1:**
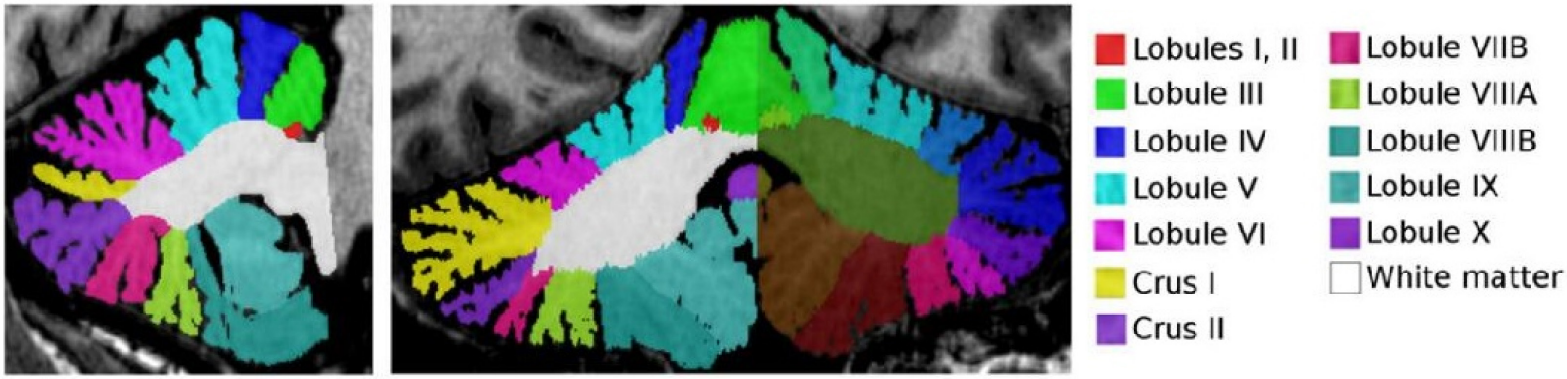
The segmentation of cerebellum.

We computed the cerebellar GMV, CT, and SA using the automated software pipeline CERES (https://www.volbrain.upv.es).^5^ Briefly, the T1w image of each subject was denoised, and the intensity inhomogeneities were corrected and registered to the MNI152 space. The image was then cropped and normalized to the MNI152 cerebellum atlas. After preprocessing, CERES applied automated cerebellum patch‐based segmentation and produced the GMV and mean CT of cerebellar lobules I‐II, III, IV, V, VI, VIIB, VIIIA, VIIIB, IX, and X and Crus I and II as output, as shown in Figure 1.

### 2.6 Statistical analysis

Kolmogorov–Smirnov test was used for assessing the normality of data, and the data that do not exhibit a normal/Gaussian distribution is transformed by the Box–Cox transformation.^25^ Then, linear changes were made to keep the same range of data after the transformation as before. This is done in two steps: convert the *Z*‐score, then multiply it by the standard deviation of the original value plus add the mean of the original value.

We performed statistical analyses using R 4.1.1 (https://www.r‐project.org/). To investigate the developmental trajectories of the GMV, CT, and SA of the total cerebellum and each of the 12 cerebellar subregions and the effects of sex, we used mixed models with the nlme package.^26^

Mixed modeling approaches are very suitable for accelerating longitudinal designs and can deal with missing data; hence, they are widely used.^27^ Each dependent measure of the *i*th family, *j*th individual, and *k*th time‐point was modeled as described by Raznahan et al.^28^ We fitted several models, including linear age terms, quadratic age terms, cubic age terms, and gender terms. The following equation shows the full model:

$$\begin{array}{cc} {\text{Measurement}}_{\mathrm{ijk}}= & \text{Intercept}+d_{\mathrm{ij}}+\beta_{0}\;\left(\text{site}\right)+\beta_{1}\;\left(\text{gender}\right)+\beta_{2}\;\left(\mathrm{age}\right)\\ & +\beta_{3}\;\left({\mathrm{age}}^{2}\right)+\beta_{4}\;\left({\mathrm{age}}^{3}\right)+\beta_{3}\;\left(\text{gender}\times \mathrm{age}\right)\\ & +\beta_{6}\;\left(\text{gender}\times {\mathrm{age}}^{2}\right)+\beta_{7}\;\left(\text{gender}\times {\mathrm{age}}^{3}\right)+e_{\mathrm{ijk}} \end{array}$$

The *e*_*ijk*_ term represents the residual error of the normal distribution. Each *β* represents a parameter estimate; for example, the quadratic age effect parameter is represented by *β*_3_. Furthermore, the interaction effects of gender and age are modeled. The full model was compared with models including only linear or quadratic age terms. Intercept, gender, and age were fixed effects, while within‐person dependence nested within subject (*d*_*ij*_) was modeled as a random effect.

The best model fit was selected based on a three‐step process. First, cubic, quadratic, and linear age effects were fitted on the GMV, CT, and SA of each ROI (region of interest). If the cubic age effect was not significant at *p* < 0.05, we used the quadratic developmental model and then the linear developmental model. Second, we probed whether the developmental trajectories differed by gender. Hence, a full model including the main effects of age and gender, as well as interaction effects, was compared using the Bayesian Information Criterion (BIC) to a simpler model including only the main effects of age and gender. Whether the full model allowing for differences in growth trajectories between genders explained more variance than the simpler model was tested. Third, if no advantage is found for the full model, the simpler model incorporating the main effects of age and gender was compared with the best‐fit developmental model selected in step one that included only age terms. The best‐fit model was again selected using BIC to obtain the model that accounted for the greatest variance in the number of parameters included in the model and to reduce the threat of data overfitting.

Finally, we investigated how children's emotional and behavioral problems affect the development of cerebellar subregions. Then we added this continuous emotional and behavioral problems score (centered) to the best fitting mixed model and inspected the significance of its main and age interaction terms. To visualize and better show the role of emotional and behavioral problems, the sample was split into two subgroups based on scores for emotional and behavioral problems^29^: relatively low (mean = 0.48, SD = 0.50, range = 0–1) and relatively high (mean = 2.90, SD = 1.14, range = 2–8) emotional symptoms, low (mean = 0.73, SD = 0.45, range = 0–1) and relatively high (mean = 2.63, SD = 0.91, range = 2–7) conduct problems, low (mean = 2.82, SD = 1.57, range = 0–5) and relatively high (mean = 7.15, SD = 1.16, range = 6–10) hyperactivity/inattention, low (mean = 1.45, SD = 1.03, range = 0–3) and relatively high (mean = 4.46, SD = 0.75, range = 4–8) peer problems and low (mean = 8.64, SD = 1.14, range = 7–10) and relatively high (mean = 5.08, SD = 1.03, range = 0–6) prosocial behaviors.

To achieve an optimal balance between Type‐I and Type‐II error, we took into account the correlation between the dependent variables (the 12 cerebellar subregions) by using a Bonferroni procedure adjusted for correlated variables (http://www.quantitativeskills.com/sisa/calculations/bonfer.htm).^30^, ^31^ Bonferroni correction treats the variables as independent (proper Bonferroni: *α*/number of tests) and would lead to an overly strict correction because the dependent variables are not obtained in independent subgroups. GMV showed a mean correlation coefficient of *r* = 0.3641, leading to an equivalent corrected *α* of 0.0066 (number of tests = 24). CT showed a mean correlation coefficient of *r* = 0.3499, resulting in an equivalent corrected *α* of 0.0065. SA showed a mean correlation coefficient of *r* = 0.3103, leading to an equivalent corrected *α* of 0.0056.

## 3 RESULTS

### 3.1 Developmental trajectories

**FIGURE 2:**
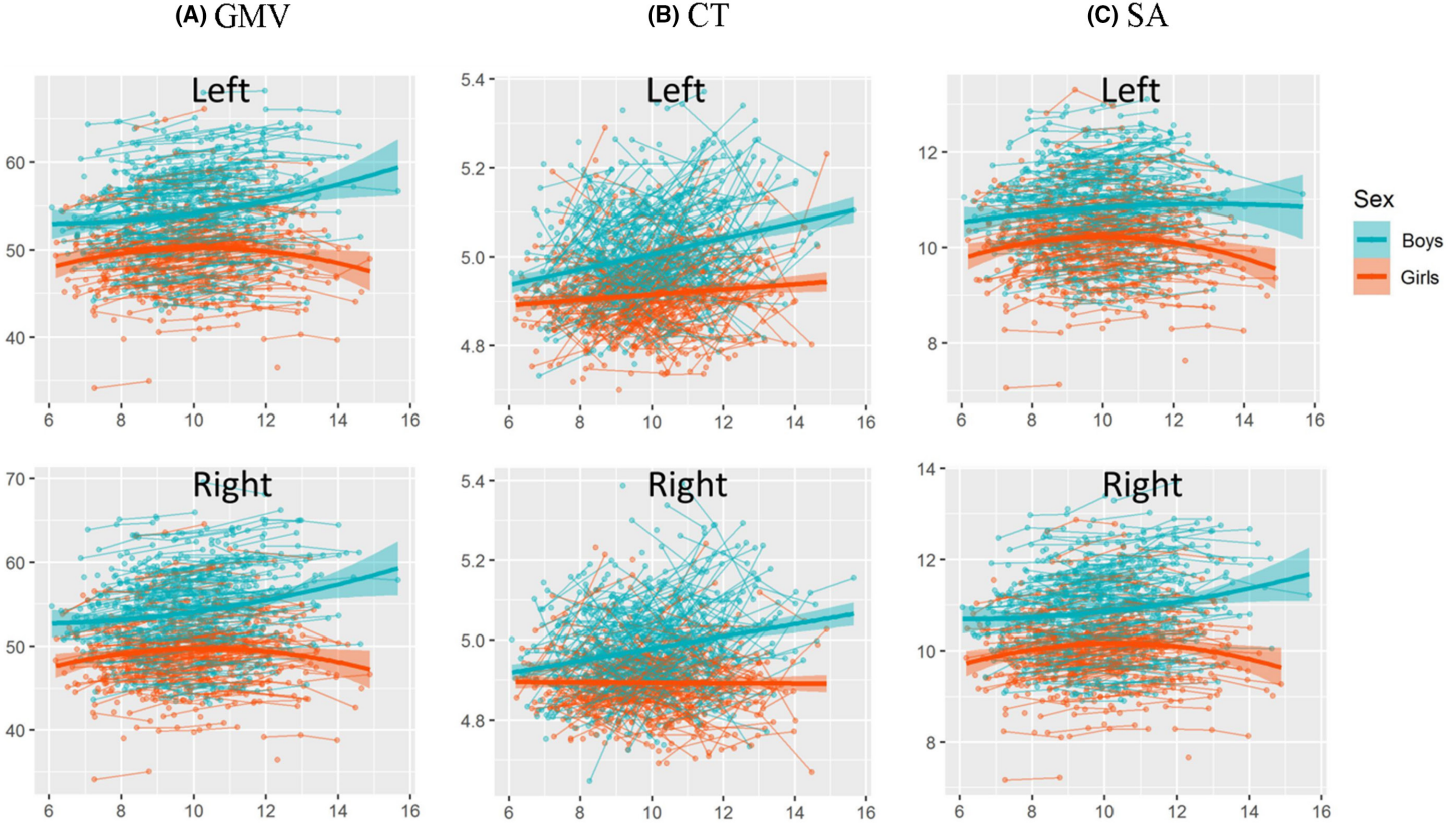
Development of the whole cerebellum. (A) GMV in cm^3^, (B) CT in cm, and (C) SA in cm^2^ (*y*‐axis) by age (*x*‐axis) and the optimal fitting model (quadratic, linear and quadratic) are shown. The shaded areas represent the 95% confidence intervals. Individual boys (blue) and girls (orange) are represented by individual lines, and participants measured once are represented by dots.

**FIGURE 3:**
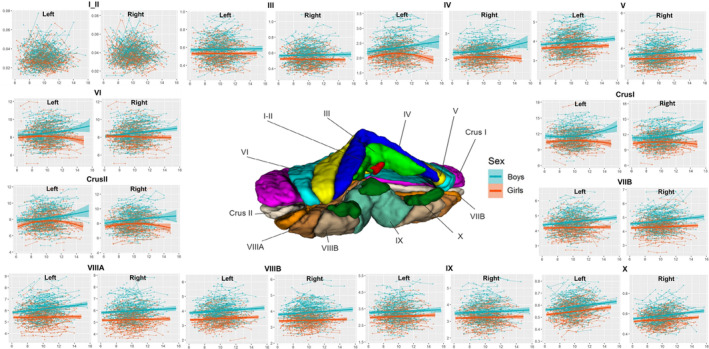
Development of GMV in cerebellar subregions. GMV (*y*‐axis) by age (*x*‐axis) and the optimal fitting model are shown. The shaded areas represent the 95% confidence intervals. Individual boys (blue) and girls (orange) are represented by individual lines, and participants measured once are represented by dots.

**FIGURE 4:**
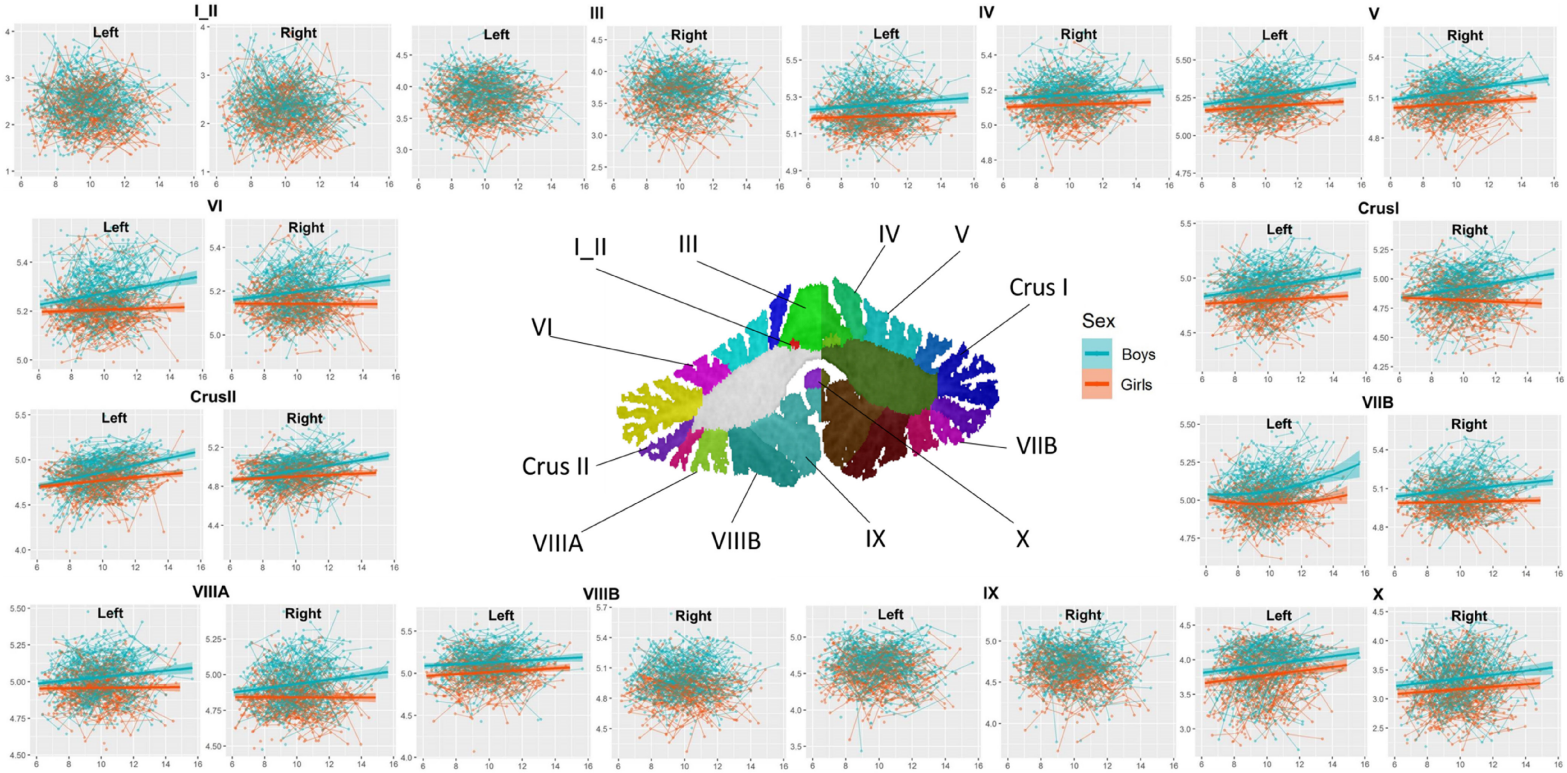
Development of CT in cerebellar subregions. CT (*y*‐axis) by age (*x*‐axis) and the optimal fitting models are shown. The shaded areas represent the 95% confidence intervals. Individual boys (blue) and girls (orange) are represented by individual lines, and participants measured once are represented by dots.

**FIGURE 5:**
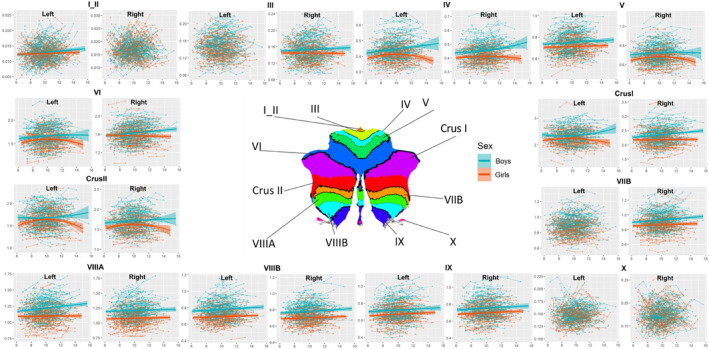
Development of SA in cerebellar subregions. SA (*y*‐axis) by age (*x*‐axis) and the optimal fitting models are shown. The shaded areas represent the 95% confidence intervals. Individual boys (blue) and girls (orange) are represented by individual lines, and participants measured once are represented by dots.

The results of the regression models for the GMV, CT, and SA of the whole cerebellum and subregions are shown in Figures 2, 3, 4, 5 and Tables 1, 2, 3. The regression model parameters of both boys and girls are shown in Tables 4, 5, 6. Whole bilateral cerebellar GMV (left: peak = 14.36, 95% CI = 10.74–17.98; right: peak = 15.07, 95% CI = 11.26–19.89) and SA (left: peak = 11.86, 95% CI = 11.12–12.60; right: peak = 13.65, 95% CI = 12.31–14.99) followed quadratic trajectories, while CT increased in a linear fashion. Specifically, the GMV, CT, and SA were larger for boys than for girls over the entire age range. Boys showed increasing linear trajectories for GMV, CT, and SA. In contrast, GMV and SA in girls followed quadratic and cubic curvilinear trajectories while showing linear growth in CT (Tables 4, 5, 6 and Figure 2).

**TABLE 1:** Best fit regression model for each GMV and parameters for developmental trajectories.

GMV	HS	Best fitting model	Intercept	Site *β*_0_	Gender coefficient *β*_1_	Age coefficient *β*_2_	Age^2^ coefficient *β*_3_	Age × gender coefficient *β*_5_	Age^2^ × gender coefficient *β*_6_
Cerebellum	L	Quadratic	4.11 × 10	n.s.	7.48	1.60	−6.50 × 10^−2^	n.s.	4.29 × 10^−2^
	R	Quadratic	4.04 × 10	n.s.	8.22	1.64	−6.65 × 10^−2^	−8.42 × 10^−1^	4.74 × 10^−2^
Lobule I‐II	L	None	‐	‐	‐	‐	‐	‐	‐
	R	None	‐	‐	‐	‐	‐	‐	‐
Lobule III	L	Linear	4.97 × 10^−1^	n.s.	5.00 × 10^−2^	4.48 × 10^−3^	‐	‐	‐
	R	Linear	4.80 × 10^−1^	n.s.	5.50 × 10^−2^	4.23 × 10^−3^	‐	‐	‐
Lobule IV	L	Quadratic	1.90	−6.18 × 10^−2^	2.12 × 10^−1^	5.00 × 10^−2^	−1.89 × 10^−3^	‐	‐
	R	Quadratic	1.78	−5.53 × 10^−2^	2.39 × 10^−1^	6.43 × 10^−2^	−2.41 × 10^−3^	‐	‐
Lobule V	L	Linear	3.38	n.s.	2.44 × 10^−1^	3.75 × 10^−2^	‐	‐	‐
	R	Linear	3.19	n.s.	2.99 × 10^−1^	2.85 × 10^−2^	‐	‐	‐
Lobule VI	L	Quadratic	6.45	n.s.	1.24	3.42 × 10^−1^	−1.52 × 10^−2^	n.s.	1.10 × 10^−2^
	R	Linear	7.51	n.s.	n.s.	5.42 × 10^−2^	‐	3.10 × 10^−2^	‐
Lobule Crus I	L	Quadratic	9.08	n.s.	9.14 × 10^−1^	2.74 × 10^−1^	−1.08 × 10^−2^	‐	‐
	R	Quadratic	7.80	n.s.	3.11	5.22 × 10^−1^	−2.31 × 10^−2^	−4.57 × 10^−1^	2.42 × 10^−2^
Lobule Crus II	L	Quadratic	6.27	n.s.	4.68 × 10^−1^	2.66 × 10^−1^	−1.10 × 10^−2^	‐	‐
	R	Quadratic	6.36	n.s.	6.39 × 10^−1^	2.92 × 10^−1^	−1.27 × 10^−2^	‐	‐
Lobule VIIB	L	Linear	4.04	n.s.	3.63 × 10^−1^	2.32 × 10^−2^	‐	‐	‐
	R	Linear	4.09	n.s.	4.27 × 10^−1^	2.80 × 10^−2^	‐	‐	‐
Lobule VIIIA	L	Linear	4.99	n.s.	6.99 × 10^−1^	5.11 × 10^−2^	‐	‐	‐
	R	Linear	4.84	n.s.	7.11 × 10^−1^	4.73 × 10^−2^	‐	‐	‐
Lobule VIIIB	L	Linear	3.10	n.s.	5.37 × 10^−1^	4.00 × 10^−2^	‐	‐	‐
	R	Linear	3.13	n.s.	4.86 × 10^−1^	3.05 × 10^−2^	‐	‐	‐
Lobule IX	L	Linear	2.96	n.s.	2.92 × 10^−1^	1.61 × 10^−2^	‐	‐	‐
	R	Linear	3.15	n.s.	3.27 × 10^−1^	1.63 × 10^−2^	‐	‐	‐
Lobule X	L	Linear	4.75 × 10^−1^	n.s.	4.13 × 10^−2^	8.04 × 10^−3^	‐	‐	‐
	R	Linear	4.76 × 10^−1^	n.s.	3.98 × 10^−2^	6.91 × 10^−3^	‐	‐	‐

*Note*: The level of significance is 0.0066 after multiple comparisons correction.

Abbreviations: HS, Hemisphere; L, left; n.s., non‐significant; R, right; ‐, not applicable.

**TABLE 2:** Best fit regression model for each CT and parameters for developmental trajectories.

CT	HS	Best fitting model	Intercept	Site *β*_0_	Gender coefficient *β*_1_	Age coefficient *β*_2_	Age^2^ coefficient *β*_3_	Age × gender coefficient *β*_5_	Age^2^ × gender coefficient *β*_6_
Cerebellum	L	Linear	4.81	n.s.	n.s.	9.60 × 10^−3^	‐	9.18 × 10^−3^	‐
	R	Linear	4.86	n.s.	n.s.	n.s.	‐	1.15 × 10^−2^	‐
Lobule I‐II	L	None	‐	‐	‐	‐	‐	‐	‐
	R	None	‐	‐	‐	‐	‐	‐	‐
Lobule III	L	None	‐	‐	‐	‐	‐	‐	‐
	R	None	‐	‐	‐	‐	‐	‐	‐
Lobule IV	L	Linear	5.13	n.s.	5.93 × 10^−2^	6.78 × 10^−3^	‐	‐	‐
	R	Linear	5.06	n.s.	5.78 × 10^−2^	6.22 × 10^−3^	‐	‐	‐
Lobule V	L	Linear	5.09	n.s.	6.65 × 10^−2^	1.11 × 10^−2^	‐	‐	‐
	R	Linear	4.92	n.s.	9.09 × 10^−2^	1.32 × 10^−2^	‐	‐	‐
Lobule VI	L	Linear	5.14	n.s.	6.50 × 10^−2^	6.52 × 10^−3^	‐	‐	‐
	R	Linear	5.15	n.s.	n.s.	n.s.	‐	8.66 × 10^−3^	‐
Lobule Crus I	L	Linear	4.58	n.s.	1.21 × 10^−1^	2.17 × 10^−2^	‐	‐	‐
	R	Linear	4.78	n.s.	n.s.	n.s.	‐	1.38 × 10^−2^	‐
Lobule Crus II	L	Linear	4.58	n.s.	n.s.	1.98 × 10^−2^	‐	1.92 × 10^−2^	‐
	R	Linear	4.82	n.s.	n.s.	n.s.	‐	2.00 × 10^−2^	‐
Lobule VIIB	L	Quadratic	5.12	n.s.	8.36 × 10^−2^	n.s.	2.45 × 10^−3^	‐	‐
	R	Linear	4.90	n.s.	9.19 × 10^−2^	9.04 × 10^−3^	‐	‐	‐
Lobule VIIIA	L	Linear	4.86	n.s.	6.95 × 10^−2^	9.15 × 10^−3^	‐	‐	‐
	R	Linear	4.76	n.s.	8.66 × 10^−2^	8.75 × 10^−3^	‐	‐	‐
Lobule VIIIB	L	Linear	4.82	2.76 × 10^−2^	1.15 × 10^−1^	1.58 × 10^−2^	‐	‐	‐
	R	None	‐	‐	‐	‐	‐	‐	‐
Lobule IX	L	None	‐	‐	‐	‐	‐	‐	‐
	R	None	‐	‐	‐	‐	‐	‐	‐
Lobule X	L	Linear	3.35	n.s.	1.47 × 10^−1^	3.75 × 10^−2^	‐	‐	‐
	R	Linear	2.75	n.s.	1.79 × 10^−1^	3.55 × 10^−2^	‐	‐	‐

*Note*: The level of significance is 0.0065 after multiple comparisons correction.

Abbreviations: HS, Hemisphere; L, left; n.s., non‐significant; R, right; ‐, not applicable.

**TABLE 3:** Best fit regression model for each SA and parameters for developmental trajectories.

SA	HS	Best fitting model	Intercept	Site *β*_0_	Gender coefficient *β*_1_	Age coefficient *β*_2_	Age^2^ coefficient *β*_3_	Age × gender coefficient *β*_5_	Age^2^ × gender coefficient *β*_6_
Cerebellum	L	Quadratic	8.65	n.s.	1.46	2.98 × 10^−1^	−1.32 × 10^−2^	n.s.	8.33 × 10^−3^
	R	Quadratic	8.59	n.s.	1.64	2.79 × 10^−1^	−1.15 × 10^−2^	−1.81 × 10^−1^	9.14 × 10^−3^
Lobule I‐II	L	Linear	1.13 × 10^−2^	n.s.	n.s.	1.46 × 10^−4^	‐	‐	‐
	R	None	‐	‐	‐	‐	‐	‐	‐
Lobule III	L	Linear	1.35 × 10^−1^	n.s.	8.32 × 10^−3^	8.85 × 10^−4^	‐	‐	‐
	R	Linear	1.38 × 10^−1^	n.s.	9.27 × 10^−3^	8.21 × 10^−4^	‐	‐	‐
Lobule IV	L	Quadratic	3.69 × 10^−1^	−1.15 × 10^−2^	3.57 × 10^−2^	9.66 × 10^−3^	−4.01 × 10^−4^	‐	‐
	R	Quadratic	3.53 × 10^−1^	−1.05 × 10^−2^	4.15 × 10^−2^	1.20 × 10^−2^	−4.77 × 10^−4^	‐	‐
Lobule V	L	Linear	6.67 × 10^−1^	n.s.	3.73 × 10^−2^	5.46 × 10^−3^	‐	‐	‐
	R	Quadratic	5.90 × 10^−1^	n.s.	4.59 × 10^−2^	1.59 × 10^−2^	−6.01 × 10^−4^	‐	‐
Lobule VI	L	Quadratic	1.35	n.s.	6.99 × 10^−2^	4.23 × 10^−2^	−1.69 × 10^−3^	‐	‐
	R	Linear	1.44	n.s.	5.52 × 10^−2^	1.26 × 10^−2^	‐	‐	‐
Lobule Crus I	L	Quadratic	2.06	n.s.	1.30 × 10^−1^	3.70 × 10^−2^	−1.87 × 10^−3^	‐	‐
	R	Linear	2.12	n.s.	1.60 × 10^−1^	7.73 × 10^−3^	‐	‐	‐
Lobule Crus II	L	Quadratic	1.27	n.s.	4.35 × 10^−1^	7.34 × 10^−2^	−3.64 × 10^−3^	−7.26 × 10^−2^	3.46 × 10^−3^
	R	Quadratic	1.41	n.s.	1.08 × 10^−1^	4.38 × 10^−2^	−2.18 × 10^−3^	‐	‐
Lobule VIIB	L	None	‐	‐	‐	‐	‐	‐	‐
	R	Linear	8.41 × 10^−1^	n.s.	6.81 × 10^−2^	3.52 × 10^−3^	‐	‐	‐
Lobule VIIIA	L	Linear	1.03	n.s.	1.24 × 10^−1^	7.65 × 10^−3^	‐	‐	‐
	R	Linear	1.02	n.s.	1.26 × 10^−1^	7.49 × 10^−3^	‐	‐	‐
Lobule VIIIB	L	Linear	6.51 × 10^−1^	n.s.	8.94 × 10^−2^	5.09 × 10^−3^	‐	‐	‐
	R	Linear	6.50 × 10^−1^	n.s.	8.11 × 10^−2^	5.49 × 10^−3^	‐	‐	‐
Lobule IX	L	Linear	6.56 × 10^−1^	−1.67 × 10^−2^	4.90 × 10^−2^	3.62 × 10^−3^	‐	‐	‐
	R	Linear	6.76 × 10^−1^	n.s.	5.47 × 10^−2^	4.17 × 10^−3^	‐	‐	‐
Lobule X	L	None	‐	‐	‐	‐	‐	‐	‐
	R	None	‐	‐	‐	‐	‐	‐	‐

*Note*: The level of significance is 0.0056 after multiple comparisons correction.

Abbreviations: HS, Hemisphere; L, left; n.s., non‐significant; R, right; ‐, not applicable.

**TABLE 4:** Best fit regression model for each GMV and parameters for developmental trajectories in boys and girls.

GMV	HS	Boys						Girls					
		Best fitting model	Intercept	Site *β*_0_	Age coefficient *β*_1_	Age^2^ coefficient *β*_2_	Age^3^ coefficient *β*_3_	Best fitting model	Intercept	Site *β*_0_	Age coefficient *β*_1_	Age^2^ coefficient *β*_2_	Age^3^ coefficient *β*_3_
Cerebellum	L	Linear	5.14 × 10	n.s.	3.87 × 10^−1^	‐	‐	Cubic	5.43 × 10	n.s.	n.s.	3.55 × 10^−1^	−1.37 × 10^−2^
	R	Linear	5.10 × 10	n.s.	4.07 × 10^−1^	‐	‐	Cubic	5.43 × 10	n.s.	n.s.	3.77 × 10^−1^	−1.44 × 10^−2^
Lobule I‐II	L	None	‐	‐	‐	‐	‐	None	‐	‐	‐	‐	‐
	R	None	‐	‐	‐	‐	‐	None	‐	‐	‐	‐	‐
Lobule III	L	Linear	5.49 × 10^−1^	n.s.	3.66 × 10^−3^	‐	‐	Linear	4.94 × 10^−1^	n.s.	5.55 × 10^−3^	‐	‐
	R	Linear	5.33 × 10^−1^	n.s.	3.82 × 10^−3^	‐	‐	Linear	4.82 × 10^−1^	n.s.	4.67 × 10^−3^	‐	‐
Lobule IV	L	Linear	2.31	−8.23 × 10^−2^	1.37 × 10^−2^	‐	‐	Quadratic	1.71	n.s.	8.63 × 10^−2^	−3.85 × 10^−3^	‐
	R	Linear	2.26	n.s.	1.68 × 10^−2^	‐	‐	Quadratic	1.72	n.s.	7.45 × 10^−2^	−3.04 × 10^−3^	‐
Lobule V	L	Linear	3.60	n.s.	3.96 × 10^−2^	‐	‐	Linear	3.41	n.s.	3.48 × 10^−2^	‐	‐
	R	Linear	3.55	n.s.	2.56 × 10^−2^	‐	‐	Linear	3.11	n.s.	3.20 × 10^−2^	‐	‐
Lobule VI	L	Linear	8.26	n.s.	6.61 × 10^−2^	‐	‐	Cubic	8.85	n.s.	n.s.	n.s.	−2.55 × 10^−3^
	R	Cubic	4.82	n.s.	9.73 × 10^−1^	−9.09 × 10^−2^	3.03 × 10^−3^	Cubic	8.73	n.s.	n.s.	n.s.	−2.62 × 10^−3^
Lobule Crus I	L	Linear	1.10 × 10	n.s.	6.88 × 10^−2^	‐	‐	Cubic	1.25 × 10	n.s.	n.s.	n.s.	−4.10 × 10^−3^
	R	Linear	1.10 × 10	n.s.	8.78 × 10^−2^	‐	‐	Quadratic	7.58	n.s.	5.22 × 10^−1^	−2.31 × 10^−2^	‐
Lobule Crus II	L	Linear	7.87	n.s.	4.99 × 10^−2^	‐	‐	Quadratic	5.38	n.s.	4.35 × 10^−1^	−1.99 × 10^−2^	‐
	R	Linear	8.31	n.s.	3.87 × 10^−2^	‐	‐	Quadratic	5.81	n.s.	3.95 × 10^−1^	−1.83 × 10^−2^	‐
Lobule VIIB	L	Linear	4.33	n.s.	2.83 × 10^−2^	‐	‐	None	‐	‐	‐	‐	‐
	R	Linear	4.43	n.s.	3.90 × 10^−2^	‐	‐	None	‐	‐	‐	‐	‐
Lobule VIIIA	L	Linear	5.69	n.s.	5.57 × 10^−2^	‐	‐	Linear	4.99	n.s.	4.48 × 10^−2^	‐	‐
	R	Linear	5.50	n.s.	5.04 × 10^−2^	‐	‐	Linear	4.90	n.s.	4.39 × 10^−2^	‐	‐
Lobule VIIIB	L	Linear	3.67	n.s.	4.08 × 10^−2^	‐	‐	Linear	3.06	n.s.	3.90 × 10^−2^	‐	‐
	R	Linear	3.56	n.s.	3.69 × 10^−2^	‐	‐	Linear	3.18	n.s.	2.52 × 10^−2^	‐	‐
Lobule IX	L	Linear	3.31	n.s.	1.69 × 10^−2^	‐	‐	Quadratic	2.46	n.s.	1.02 × 10^−1^	−4.30 × 10^−3^	‐
	R	Linear	3.53	n.s.	1.79 × 10^−2^	‐	‐	Linear	3.09	n.s.	1.42 × 10^−2^	‐	‐
Lobule X	L	Linear	5.17 × 10^−1^	n.s.	8.55 × 10^−3^	‐	‐	Linear	4.75 × 10^−1^	n.s.	7.36 × 10^−3^	‐	‐
	R	Linear	5.16 × 10^−1^	n.s.	7.28 × 10^−3^	‐	‐	Linear	4.75 × 10^−1^	n.s.	6.36 × 10^−3^	‐	‐

*Note*: The level of significance is 0.0066 after multiple comparisons correction.

Abbreviations: HS, Hemisphere; L, left; n.s., non‐significant; R, right; ‐, not applicable.

**TABLE 5:** Best fit regression model for each CT and parameters for developmental trajectories in boys and girls.

CT	HS	Boys						Girls					
		Best fitting model	Intercept	Site *β*_0_	Age coefficient *β*_1_	Age^2^ coefficient *β*_2_	Age^3^ coefficient *β*_3_	Best fitting model	Intercept	Site *β*_0_	Age coefficient *β*_1_	Age^2^ coefficient *β*_2_	Age^3^ coefficient *β*_3_
Cerebellum	L	Linear	4.82	n.s.	1.87 × 10^−2^	‐	‐	Linear	4.81	n.s.	9.63 × 10^−3^	‐	‐
	R	Linear	4.83	n.s.	1.50 × 10^−2^	‐	‐	None	‐	‐	‐	‐	‐
Lobule I‐II	L	None	‐	‐	‐	‐	‐	None	‐	‐	‐	‐	‐
	R	None	‐	‐	‐	‐	‐	None	‐	‐	‐	‐	‐
Lobule III	L	None	‐	‐	‐	‐	‐	None	‐	‐	‐	‐	‐
	R	None	‐	‐	‐	‐	‐	None	‐	‐	‐	‐	‐
Lobule IV	L	Linear	5.20	n.s.	6.78 × 10^−3^	‐	‐	Linear	5.13	n.s.	6.67 × 10^−3^	‐	‐
	R	Linear	5.10	n.s.	7.00 × 10^−3^	‐	‐	Linear	5.06	n.s.	7.07 × 10^−3^	‐	‐
Lobule V	L	Linear	5.15	n.s.	1.27 × 10^−2^	‐	‐	Linear	5.10	n.s.	8.96 × 10^−3^	‐	‐
	R	Linear	5.02	n.s.	1.28 × 10^−2^	‐	‐	Linear	4.90	n.s.	1.35 × 10^−2^	‐	‐
Lobule VI	L	Linear	5.18	n.s.	9.86 × 10^−3^	‐	‐	None	‐	‐	‐	‐	‐
	R	Linear	5.12	n.s.	8.31 × 10^−3^	‐	‐	None	‐	‐	‐	‐	‐
Lobule Crus I	L	Linear	4.64	n.s.	2.71 × 10^−2^	‐	‐	Linear	4.64	n.s.	1.50 × 10^−2^	‐	‐
	R	Linear	4.73	n.s.	1.97 × 10^−2^	‐	‐	None	‐	‐	‐	‐	‐
Lobule Crus II	L	Linear	4.49	n.s.	3.90 × 10^−2^	‐	‐	Linear	4.58	n.s.	1.99 × 10^−2^	‐	‐
	R	Linear	4.69	n.s.	2.93 × 10^−2^	‐	‐	Linear	4.82	n.s.	9.52 × 10^−3^	‐	‐
Lobule VIIB	L	Linear	4.90	n.s.	1.70 × 10^−2^	‐	‐	None	‐	‐	‐	‐	‐
	R	Linear	4.94	n.s.	1.46 × 10^−2^	‐	‐	None	‐	‐	‐	‐	‐
Lobule VIIIA	L	Linear	4.91	n.s.	1.18 × 10^−2^	‐	‐	None	‐	‐	‐	‐	‐
	R	Linear	4.80	n.s.	1.35 × 10^−2^	‐	‐	None	‐	‐	‐	‐	‐
Lobule VIIIB	L	Linear	4.94	n.s.	1.55 × 10^−2^	‐	‐	Linear	4.81	n.s.	1.59 × 10^−2^	‐	‐
	R	None	‐	‐	‐	‐	‐	None	‐	‐	‐	‐	‐
Lobule IX	L	None	‐	‐	‐	‐	‐	None	‐	‐	‐	‐	‐
	R	None	‐	‐	‐	‐	‐	None	‐	‐	‐	‐	‐
Lobule X	L	Linear	3.52	n.s.	3.68 × 10^−2^	‐	‐	Linear	3.32	n.s.	3.78 × 10^−2^	‐	‐
	R	Linear	2.89	n.s.	4.06 × 10^−2^	‐	‐	Linear	2.79	n.s.	2.93 × 10^−2^	‐	‐

*Note*: The level of significance is 0.0065 after multiple comparisons correction.

Abbreviations: HS, Hemisphere; L, left; n.s., non‐significant; R, right; ‐, not applicable.

**TABLE 6:** Best fit regression model for each SA and parameters for developmental trajectories in boys and girls.

SA	HS	Boys						Girls					
		Best fitting model	Intercept	Site *β*_0_	Age coefficient *β*_1_	Age^2^ coefficient *β*_2_	Age^3^ coefficient *β*_3_	Best fitting model	Intercept	Site *β*_0_	Age coefficient *β*_1_	Age^2^ coefficient *β*_2_	Age^3^ coefficient *β*_3_
Cerebellum	L	Quadratic	1.01 × 10	n.s.	1.55 × 10^−1^	−6.00 × 10^−3^	‐	Quadratic	8.55	n.s.	2.98 × 10^−1^	−1.32 × 10^−2^	‐
	R	Linear	1.06 × 10	n.s.	4.95 × 10^−2^	‐	‐	Cubic	1.13 × 10	n.s.	n.s.	7.43 × 10^−2^	−2.79 × 10^−3^
Lobule I‐II	L	None	‐	‐	‐	‐	‐	None	‐	‐	‐	‐	‐
	R	None	‐	‐	‐	‐	‐	None	‐	‐	‐	‐	‐
Lobule III	L	Linear	1.42 × 10^−1^	n.s.	8.55 × 10^−4^	‐	‐	Linear	1.36 × 10^−1^	n.s.	9.31 × 10^−4^	‐	‐
	R	Linear	1.46 × 10^−1^	n.s.	8.55 × 10^−4^	‐	‐	None	‐	‐	‐	‐	‐
Lobule IV	L	Linear	4.46 × 10^−1^	−1.53 × 10^−2^	2.01 × 10^−3^	‐	‐	Quadratic	3.35 × 10^−1^	n.s.	1.59 × 10^−2^	−7.43 × 10^−4^	‐
	R	Linear	4.42 × 10^−1^	n.s.	2.79 × 10^−3^	‐	‐	Quadratic	3.35 × 10^−1^	n.s.	1.53 × 10^−2^	−6.74 × 10^−4^	‐
Lobule V	L	Linear	7.01 × 10^−1^	n.s.	5.62 × 10^−3^	‐	‐	Linear	6.71 × 10^−1^	n.s.	5.26 × 10^−3^	‐	‐
	R	Linear	7.05 × 10^−1^	n.s.	3.37 × 10^−3^	‐	‐	Quadratic	5.67 × 10^−1^	n.s.	1.88 × 10^−2^	−7.31 × 10^−4^	‐
Lobule VI	L	Quadratic	1.42	n.s.	4.19 × 10^−2^	−1.56 × 10^−3^	‐	Quadratic	1.27	n.s.	5.40 × 10^−2^	−2.40 × 10^−3^	‐
	R	Linear	1.49	n.s.	1.37 × 10^−2^	‐	‐	Quadratic	1.28	n.s.	4.44 × 10^−2^	−1.63 × 10^−3^	‐
Lobule Crus I	L	None	‐	‐	‐	‐	‐	Quadratic	1.88	n.s.	7.12 × 10^−2^	−3.66 × 10^−3^	‐
	R	Linear	2.31	n.s.	9.41 × 10^−3^	‐	‐	Cubic	2.49	n.s.	n.s.	n.s.	−7.85 × 10^−4^
Lobule Crus II	L	None	‐	‐	‐	‐	‐	Quadratic	1.25	n.s.	7.23 × 10^−2^	−3.58 × 10^−3^	‐
	R	None	‐	‐	‐	‐	‐	Quadratic	1.25	n.s.	6.96 × 10^−2^	−3.34 × 10^−3^	‐
Lobule VIIB	L	None	‐	‐	‐	‐	‐	None	‐	‐	‐	‐	‐
	R	Linear	9.02 × 10^−1^	n.s.	4.51 × 10^−3^	‐	‐	None	‐	‐	‐	‐	‐
Lobule VIIIA	L	Linear	1.16	n.s.	8.15 × 10^−3^	‐	‐	Linear	1.03	n.s.	6.92 × 10^−3^	‐	‐
	R	Linear	1.14	n.s.	7.32 × 10^−3^	‐	‐	Linear	1.03	n.s.	7.75 × 10^−3^	‐	‐
Lobule VIIIB	L	Linear	7.46 × 10^−1^	n.s.	5.20 × 10^−3^	‐	‐	Linear	6.45 × 10^−1^	n.s.	4.99 × 10^−3^	‐	‐
	R	Linear	7.27 × 10^−1^	n.s.	6.01 × 10^−3^	‐	‐	Linear	6.55 × 10^−1^	n.s.	4.77 × 10^−3^	‐	‐
Lobule IX	L	Linear	7.15 × 10^−1^	−2.61 × 10^−2^	4.06 × 10^−3^	‐	‐	Quadratic	5.14 × 10^−1^	n.s.	2.97 × 10^−2^	−1.32 × 10^−3^	‐
	R	Linear	7.43 × 10^−1^	−2.47 × 10^−2^	4.22 × 10^−3^	‐	‐	Quadratic	5.86 × 10^−1^	n.s.	1.98 × 10^−2^	−7.98 × 10^−4^	‐
Lobule X	L	None	‐	‐	‐	‐	‐	None	‐	‐	‐	‐	‐
	R	None	‐	‐	‐	‐	‐	None	‐	‐	‐	‐	‐

*Note*: The level of significance is 0.0056 after multiple comparisons correction.

Abbreviations: HS, Hemisphere; L, left; n.s., non‐significant; R, right; ‐, not applicable.

The GMV of lobules bilateral I‐II; the CT of lobules bilateral I‐II, bilateral III, right VIIIB, and bilateral IX; and the SA of lobules right I‐II and left VIIB and bilateral X did not conform to any model. The GMV of lobules bilateral IV (Left: peak = 13.10, 95% CI = 9.18–17.03; Right: peak = 13.07, 95% CI = 9.09–17.05), left VI (peak = 12.81, 95% CI = 9.79–15.84), bilateral crus I (Left: peak = 12.56, 95% CI = 10.32–14.79; Right: peak = 14.47, 95% CI = 10.14–18.81) and II (Left: peak = 11.86, 95% CI = 8.88–14.85; Right: peak = 11.18, 95% CI = 11.13–11.24), the CT of lobule left VIIB (peak = 8.22, 95% CI = 7.03–9.42) and the SA of lobules bilateral IV (Left: peak = 11.71, 95% CI = 8.70–14.73; Right: peak = 12.17, 95% CI = 9.21–15.13), right V (peak = 13.09, 95% CI = 10.42–15.76), left VI (peak = 12.24, 95% CI = 8.94–15.53), left crus I (peak = 9.88, 95% CI = 7.25–12.51) and bilateral crus II (Left: peak = 9.66, 95% CI = 9.39–9.92; Right: peak = 9.83, 95% CI = 9.51–10.14) showed inverted U‐shaped quadratic trajectories. All the other cerebellar subregions of GMV, CT, and SA showed linear growth (Tables 1, 2, 3 and Figures 3, 4, 5).

Previous studies have found that total cerebellar volume showed an inverted U‐shaped trajectory peaking earlier in females than in males during childhood to adolescence.^3^, ^4^ To further investigate sex differences in cerebellar subregion development, we fitted the best curve by sex (Tables 4, 5, 6 and Figures 3, 4, 5). Boys showed increasing linear trajectories for GMV, CT, and surface. In general, boys tended to have a more linear rise in cerebellar subregions, while girls tended to have a more curvilinear development (the GMV of bilateral cerebellum, lobules IV, VI, Curs I, Crus II, left IX, the SA of right cerebellum, bilateral lobules IV, Crus I, Crus II, IX, right V and VI), or a more linear trend for boys than for girls (the GMV of bilateral cerebellum, lobules III, V, VIIB, VIIIA, VIIIB, X, right IX, the CT of the cerebellum, lobules IV, V, VI, Crus I, Crus II, VIIB, VIIIA, X, left VIIIB, the SA of lobules bilateral III, VIIIA, VIIIB, left V), or girls reach peak earlier than boys (the SA of left cerebellum: boys: peak = 12.89, 95% CI = 10.78–15.68, girls: peak = 11.21, 95% CI = 10.56–12.32; the SA of left lobule VI: boys: peak = 13.40, 95% CI = 11.57–16.11, girls: peak = 11.27, 95% CI = 10.57–13.12).

### 3.2 Emotional and behavioral problems and cerebellar subregions and development

**FIGURE 6:**
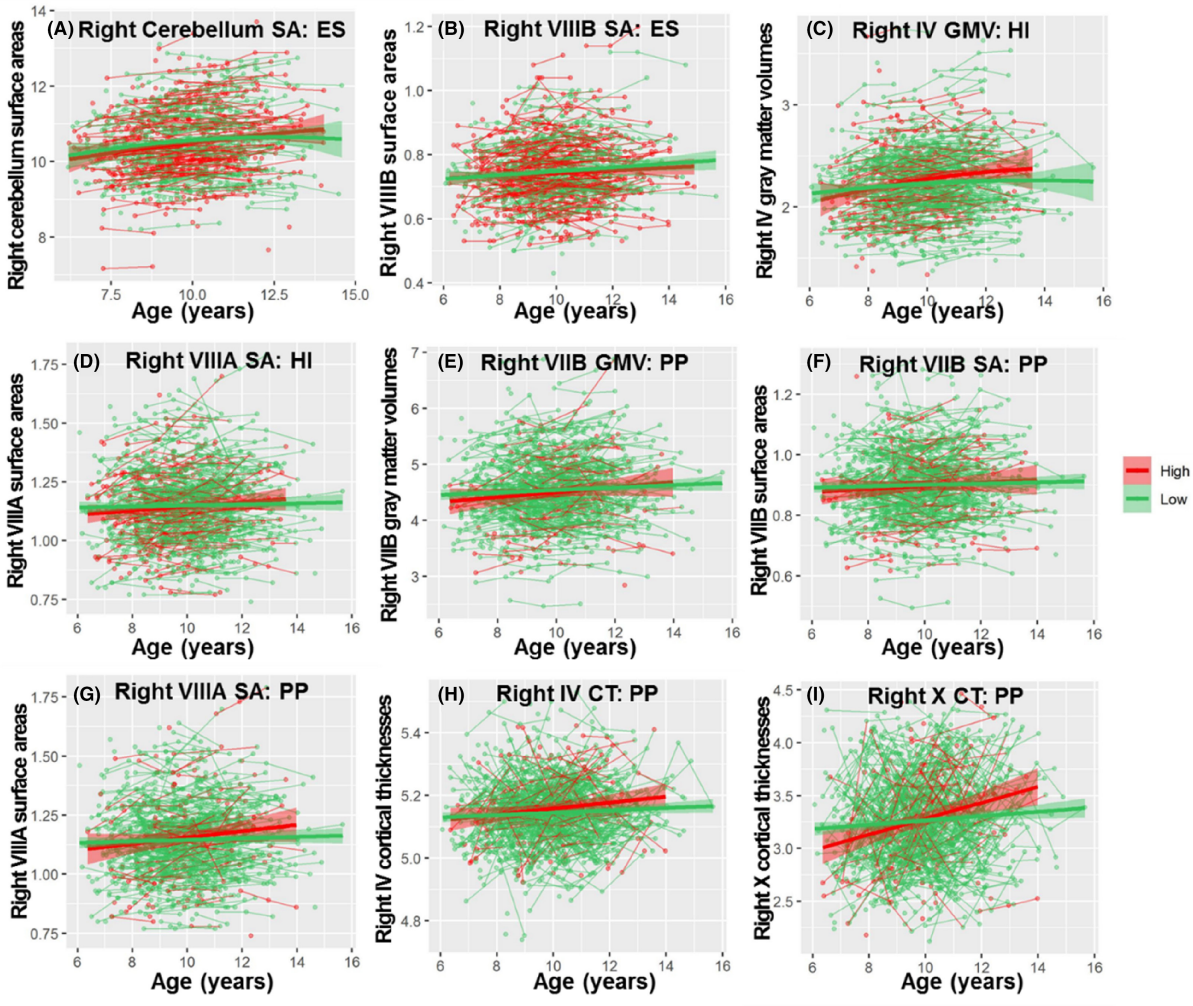
The different developmental trajectories of cerebellar subregion in GMV, CT, SA between relatively high and relatively low‐risk groups of emotional and behavioral problems. (A) The right cerebellum SA moderated by ES, (B) the right VIIIB SA moderated by ES, (C) the right IV GMV moderated by HI, (D) the right VIIIA SA moderated by HI, (E) the right VIIB GMV moderated by PP, (F) the right VIIB SA moderated by PP, (G) the right VIIIA SA moderated by PP, (H) the right IV CT moderated by PP, (I) the right X CT moderated by PP. For visualization purposes, the sample was split into two groups: relatively high (red) and relatively low (green) emotional and behavioral problems. Note that the statistical analyses were performed by using a continuous emotional and behavioral problems score, and adding this score as an interaction term to the best fitting model. Cerebellar GMV, CT and SA (*y*‐axis) by age (*x*‐axis) are shown and the shaded areas represent the 95% confidence intervals. CT, Cortical thickness; ES, Emotional symptoms; GMV, Gray matter volumes; HI, hyperactivity/Inattention; PP, Peer problems; SA, Surface area.

**FIGURE 7:**
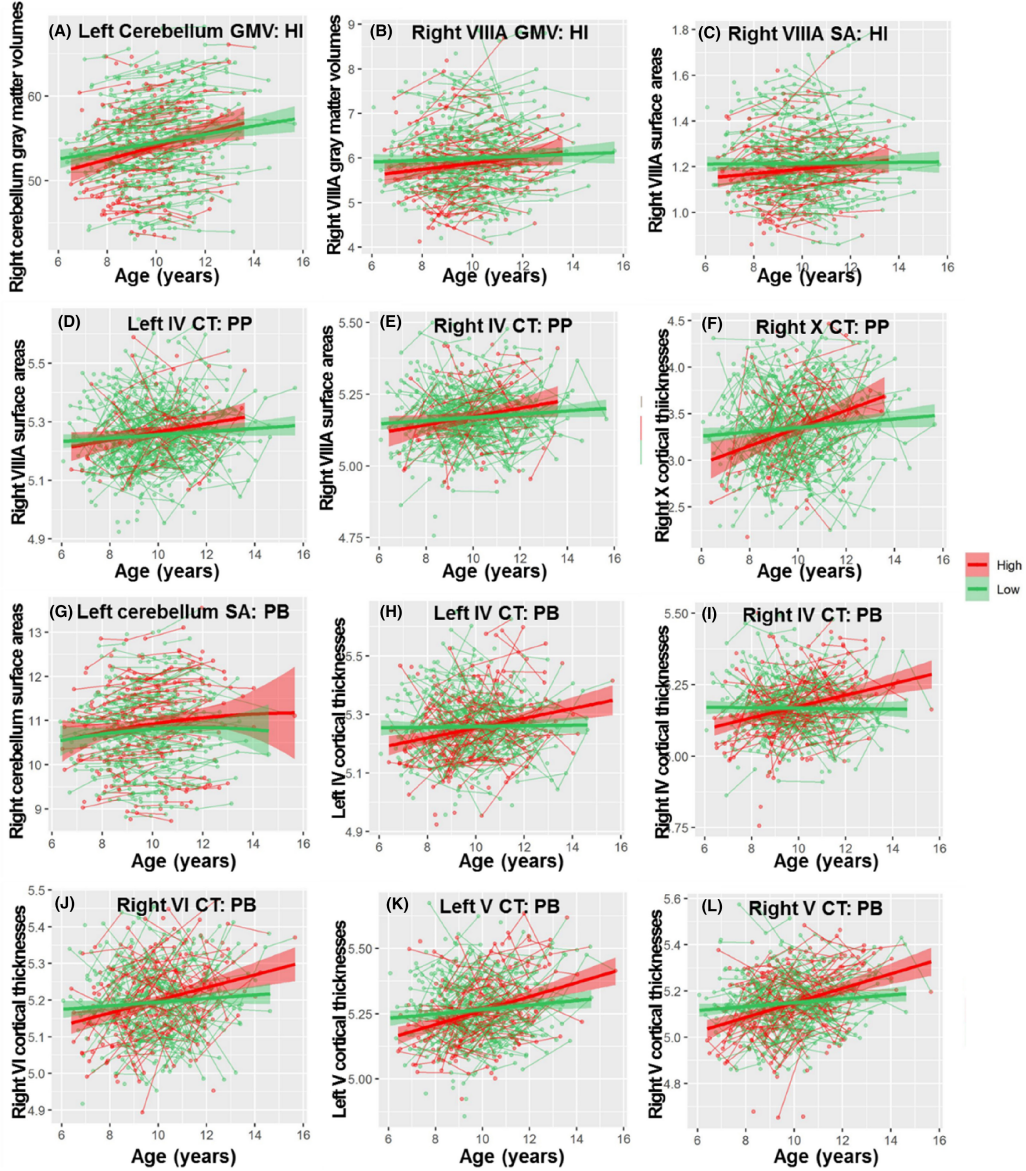
The different developmental trajectories of the cerebellar subregion in GMV, CT, SA between relatively high and relatively low‐risk group of emotional and behavioral problems in boys. (A) The left cerebellum GMV moderated by HI, (B) the right VIIIA GMV moderated by HI, (C) the right VIIIA SA moderated by HI, (D) the left IV CT moderated by PP, (E) the right IV CT moderated by PP, (F) the right X CT moderated by PP, (G) left cerebellum SA moderated by PB, (H) the left IV CT moderated by PB, (I) the right IV CT moderated by PB, (J) the right VI CT moderated by PB, (K) the left V CT moderated by PB, (L) the right V CT moderated by PB. For visualization purposes, the sample was split into two groups: relatively high (red) and relatively low (green) emotional and behavioral problems. Note that the statistical analyses were performed by using a continuous emotional and behavioral problems score, and adding this score as an interaction term to the best fitting model. Cerebellar GMV, CT, and SA (*y*‐axis) by age (*x*‐axis) are shown and the shaded areas represent the 95% confidence intervals. CT, Cortical thickness; GMV, Gray matter volumes; HI, Hyperactivity/Inattention; PB, Prosocial behavior problems; PP, Peer problems; SA, Surface area.

**FIGURE 8:**
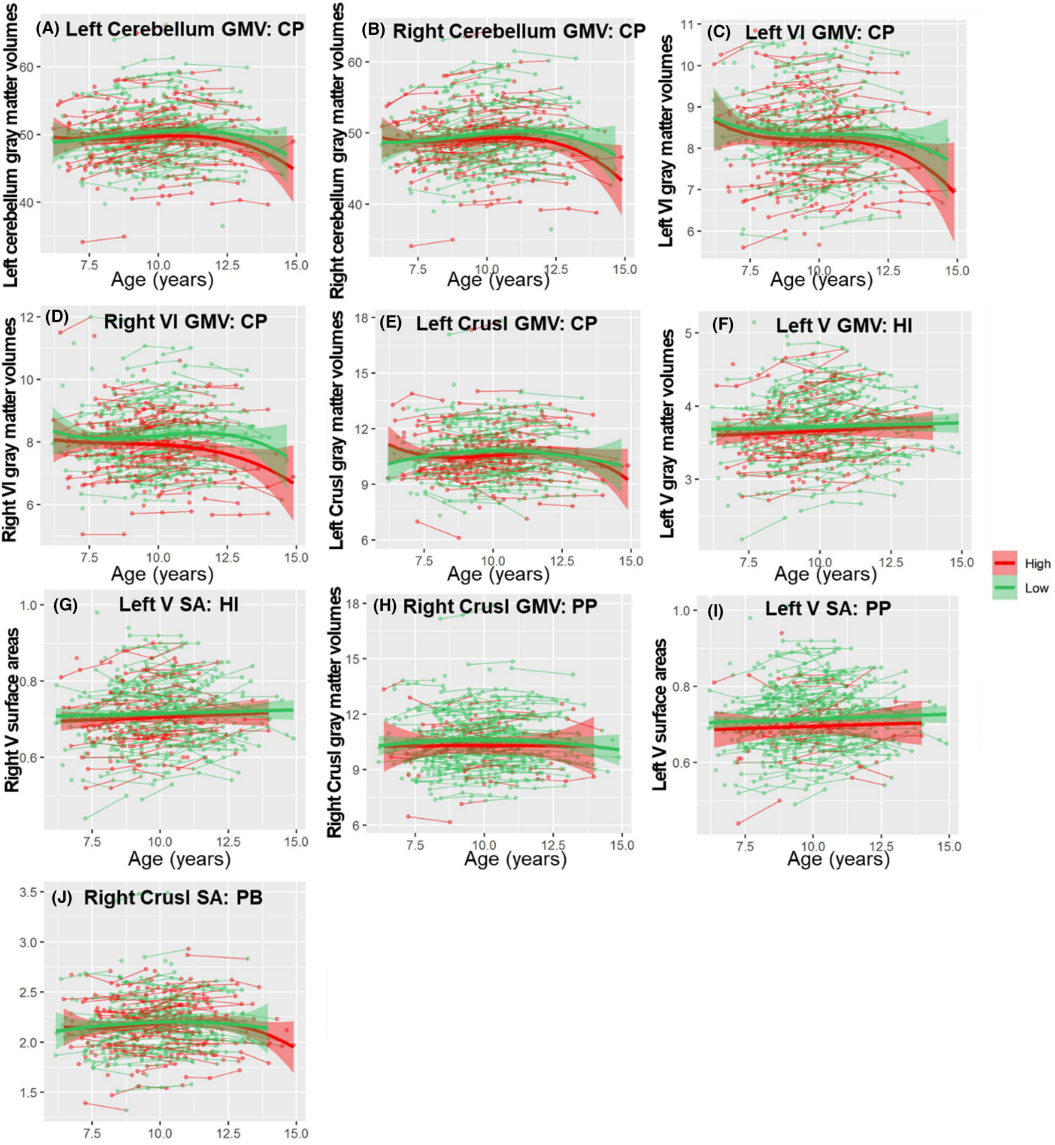
The different developmental trajectories of the cerebellar subregion in GMV, CT, SA between relatively high and relatively low‐risk group of emotional and behavioral problems in girls. (A) The left cerebellum GMV moderated by CP, (B) the right cerebellum GMV moderated by CP, (C) the left VI GMV moderated by CP, (D) the right VI GMV moderated by CP, (E) the left CrusI GMV moderated by CP, (F) the left V GMV moderated by HI, (G) the left V SA moderated by HI, (H) the right CrusI GMV moderated by PP, (I) the left V SA moderated by PP, (J) the right CrusI SA moderated by PB. For visualization purposes, the sample was split into two groups: relatively high (red) and relatively low (green) emotional and behavioral problems. Note that the statistical analyses were performed by using a continuous emotional and behavioral problems score, and adding this score as an interaction term to the best fitting model. Cerebellar GMV, CT and SA (*y*‐axis) by age (*x*‐axis) are shown, and the shaded areas represent the 95% confidence intervals. CP, Conduct problems; CT, Cortical thickness; ES, Emotional symptoms; GMV, Gray matter volumes; HI, hyperactivity/Inattention; PB, Prosocial behavior problems; PP, Peer problems; SA, Surface area.

To investigate how children's emotional and behavioral problems affect the development of cerebellar subregions, we added these continuous scores as an interaction term to the best fitting model (Tables S1–S3 and Figure 6, Tables S4–S6 and Figure 7 for boys and Tables S8 and S9 and Figure 8 for girls).

#### 3.2.1 Emotional symptoms impede expansion of the SA of the cerebellar cortex

Significant age × emotional symptoms interactions were found in the right cerebellar SA (Figure 6A) and the right VIIIB SA (Figure 6B). Group analysis found that the right whole cerebellar SA followed quadratic trajectories both in the high‐ and low‐risk emotional symptoms groups, and the high‐risk group (peak = 14.75, 95% CI = 11.77–16.36) peaked later than the low‐risk group (peak = 13.05, 95% CI = 11.83–14.65). The right VIIIB SA had a weaker linear growth trend in the high‐risk group (*β* = 0.0038, *p* = 2.60 × 10^−4^) than in the low‐risk group (*β* = 0.0068, *p* = 3.67 × 10^−7^). No gender differences were found in the effects of emotions on cerebellar trajectories.

#### 3.2.2 Conduct problems lead to inadequate cerebellar GMV development in girls

Significant age × conduct problems interactions were found in the bilateral cerebellar gray matter (Figure 8A,B), bilateral VI GMV (Figure 8C,D) and left Crus I GMV (Figure 8E), which follow cubic developmental trajectories. As shown in Figure 8A–E, the high‐risk group entered the gradual decline later but also ended earlier, indicating that the GMV of the bilateral cerebellum, bilateral VI and right Crus I were not sufficiently developed in the high‐risk group.

#### 3.2.3 Hyperactivity/inattention delayed the development of cerebellar GMV and SA

Significant age × hyperactivity/inattention interactions were found in the right IV GMV (Figure 6C) and right VIIIA SA (Figure 6D). The group analysis found that the right IV GMV followed quadratic trajectories both in the high‐ and low‐risk hyperactivity/inattention groups, and the high‐risk group (peak = 15.03, 95% CI = 12.10–18.08) peaked later than the low‐risk group (peak = 13.50, 95% CI = 11.39–16.42). The high‐risk group showed a stronger linear increase in the right VIIIA SA (*β* = 0.0142, *p* = 2.82 × 10^−5^) than the low‐risk group (*β* = 0.0059, *p* = 2.75 × 10^−4^).

There were significant differences between age and hyperactivity/inattention in the right cerebellar GMV (Figure 7A), right VIIIA GMV (Figure 7B) and SA (Figure 7C) in boys and the left V GMV (Figure 8F) and SA (Figure 8G) in girls. The group analysis found that the high‐risk group showed stronger linear growth than the low‐risk group (left cerebellar GMV in boys: *β*_high‐risk_ = 0.5436, *p* = 1.33 × 10^−15^, *β*_low‐risk_ = 0.3731, *p* = 2.82 × 10^−5^; right VIIIA GMV in boys: *β*_high‐risk_ = 0.0878, *p* = 3.71 × 10^−4^, *β*_low‐risk_ = 0.0351, *p* = 0.011; right VIIIA SA in boys: *β*_high‐risk_ = 0.0163, *p* = 3.51 × 10^−4^, *β*_low‐risk_ = 0.0041, *p* = 0.089; left V GMV in girls: *β*_high‐risk_ = 0.0523, *p* = 6.42 × 10^−7^, *β*_low‐risk_ = 0.0296, *p* = 3.17 × 10^−8^; left V SA: *β*_high‐risk_ = 0.0087, *p* = 1.49 × 10^−5^, *β*_low‐risk_ = 0.0040, *p* = 1.77 × 10^−4^).

#### 3.2.4 Peer problems disrupt CT growth and SA expansion, resulting in delayed GMV development

Significant age × peer problems interactions were found in the right VIIB GMV (Figure 6E) and SA (Figure 6F), right VIIIA surface (Figure 6G), right IV CT (Figure 6H) and right X CT (Figure 6I). Group analysis found that the high‐risk group showed stronger linear development than the low‐risk group in right VIIB GMV (*β*_high‐risk_ = 0.0459, *p* = 0.0216, *β*_low‐risk_ = 0.020, *p* = 3.55 × 10^−4^) and SA (*β*_high‐risk_ = 0.0055, *p* = 0.139, *β*_low‐risk_ = 0.0020, *p* = 0.073), right VIIIA surface (*β*_high‐risk_ = 0.0077, *p* = 8.01 × 10^−9^, *β*_low‐risk_ = 0.0065, *p* = 0.211), right IV CT (*β*_high‐risk_ = 0.0149, *p* = 2.98 × 10^−4^, *β*_low‐risk_ = 0.0048, *p* = 0.007) and right X CT (*β*_high‐risk_ = 0.0804, *p* = 3.57 × 10^−5^, *β*_low‐risk_ = 0.0286, *p* = 1.15 × 10^−4^).

There were significant differences between age and peer problems in the bilateral IV CT (Figure 7D,E), right X CT (Figure 7F) in boys, and the right Crus I GMV (Figure 8H) and left V SA (Figure 8I) in girls. The group analysis found that the high‐risk group showed stronger linear growth than the low‐risk group (left IV CT in boys: *β*_high‐risk_ = 0.0156, *p* = 0.001, *β*_low‐risk_ = 0.0044, *p* = 0.097; right IV CT in boys: *β*_high‐risk_ = 0.0176, *p* = 0.003, *β*_low‐risk_ = 0.0043, *p* = 0.089; right X CT in boys: *β*_high‐risk_ = 0.0945, *p* = 2.21 × 10^−4^, *β*_low‐risk_ = 0.0307, *p* = 0.003; left V SA: *β*_*high‐risk*_ = 0.0079, *p* = 0.101, *β*_low‐risk_ = 0.0046, *p* = 1.81 × 10^−8^), the right Crus I GMV in girls followed quadratic trajectories both in the high‐ and low‐risk peer problems groups, and the high‐risk group (peak = 15.00, 95% CI = 12.29–18.83) peaked later than the low‐risk group (peak = 13.60, 95% CI = 11.98–16.82).

#### 3.2.5 Prosocial behavior problems impede the expansion of the SA and lead to excessive CT growth in the cerebellum

Significant age × prosocial behavior problem interactions were found in the left cerebellum SA (Figure 7G), bilateral IV CT (Figure 7H,I), bilateral V CT (Figure 7K,L), and right VI CT (Figure 7J) in boys and the right Crus I GMV in girls. Group analysis found that the high‐risk group showed stronger linear development than the low‐risk group in right bilateral IV CT (left: *β*_high‐risk_ = 0.0161, *p* = 6.68 × 10^−5^, *β*_low‐risk_ = 0.0030, *p* = 0.339; right: *β*_high‐risk_ = 0.0183, *p* = 3.01 × 10^−6^, *β*_low‐risk_ = 8.16 × 10^−4^, *p* = 0.777), bilateral V CT (left: *β*_high‐risk_ = 0.0207, *p* = 5.62 × 10^−8^, *β*_low‐risk_ = 0.0088, *p* = 0.007; right: *β*_high‐risk_ = 0.0248, *p* = 2.42 × 10^−7^, *β*_*low‐risk*_ = 0.0077, *p* = 0.017) and right VI CT (*β*_high‐risk_ = 0.0154, *p* = 3.95 × 10^−5^, *β*_low‐risk_ = 0.0053, *p* = 0.056) in boys. The left cerebellar SA in boys and the right Crus I SA in girls followed quadratic trajectories both in the high‐ and low‐risk prosocial behavior groups, and the high‐risk group (boys: peak = 16.13, 95% CI = 11.59–18.28; girls: peak = 11.74, 95% CI = 9.95–14.15) peaked later than the low‐risk group (boys: peak = 11.84, 95% CI = 9.18–14.53; girls: peak = 10.88, 95% CI = 9.19–12.95).

## 4 DISCUSSION

In this study, we explored the developmental trajectories and behavioral correlates of the cerebellum in a 4‐year follow‐up cohort study. This is the first study to map cerebellar development in 24 subregions of children and adolescents using a longitudinal cohort from three dimensions of GMV, CT, and SA. In addition, emotional symptoms, conduct problems, hyperactivity/inattention, peer problems, and prosocial behaviors change cerebellar developmental trajectories from childhood to adolescence.

### 4.1 SA plays a key role in cerebellar development

The GMV consists of CT and SA, and studies based on the cerebral cortex have found that CT and SA have independent genetic origins^10^, ^32^ and different developmental trajectories.^6^, ^11^ Wierenga et al.^11^ examined cerebral cortical change in a large cohort of typically developing children and found that CT and SA development do not reflect the development of GMV, with maximum SA developing later than maximum GMV. In this study, we found that the development trend of the cerebellar cortex in GMV, CT, and SA was not consistent with that of the cerebral cortex: the development trajectory of cerebellar SA mirrored GMV more closely, and maximum SA developed later than maximum GMV. At the whole cerebellar level, GMV and SA both showed inverted U‐shaped quadratic trajectories, and SA peaked almost 2 years earlier than GMV, while the development of CT followed a linear trend. Therefore, it is reasonable to think that the development of SA is pioneering in these three dimensions and may predict the development of the cerebellum. The developmental pattern of the cerebellum may be as follows: after the SA reaches the peak, the increase in GMV is mainly induced by the increase in CT in a short period but soon reaches the peak by the decrease or flattening of the SA and then begins to decline. This development trend is also reflected in the four subregions of lobule IV, lobule VI, Crus I, and Crus II. In addition, the relatively similar functional developmental maps of GMV and SA suggest that the corresponding functions of GMV are more determined by SA and that CT has a weaker relationship with behavior.

Why was the developmental trajectory of gray matter determined primarily by the changes in SA? The human and animal anatomy shows that although the cerebellum accounts for only 10% of the total brain volume, the mature cerebellum contains more than half of all neurons; therefore, the cerebellum folds into this compact space like an accordion (a narrow band 10 cm wide and nearly a meter long).^33^ Compared to other mammals, it is one of the most markedly expanded brain regions in humans,^34^ and the SA has increased more than 1000‐fold over the course of mammalian evolution without a comparable increase in CT, suggesting that cortical expansion arises from proliferation kinetics, which increase the number of radial columnar units without significantly changing the number of neurons within each unit.^9^ Thus, it is effective to expand the distribution space of neurons by increasing the SA or number of folds within a limited space.

### 4.2 Boys and girls show different developmental trajectories

This study identified three types of differences in cerebellar development trends between boys and girls. First, both girls and boys showed inverted U‐shaped quadratic trajectories in the SA of the left whole cerebellum and left lobule VI, and girls peaked earlier than boys. This is very similar to previous findings. Tiemeier et al.^3^ found that males and females followed the same developmental trajectories, while females peaked earlier than males. Second, boys tended to have a more linear rise in cerebellar subregions, while girls tended to have a more curvilinear development. Third, boys showed stronger linear growth than girls.

Why do boys and girls show these three different patterns of cerebellar development? This may be caused by differences in age distribution. In Tiemeier et al.'s study, the age distribution was 5–24 years, and they found that girls peaked at 11.8 years and boys peaked at 15.5 years. In our study, the age distribution was 6 to 15 years, an age range when cerebellar development in boys is still rising in the first half of the inverted U‐shaped curve, so we can only observe a linear rise. Girls are more likely to be identified as inverted U‐shaped because they peak earlier. For example, similar results were found in Tiemeier et al.'s study; corps medullare was found to be the area that reached the peak at the latest. Therefore, only girls were identified to reach the peak at the age of 17.2, while boys were always in a linear rise.^3^ On the other hand, in the case that no peak was detected for both boys and girls in this study, both showed a linear increase. Since girls were closer to the peak, the linear increasing trend would be weaker than that of boys. In addition, we found that cerebellar SA peaks earlier than GMV, so despite the narrow age distribution in this study, we found that both boys and girls peaked in the SA of the left whole cerebellum and left lobule VI, and girls peaked earlier. Overall, the differences observed in the developmental trajectories between boys and girls can be attributed to the gender difference in brain maturation.

There were few research studies showing gender difference in brain development particularly in cortical and subcortical structures, and the difference might be due to effect of puberty.^35^, ^36^, ^37^ Using indirect clinical data, they found that puberty hormones or sex chromosome differences may play a role in this process.^35^ However, direct evidence of the hormonal effects of puberty on the anatomy of the developing human brain is lacking. Since then, some studies also found that the cortical and subcortical structures of the brain in girls tend to peak earlier compared to those in boys.^28^, ^36^, ^37^, ^38^ Among them, Hu et al.^37^ confirmed the relationship between sex differences in the volume of the medial temporal lobe and puberty by using puberty scores, and Goddings et al.^36^ demonstrated that puberty affected sex differences in subcortical brain development using the Tanner stage. So far, we have found no studies that link sex differences in cerebellar development to puberty, and unfortunately, we did not have data on puberty, so we cannot confirm this question. It is hoped that future studies will collect data on puberty to confirm whether the sex difference in cerebellar development is due to puberty.

### 4.3 Emotional and behavioral problems impede cerebellar development

This is the first comprehensive study to examine the effects of emotional and behavioral problems on the developmental trajectories of cerebellar GMV, CT, and SA subregions from childhood to adolescence. We found that emotional and behavioral problems changed the developmental trajectory of the cerebellum in different ways.

First, emotional symptoms impede the expansion of the SA of the cerebellar cortex. We found that the high‐risk group for emotional problems peaked on the right cerebellar SA approximately 1.7 years later than the low‐risk group and showed a stronger linear trend in the right VIIIB SA. This indicates that emotional problems lead to delayed development of the cerebellar cortex, which is mainly reflected in the expansion of the cortical SA. Previous studies of mood disorders such as bipolar disorder and depression have found that cerebellar GMV decreases,^39^, ^40^, ^41^ and this study provides a procedural explanation for the formation of these results. Previous studies have found that the increase in cortical GMV from childhood to adolescence is mainly dependent on SA expansion,^42^, ^43^ and this expansion appears to be more important in the cerebellum.^33^ In other words, the smaller cerebellar GMV found in previous studies based on mood disorders^39^, ^40^, ^41^ may be caused by the delayed expansion of cortical SA caused by emotional symptoms, which suggests that cortical SA expansion of the left cerebellum and lobule VIIIB delay may be important early predictors of mood disorders.

Second, conduct problems lead to inadequate cerebellar GMV development in girls. We found that conduct problems regulated the development of GMV of the bilateral cerebellum, bilateral VI, and left Crus I that they follow cubic developmental trajectories in girls, and the high‐risk group of conduct problems entered the gradual decline later but also ended earlier, indicating that the GMV in these areas may not be sufficiently developed in the high‐risk group for conduct problems. Previous studies have found that a delay in cortical maturation in several areas related to decision‐making, morality, and empathy is a significant cause of conduct problems^44^ but rarely involves the cerebellum. However, this study further found that the high‐risk group of conduct problems not only matured later but also had a short developmental cycle and did not achieve full development. Therefore, future studies may consider intervening in the mature stage to fully develop the affected cerebellar subregions. Studies based on functional brain networks have found that conduct problems are mainly reflected in dysfunction of the default network,^45^, ^46^, ^47^ while underdeveloped brain areas found in the cerebellum in this study are mainly concentrated in lobule VI and Crus I, which are also part of the default network.^48^, ^49^ This study also provides a new explanation for the disordered default network of conduct problems, which may be caused by inadequate structural development during childhood and adolescence.

Why do conduct problems cause cerebellar GMV underdevelopment in girls but not boys? According to previous research, in general, girls tended to have fewer behavioral problems, and high‐risk girls may have more behavioral problems than high‐risk boys when assessed in multiple domains.^50^ In addition, girls with conduct problems exhibited greater electrodermal responding.^51^ It has been proposed that girls may require a greater loading of neurobiological or psychosocial risk factors to develop conduct problems, and this reflected in greater gray matter volume reductions (e.g. the left posterior insula) in girls with conduct problems compared with boys.^52^ Therefore, it is because high‐risk girls tend to have greater impairments that they are more likely than boys to exhibit inadequate development in the cerebellum.

Third, hyperactivity/inattention delayed the development of cerebellar GMV and SA. ADHD is characterized by a delay in cortical maturation^53^, ^54^, ^55^; however, they only found a reduction in the volume of the vermis and a faster growth rate of the left medulla, not a developmental delay in the cerebellum.^17^, ^19^ In this study, we observed for the first time the lag of cerebellar gray matter development in the high‐risk group of hyperactivity/inattention problems and mainly reflected in the peak GMV in the right IV region approximately 1.5 years later than in the low‐risk group. Why did we observe this lag that was not found in previous studies? This may be due to the larger sample size and finer subdivision. The human cerebellum has a protracted developmental timeline compared with the neocortex, so it is more difficult to reach its peak during childhood and adolescence. Therefore, it is easier to observe linear changes in the cerebellum, such as the faster development rate of the left medulla observed by Shaw et al.,^19^ and the same phenomenon in the lobule right VIIIA, V was also observed in this study, which may be caused by the fact that the low‐risk group is closer to the peak value and the development speed slows down earlier. The possibility of capturing peak cerebellar development was also raised by the use of a large longitudinal cohort study with finely delineated cerebellar subregions and multidimensional measurements of GMV, CT, and SA in this study.

In terms of gender differences, boys at high risk of hyperactivity/inattention showed developmental delays in the left cerebellar GMV, right VIIIA GMV and SA in boys, while high risk girls only in the left V GMV and SA. Both V and VIII have been shown to be associated with motor and somatosensory representations in a meta‐analysis of neuroimaging studies.^56^ However, in terms of the extent of damage, high‐risk girls suffered damage only to the left V lobule, while boys suffered more damage, including the whole left cerebellum and the right VIIIA lobule. This may be related to the higher prevalent and more severe symptoms in boys compared to girls.^57^, ^58^

Fourth, peer behavior problems disrupt CT growth and SA expansion, resulting in delayed GMV development. At present, no studies have explored the relationship between peer problems and cerebellar development. Therefore, we can only refer to the results of ASD research with social impairment at its core. A study based on a mouse model found that a decrease in Purkinje cell functioning leads to abnormal social behaviors.^59^ Three major cerebellar abnormalities were observed in ASD patients: Purkinje cell depletion, cerebellar volume reduction, and disruption of feedback pathways between the cerebellum and brain regions. The latter two may also be by‐products of Purkinje cell depletion,^60^ and pathological changes are evident in the superior peduncles of the cerebellum in children with ASD.^61^ This study also found that lobules IV, X, VIIB, and V mainly showed differences in CT or SA development speed, while Crus I only showed a significant development delay (approximately 1.5 years). In this study, we again demonstrate that the cerebellar Crus I may be the core region of social dysfunction and further show that this reduction in cerebellar volume is mainly caused by delayed GMV development due to abnormal development of CT and SA.

In addition, differences were found in the IV and X lobules in high‐risk boys and Crus I and V in high‐risk girls. The study found that girls were more likely to seek non‐parental relationships and were more likely to be influenced by their peers,^62^ and boys were more likely to stress their independence in relationships and feel less connected to their peers.^63^ Therefore, girls' peer relationship may be more reflected in communication, while boys' peer relationship may be more reflected in problem behaviors. Based on the previous meta‐analysis, IV and X were related to motor and sensorimotor, while Crus I and V were involved in language and cognition.^56^ This may account for the differences of peer behavior problems between boys and girls in this study.

Fifth, prosocial behavior problems impede the expansion of the SA and lead to excessive CT growth in the cerebellum. The neuronal mechanisms underlying prosocial behavior are still elusive. Recently, Wang et al.^64^ identified a causal role of the basal forebrain in the control of prosocial behavior via inhibitory projections that disinhibit midbrain ventral tegmental area (VTA) dopamine neurons. Carta et al.^65^ found that monosynaptic connections from the fastigial nucleus of the cerebellum directly modulate VTA activity, and dysfunction of the cerebellum–VTA connection could contribute to the pathogenesis of diseases in which the dopaminergic system is dysregulated.^66^ However, the above studies are based on the speculation of the mouse model, and there is no direct evidence for how the cerebellum directly plays a role in prosocial behavior. In this study, we found that prosocial behavior problems mainly affect cerebellar development from two aspects: first, they hinder cerebellar expansion during the critical period of cortical SA expansion; meanwhile, they also cause the rapid growth of CT, resulting in the disorder of cerebellar structure development, and thus aggravate functional problems.

This study found that prosocial behavior problems mainly affected the bilateral IV, V, right VI lobules, and the left cerebellum in boys, while only influencing Crus I lobule in girls. This may be related to the different characteristics of prosocial behavior problems in boys and girls. Previous studies showed that prosocial behavior in girls was more associated with emotional symptoms (such as depression),^67^ while prosocial behavior in boys is more related to externalizing behaviors.^68^ Emotional numbing occurred when the frontal cortex and anterior cingulate over‐regulated the responses of the amygdala and insula, and the Crus I played an important role in this emotional overmodulation.^69^ The cerebellum, especially the midline cerebellar structure (vermis, fastigialnucleus), has been shown to be related to externalizing behavior.^70^

In this study, we found environment and biology are important influencing factors. First, gender, as the biological factor mainly considered in this study, regulates the developmental trajectories of different subregions of the cerebellum. Overall, the cerebellum of girls matured earlier, but unfortunately, due to the lack of puberty data, it could not be determined whether it was related to puberty. Second, children's emotional and behavioral problems, as important influencing factors, also affect the developmental trajectory of children's cerebellum to varying degrees, resulting in a variety of developmental trajectory abnormalities including developmental delay, inadequate and disorder. Third, the influence of children's emotional and behavioral problems on the developmental trajectories of cerebellar subregions was also regulated by gender. Due to the different characteristics and mechanisms of emotional and behavioral problems in boys and girls, the influence of emotional behavior problems on the developmental trajectories of cerebellar subregions was also different, respectively. In summary, the developmental trajectories of cerebellar subregions were the result of a combination of biological and environmental influences.

## 5 LIMITATIONS

The study has some shortcomings. First, the sample involved only children/adolescents aged 6–15 years. The human cerebellum has a protracted developmental timeline compared with the neocortex; therefore, it may take a longer age span to capture the peak of cerebellar development.^14^ In Tiemeier et al.'s^3^ study, the age distribution was 5–24 years, and they found that the total cerebellar volume, inferior posterior lobe, superior posterior lobe, anterior lobe, and corps medullare peaked at 11.1–17.2 years of age. In this study, curvilinear development was found only in some brain regions, while linear development was found in other brain regions, which may be related to the finer division and of the brain regions and more multidimensional measures. This can be further explored to determine which brain regions develop curvilinearly and linearly by expanding the age range in future studies. Second, the SA in this study was derived from the ratio of GMV to CT. Due to the limitations of current MRI measurement and computational techniques, there is no published literature indicating a better method to directly measure the SA of the cerebellar cortex. Therefore, this is a compromise that has to be adopted and is theoretically sound, and this computational method has long been applied in many studies.^10^, ^71^ Finally, in this study, we found that there were gender differences in both the trajectories of cerebellar development and the effects of emotional behavior problems on them. Although studies based on cortical and subcutaneous structures have found that this may be due to the effects of puberty,^35^, ^36^, ^37^ no studies have explored the relationship between cerebellar development trajectory and puberty, and this study was unable to examine the relationship due to the lack of puberty data. Therefore, it is hoped that future studies can solve this problem by collecting data on puberty.

## 6 CONCLUSIONS

In this study, we first mapped the developmental trajectories of gray matter, CT, and SA in cerebellar subregions from childhood to adolescence based on a longitudinal cohort study, and then we investigated in detail how emotional and behavioral problems can cause involvement damage by changing the cerebellar development trajectory. The results showed that emotional symptoms impede the expansion of the SA of the cerebellar cortex; conduct problems lead to inadequate cerebellar GMV development in girls; hyperactivity/inattention delays the development of cerebellar GMV and SA; peer behavior problems disrupt CT growth and SA expansion, resulting in delayed GMV development; and prosocial behavior problems impede the expansion of the SA and lead to excessive CT growth in the cerebellum. This study provides the first evidence for how emotional and behavioral problems affect the dynamic development of GMV, CT and SA, which provides an important basis and guidance for the prevention and intervention of cognitive and emotional behavioral problems in the future.

## FUNDING INFORMATION

This study has been supported by the STI 2030—Major Projects 2021ZD0200500.

## CONFLICT OF INTEREST STATEMENT

Dr. Yong is an Editorial Board member of CNS Neuroscience and Therapeutics and a co‐author of this article. To minimize bias, they were excluded from all editorial decision‐making related to the acceptance of this article for publication.

## Supporting information


Tables S1–S9

## Data Availability

The raw data supporting the conclusions of this article were from the Children School Functions and Brain Development Project (CBD, Beijing Cohort), which will be soon made public.
