# Supplementary material for: Emotional and behavioral problems change the development of cerebellar gray matter volume, thickness, and surface area from childhood to adolescence: A longitudinal cohort study
Source: CNS Neurosci Ther. 2023 Jun 8;29(11):3528–48. doi: 10.1111/cns.14286 (PMC10580368; doi:10.1111/cns.14286)
Supplement: Supplementary file 1 — Tables S1–S9 [file CNS-29-3528-s001.docx]

**Supplemental Information**

**Supplemental Tables**

Table S1 Model parameters for fixed effects when including level of emotional and behavioural problems in the best fitting models for whole cerebellum and cerebeller subregion gray matter volumes.

| Gray matter volume | HS | Best fitting model | EBP Scales | Intercept | Site | Gender coefficient  β1 | Age coefficient  β2 | Age^2^ coefficient  β3 | EBP | Age×EBP coefficient  β_5_ | Age^2^×EBP coefficient  β_6_ |
| --- | --- | --- | --- | --- | --- | --- | --- | --- | --- | --- | --- |
| Cerebellum | L | Quadratic | ES | 4.20×10 | n.s. | 4.29 | 1.37 | -5.06×10^-2^ | n.s. | n.s. | n.s. |
|  |  |  | CP | 4.22×10 | n.s. | 4.29 | 1.34 | -5.07×10^-2^ | n.s. | n.s. | n.s. |
|  |  |  | HI | 4.35×10 | n.s. | 4.29 | 1.10 | -4.00×10^-2^ | n.s. | n.s. | n.s. |
|  |  |  | PP | 4.18×10 | n.s. | 4.30 | 1.42 | -5.43×10^-2^ | n.s. | n.s. | n.s. |
|  |  |  | PB | 4.28×10 | n.s. | 4.29 | n.s. | n.s. | n.s. | n.s. | n.s. |
|  | R | Quadratic | ES | 4.13×10 | n.s. | 4.62 | 1.40 | -5.04×10^-2^ | n.s. | n.s. | n.s. |
|  |  |  | CP | 4.23×10 | n.s. | 4.62 | 1.20 | -4.22×10^-2^ | n.s. | n.s. | n.s. |
|  |  |  | HI | 4.24×10 | n.s. | 4.62 | 1.21 | -4.35×10^-2^ | n.s. | n.s. | n.s. |
|  |  |  | PP | 4.19×10 | n.s. | 4.63 | 1.32 | -4.98×10^-2^ | n.s. | n.s. | n.s. |
|  |  |  | PB | 3.93×10 | n.s. | 4.63 | 1.73 | n.s. | n.s. | n.s. | n.s. |
| Lobule Ⅰ-Ⅱ | L | None | ES | 2.56×10^-2^ | n.s. | 3.16×10^-3^ | n.s. | - | n.s. | - | - |
|  |  |  | CP | 2.55×10^-2^ | n.s. | 3.19×10^-3^ | n.s. | - | n.s. | - | - |
|  |  |  | HI | 2.56×10^-2^ | n.s. | 3.19×10^-3^ | n.s. | - | n.s. | - | - |
|  |  |  | PP | 2.54×10^-2^ | n.s. | 3.19×10^-3^ | n.s. | - | n.s. | - | - |
|  |  |  | PB | 2.62×10^-2^ | n.s. | 3.13×10^-3^ | n.s. | - | n.s. | - | - |
|  | R | None | ES | 3.68×10^-2^ | n.s. | 3.45×10^-3^ | n.s. | - | n.s. | - | - |
|  |  |  | CP | 3.69×10^-2^ | n.s. | 3.42×10^-3^ | n.s. | - | n.s. | - | - |
|  |  |  | HI | 3.73×10^-2^ | n.s. | 3.45×10^-3^ | n.s. | - | n.s. | - | - |
|  |  |  | PP | 3.72×10^-2^ | n.s. | 3.46×10^-3^ | n.s. | - | n.s. | - | - |
|  |  |  | PB | 3.71×10^-2^ | n.s. | 3.44×10^-3^ | n.s. | - | n.s. | - | - |
| Lobule Ⅲ | L | Linear | ES | 4.95×10^-1^ | n.s. | 5.00×10^-2^ | 4.71×10^-3^ | - | n.s. | n.s. | - |
|  |  |  | CP | 4.87×10^-1^ | n.s. | 4.98×10^-2^ | 5.13×10^-3^ | - | n.s. | n.s. | - |
|  |  |  | HI | 5.02×10^-1^ | n.s. | 5.02×10^-2^ | 4.06×10^-3^ | - | n.s. | n.s. | - |
|  |  |  | PP | 4.92×10^-1^ | n.s. | 4.99×10^-2^ | 4.87×10^-3^ | - | n.s. | n.s. | - |
|  |  |  | PB | 5.29×10^-1^ | n.s. | 4.99×10^-2^ | n.s. | - | n.s. | n.s. | - |
|  | R | Linear | ES | 4.81×10^-1^ | n.s. | 5.49×10^-2^ | 4.18×10^-3^ | - | n.s. | n.s. | - |
|  |  |  | CP | 4.72×10^-1^ | n.s. | 5.46×10^-2^ | 4.54×10^-3^ | - | n.s. | n.s. | - |
|  |  |  | HI | 4.94×10^-1^ | n.s. | 5.51×10^-2^ | n.s. | - | n.s. | n.s. | - |
|  |  |  | PP | 4.85×10^-1^ | n.s. | 5.53×10^-2^ | 3.90×10^-3^ | - | n.s. | n.s. | - |
|  |  |  | PB | 4.84×10^-1^ | n.s. | 5.46×10^-2^ | n.s. | - | n.s. | n.s. | - |
| Lobule Ⅳ | L | Quadratic | ES | 1.92 | -6.18×10^-2^ | 2.12×10^-1^ | 4.94×10^-2^ | n.s. | n.s. | n.s. | n.s. |
|  |  |  | CP | 1.98 | -6.18×10^-2^ | 2.12×10^-1^ | n.s. | n.s. | n.s. | n.s. | n.s. |
|  |  |  | HI | 1.83 | -6.19×10^-2^ | 2.12×10^-1^ | 6.28×10^-2^ | n.s. | n.s. | n.s. | n.s. |
|  |  |  | PP | 1.90 | -6.18×10^-2^ | 2.13×10^-1^ | n.s. | n.s. | n.s. | n.s. | n.s. |
|  |  |  | PB | 2.09 | -6.18×10^-2^ | 2.13×10^-1^ | n.s. | n.s. | n.s. | n.s. | n.s. |
|  | R | Quadratic | ES | 1.76 | -5.53×10^-2^ | 2.39×10^-1^ | 6.79×10^-2^ | -2.59×10^-3^ | n.s. | n.s. | n.s. |
|  |  |  | CP | 1.67 | -5.52×10^-2^ | 2.40×10^-1^ | 8.87×10^-2^ | -3.73×10^-3^ | n.s. | n.s. | n.s. |
|  |  |  | HI | 1.66 | -5.49×10^-2^ | 2.41×10^-1^ | 9.62×10^-2^ | -4.24×10^-3^ | n.s. | n.s. | 6.97×10^-4^ |
|  |  |  | PP | 1.78 | -5.54×10^-2^ | 2.40×10^-1^ | 6.63×10^-2^ | -2.61×10^-3^ | n.s. | n.s. | n.s. |
|  |  |  | PB | 1.73 | -5.54×10^-2^ | 2.40×10^-1^ | n.s. | n.s. | n.s. | n.s. | n.s. |
| Lobule Ⅴ | L | Linear | ES | 3.39 | n.s. | 2.44×10^-1^ | 3.68×10^-2^ | - | n.s. | n.s. | - |
|  |  |  | CP | 3.41 | n.s. | 2.44×10^-1^ | 3.47×10^-2^ | - | n.s. | n.s. | - |
|  |  |  | HI | 3.46 | n.s. | 2.44×10^-1^ | 2.92×10^-2^ | - | n.s. | n.s. | - |
|  |  |  | PP | 3.42 | n.s. | 2.44×10^-1^ | 3.41×10^-2^ | - | n.s. | n.s. | - |
|  |  |  | PB | 3.32 | n.s. | 2.45×10^-1^ | 4.17×10^-2^ | - | n.s. | n.s. | - |
|  | R | Linear | ES | 3.20 | n.s. | 2.99×10^-1^ | 2.72×10^-2^ | - | n.s. | n.s. | - |
|  |  |  | CP | 3.19 | n.s. | 2.99×10^-1^ | 2.79×10^-2^ | - | n.s. | n.s. | - |
|  |  |  | HI | 3.22 | n.s. | 2.99×10^-1^ | 2.55×10^-2^ | - | n.s. | n.s. | - |
|  |  |  | PP | 3.21 | n.s. | 3.00×10^-1^ | 2.63×10^-2^ | - | n.s. | n.s. | - |
|  |  |  | PB | 3.15 | n.s. | 3.03×10^-1^ | n.s. | - | n.s. | n.s. | - |
| Lobule Ⅵ | L | Quadratic | ES | 6.69 | n.s. | 4.74×10^-1^ | 2.75×10^-1^ | -1.09×10^-2^ | n.s. | n.s. | n.s. |
|  |  |  | CP | 6.67 | n.s. | 4.74×10^-1^ | 2.85×10^-1^ | -1.18×10^-2^ | n.s. | n.s. | n.s. |
|  |  |  | HI | 6.66 | n.s. | 4.72×10^-1^ | 2.87×10^-1^ | -1.20×10^-2^ | n.s. | n.s. | n.s. |
|  |  |  | PP | 6.53 | n.s. | 4.73×10^-1^ | 3.06×10^-1^ | -1.26×10^-2^ | n.s. | n.s. | n.s. |
|  |  |  | PB | 6.54 | n.s. | 4.75×10^-1^ | n.s. | n.s. | n.s. | n.s. | n.s. |
|  | R | Linear | ES | 7.33 | n.s. | 3.73×10^-1^ | 7.34×10^-2^ | - | n.s. | n.s. | - |
|  |  |  | CP | 7.43 | n.s. | 3.75×10^-1^ | 6.35×10^-2^ | - | n.s. | n.s. | - |
|  |  |  | HI | 7.43 | n.s. | 3.76×10^-1^ | 6.42×10^-2^ | - | n.s. | n.s. | - |
|  |  |  | PP | 7.36 | n.s. | 3.73×10^-1^ | 6.93×10^-2^ | - | n.s. | n.s. | - |
|  |  |  | PB | 7.22 | n.s. | 3.75×10^-1^ | 8.27×10^-2^ | - | n.s. | n.s. | - |
| Lobule  Crus I | L | Quadratic | ES | 8.73 | n.s. | 9.13×10^-1^ | 3.51×10^-1^ | -1.45×10^-2^ | n.s. | n.s. | n.s. |
|  |  |  | CP | 9.04 | n.s. | 9.16×10^-1^ | 2.94×10^-1^ | -1.23×10^-2^ | n.s. | n.s. | n.s. |
|  |  |  | HI | 9.43 | n.s. | 9.14×10^-1^ | n.s. | n.s. | n.s. | n.s. | n.s. |
|  |  |  | PP | 8.77 | n.s. | 9.19×10^-1^ | 3.32×10^-1^ | -1.32×10^-2^ | n.s. | n.s. | n.s. |
|  |  |  | PB | 9.03 | n.s. | 9.15×10^-1^ | n.s. | n.s. | n.s. | n.s. | n.s. |
|  | R | Quadratic | ES | 8.74 | n.s. | 1.02 | 3.16×10^-1^ | -1.18×10^-2^ | n.s. | n.s. | n.s. |
|  |  |  | CP | 8.90 | n.s. | 1.02 | 2.82×10^-1^ | n.s. | n.s. | n.s. | n.s. |
|  |  |  | HI | 8.87 | n.s. | 1.02 | 2.73×10^-1^ | n.s. | n.s. | n.s. | n.s. |
|  |  |  | PP | 8.77 | n.s. | 1.02 | 3.11×10^-1^ | -1.20×10^-2^ | n.s. | n.s. | n.s. |
|  |  |  | PB | 8.85 | n.s. | 1.02 | n.s. | n.s. | n.s. | n.s. | n.s. |
| Lobule  Crus II | L | Quadratic | ES | 6.22 | n.s. | 4.66×10^-1^ | 2.80×10^-1^ | -1.17×10^-2^ | n.s. | n.s. | n.s. |
|  |  |  | CP | 6.15 | n.s. | 4.69×10^-1^ | 3.01×10^-1^ | -1.31×10^-2^ | n.s. | n.s. | n.s. |
|  |  |  | HI | 5.77 | n.s. | 4.67×10^-1^ | 3.67×10^-1^ | -1.62×10^-2^ | n.s. | n.s. | n.s. |
|  |  |  | PP | 5.86 | n.s. | 4.68×10^-1^ | 3.53×10^-1^ | -1.56×10^-2^ | n.s. | n.s. | n.s. |
|  |  |  | PB | 6.27 | n.s. | 4.68×10^-1^ | n.s. | n.s. | n.s. | n.s. | n.s. |
|  | R | Quadratic | ES | 6.00 | n.s. | 6.38×10^-1^ | 3.76×10^-1^ | -1.71×10^-2^ | n.s. | n.s. | n.s. |
|  |  |  | CP | 6.02 | n.s. | 6.41×10^-1^ | 3.74×10^-1^ | -1.72×10^-2^ | n.s. | n.s. | n.s. |
|  |  |  | HI | 6.10 | n.s. | 6.38×10^-1^ | 3.51×10^-1^ | -1.60×10^-2^ | n.s. | n.s. | n.s. |
|  |  |  | PP | 5.98 | n.s. | 6.42×10^-1^ | 3.71×10^-1^ | -1.66×10^-2^ | n.s. | n.s. | n.s. |
|  |  |  | PB | 5.28 | n.s. | 6.38×10^-1^ | n.s. | n.s. | n.s. | n.s. | n.s. |
| Lobule ⅦB | L | Linear | ES | 4.06 | n.s. | 3.62×10^-1^ | 2.31×10^-2^ | - | n.s. | n.s. | - |
|  |  |  | CP | 4.10 | n.s. | 3.63×10^-1^ | n.s. | - | n.s. | n.s. | - |
|  |  |  | HI | 4.20 | n.s. | 3.65×10^-1^ | n.s. | - | n.s. | n.s. | - |
|  |  |  | PP | 4.12 | n.s. | 3.63×10^-1^ | n.s. | - | n.s. | n.s. | - |
|  |  |  | PB | 3.87 | n.s. | 3.63×10^-1^ | n.s. | - | n.s. | n.s. | - |
|  | R | Linear | ES | 4.11 | n.s. | 4.25×10^-1^ | 2.83×10^-2^ | - | n.s. | n.s. | - |
|  |  |  | CP | 4.17 | n.s. | 4.28×10^-1^ | 2.17×10^-2^ | - | n.s. | n.s. | - |
|  |  |  | HI | 4.13 | n.s. | 4.31×10^-1^ | 2.73×10^-2^ | - | n.s. | n.s. | - |
|  |  |  | PP | 4.25 | n.s. | 4.25×10^-1^ | n.s. | - | -8.86×10^-2^ | 9.57×10^-3^ | - |
|  |  |  | PB | 3.85 | n.s. | 4.29×10^-1^ | 4.87×10^-2^ | - | n.s. | n.s. | - |
| Lobule ⅧA | L | Linear | ES | 5.05 | n.s. | 6.97×10^-1^ | 4.65×10^-2^ | - | n.s. | n.s. | - |
|  |  |  | CP | 4.98 | n.s. | 6.97×10^-1^ | 5.01×10^-2^ | - | n.s. | n.s. | - |
|  |  |  | HI | 5.08 | n.s. | 7.01×10^-1^ | 4.35×10^-2^ | - | n.s. | n.s. | - |
|  |  |  | PP | 5.02 | n.s. | 7.00×10^-1^ | 4.85×10^-2^ | - | n.s. | n.s. | - |
|  |  |  | PB | 5.20 | n.s. | 6.96×10^-1^ | n.s. | - | n.s. | n.s. | - |
|  | R | Linear | ES | 4.84 | n.s. | 7.11×10^-1^ | 4.78×10^-2^ | - | n.s. | n.s. | - |
|  |  |  | CP | 4.79 | n.s. | 7.08×10^-1^ | 4.89×10^-2^ | - | n.s. | n.s. | - |
|  |  |  | HI | 5.06 | n.s. | 7.11×10^-1^ | n.s. | - | n.s. | n.s. | - |
|  |  |  | PP | 4.96 | n.s. | 7.12×10^-1^ | 3.57×10^-2^ | - | n.s. | n.s. | - |
|  |  |  | PB | 4.83 | n.s. | 7.10×10^-1^ | n.s. | - | n.s. | n.s. | - |
| Lobule ⅧB | L | Linear | ES | 3.06 | n.s. | 5.37×10^-1^ | 4.53×10^-2^ | - | n.s. | n.s. | - |
|  |  |  | CP | 3.11 | n.s. | 5.37×10^-1^ | 3.96×10^-2^ | - | n.s. | n.s. | - |
|  |  |  | HI | 3.11 | n.s. | 5.35×10^-1^ | 3.73×10^-2^ | - | n.s. | n.s. | - |
|  |  |  | PP | 3.14 | n.s. | 5.38×10^-1^ | 3.67×10^-2^ | - | n.s. | n.s. | - |
|  |  |  | PB | 2.91 | n.s. | 5.38×10^-1^ | 5.90×10^-2^ | - | n.s. | n.s. | - |
|  | R | Linear | ES | 3.10 | n.s. | 4.85×10^-1^ | 3.49×10^-2^ | - | n.s. | n.s. | - |
|  |  |  | CP | 3.13 | n.s. | 4.86×10^-1^ | 3.13×10^-2^ | - | n.s. | n.s. | - |
|  |  |  | HI | 3.21 | n.s. | 4.86×10^-1^ | 2.34×10^-2^ | - | n.s. | n.s. | - |
|  |  |  | PP | 3.18 | n.s. | 4.85×10^-1^ | 2.52×10^-2^ | - | n.s. | n.s. | - |
|  |  |  | PB | 3.08 | n.s. | 4.86×10^-1^ | n.s. | - | n.s. | n.s. | - |
| Lobule Ⅸ | L | Linear | ES | 2.99 | n.s. | 2.91×10^-1^ | 1.46×10^-2^ | - | n.s. | n.s. | - |
|  |  |  | CP | 2.94 | n.s. | 2.92×10^-1^ | 1.82×10^-2^ | - | n.s. | n.s. | - |
|  |  |  | HI | 2.99 | n.s. | 2.90×10^-1^ | n.s. | - | n.s. | n.s. | - |
|  |  |  | PP | 3.03 | n.s. | 2.93×10^-1^ | n.s. | - | n.s. | n.s. | - |
|  |  |  | PB | 2.90 | n.s. | 2.91×10^-1^ | n.s. | - | n.s. | n.s. | - |
|  | R | Linear | ES | 3.14 | n.s. | 3.27×10^-1^ | 1.78×10^-2^ | - | n.s. | n.s. | - |
|  |  |  | CP | 3.17 | n.s. | 3.27×10^-1^ | 1.41×10^-2^ | - | n.s. | n.s. | - |
|  |  |  | HI | 3.20 | n.s. | 3.26×10^-1^ | n.s. | - | n.s. | n.s. | - |
|  |  |  | PP | 3.21 | n.s. | 3.26×10^-1^ | n.s. | - | n.s. | n.s. | - |
|  |  |  | PB | 3.00 | n.s. | 3.27×10^-1^ | 3.24×10^-2^ | - | n.s. | n.s. | - |
| Lobule Ⅹ | L | Linear | ES | 4.74×10^-1^ | n.s. | 4.13×10^-2^ | 8.26×10^-3^ | - | n.s. | n.s. | - |
|  |  |  | CP | 4.83×10^-1^ | n.s. | 4.13×10^-2^ | 7.13×10^-3^ | - | n.s. | n.s. | - |
|  |  |  | HI | 4.74×10^-1^ | n.s. | 4.11×10^-2^ | 7.97×10^-3^ | - | n.s. | n.s. | - |
|  |  |  | PP | 4.93×10^-1^ | n.s. | 4.18×10^-2^ | 6.57×10^-3^ | - | n.s. | n.s. | - |
|  |  |  | PB | 4.53×10^-1^ | n.s. | 4.11×10^-2^ | 1.08×10^-2^ | - | n.s. | n.s. | - |
|  | R | Linear | ES | 4.78×10^-1^ | n.s. | 3.98×10^-2^ | 6.62×10^-3^ | - | n.s. | n.s. | - |
|  |  |  | CP | 4.68×10^-1^ | n.s. | 3.96×10^-2^ | 7.50×10^-3^ | - | n.s. | n.s. | - |
|  |  |  | HI | 4.84×10^-1^ | n.s. | 3.95×10^-2^ | 5.93×10^-3^ | - | n.s. | n.s. | - |
|  |  |  | PP | 4.97×10^-1^ | n.s. | 4.01×10^-2^ | 4.90×10^-3^ | - | n.s. | n.s. | - |
|  |  |  | PB | 4.57×10^-1^ | n.s. | 3.97×10^-2^ | 9.11×10^-3^ | - | n.s. | n.s. | - |

The level of significance is 0.0066 after multiple comparisons correction, – = not applicable; n.s. = non-significant, HS = Hemisphere, L = left, R = right, EBP = emotional and behavioural problems, ES = Emotional Symptoms, CP =Conduct Problems , HI = Hyperactivity/Inattention, PP = Peer Relationship Problems, PB = Prosocial Behaviors

Table S2 Model parameters for fixed effects when including level of emotional and behavioural problems in the best fitting models for whole cerebellum and cerebeller subregion cortical thicknesses.

| Cortical thickness | HS | Best fitting model | EBP Scales | Intercept | Site | Gender coefficient  β1 | Age coefficient  β2 | Age^2^ coefficient  β3 | EBP | Age×EBP coefficient  β5 | Age^2^×EBP coefficient  β6 |
| --- | --- | --- | --- | --- | --- | --- | --- | --- | --- | --- | --- |
| Cerebellum | L | Linear | ES | 4.76 | n.s. | 8.84×10^-2^ | 1.52×10^-2^ | - | n.s. | n.s. | - |
|  |  |  | CP | 4.77 | n.s. | 8.85×10^-2^ | 1.47×10^-2^ | - | n.s. | n.s. | - |
|  |  |  | HI | 4.75 | n.s. | 8.83×10^-2^ | 1.57×10^-2^ | - | n.s. | n.s. | - |
|  |  |  | PP | 4.77 | n.s. | 8.82×10^-2^ | 1.36×10^-2^ | - | n.s. | n.s. | - |
|  |  |  | PB | 4.72 | n.s. | 8.90×10^-2^ | 1.85×10^-2^ | - | n.s. | n.s. | - |
|  | R | Linear | ES | 4.81 | n.s. | 8.27×10^-2^ | 8.93×10^-3^ | - | n.s. | n.s. | - |
|  |  |  | CP | 4.80 | n.s. | 8.27×10^-2^ | 9.39×10^-3^ | - | n.s. | n.s. | - |
|  |  |  | HI | 4.81 | n.s. | 8.23×10^-2^ | 8.29×10^-3^ | - | n.s. | n.s. | - |
|  |  |  | PP | 4.83 | n.s. | 8.25×10^-2^ | 7.17×10^-3^ | - | n.s. | n.s. | - |
|  |  |  | PB | 4.70 | n.s. | 8.34×10^-2^ | 1.93×10^-2^ | - | n.s. | n.s. | - |
| Lobule Ⅰ-Ⅱ | L | None | ES | 2.32 | n.s. | 1.61×10^-1^ | n.s. | - | n.s. | - | - |
|  |  |  | CP | 2.28 | n.s. | 1.61×10^-1^ | n.s. | - | n.s. | - | - |
|  |  |  | HI | 2.30 | n.s. | 1.62×10^-1^ | n.s. | - | n.s. | - | - |
|  |  |  | PP | 2.30 | n.s. | 1.61×10^-1^ | n.s. | - | n.s. | - | - |
|  |  |  | PB | 2.31 | n.s. | 1.62×10^-1^ | n.s. | - | n.s. | - | - |
|  | R | None | ES | 2.22 | 7.35×10^-2^ | 1.69×10^-1^ | n.s. | - | n.s. | - | - |
|  |  |  | CP | 2.20 | 7.31×10^-2^ | 1.70×10^-1^ | n.s. | - | n.s. | - | - |
|  |  |  | HI | 2.20 | 7.32×10^-2^ | 1.70×10^-1^ | n.s. | - | n.s. | - | - |
|  |  |  | PP | 2.21 | 7.33×10^-2^ | 1.71×10^-1^ | n.s. | - | n.s. | - | - |
|  |  |  | PB | 2.19 | 7.32×10^-2^ | 1.71×10^-1^ | n.s. | - | n.s. | - | - |
| Lobule Ⅲ | L | None | ES | 3.71 | n.s. | 1.20×10^-1^ | n.s. | - | n.s. | - | - |
|  |  |  | CP | 3.69 | n.s. | 1.20×10^-1^ | n.s. | - | n.s. | - | - |
|  |  |  | HI | 3.73 | n.s. | 1.23×10^-1^ | n.s. | - | n.s. | - | - |
|  |  |  | PP | 3.69 | n.s. | 1.19×10^-1^ | n.s. | - | n.s. | - | - |
|  |  |  | PB | 3.72 | n.s. | 1.20×10^-1^ | n.s. | - | n.s. | - | - |
|  | R | None | ES | 3.53 | n.s. | 1.36×10^-1^ | n.s. | - | n.s. | - | - |
|  |  |  | CP | 3.52 | n.s. | 1.36×10^-1^ | n.s. | - | n.s. | - | - |
|  |  |  | HI | 3.54 | n.s. | 1.37×10^-1^ | n.s. | - | n.s. | - | - |
|  |  |  | PP | 3.53 | n.s. | 1.36×10^-1^ | n.s. | - | n.s. | - | - |
|  |  |  | PB | 3.56 | n.s. | 1.35×10^-1^ | n.s. | - | n.s. | - | - |
| Lobule Ⅳ | L | Linear | ES | 5.13 | n.s. | 5.93×10^-2^ | 6.77×10^-3^ | - | n.s. | n.s. | - |
|  |  |  | CP | 5.13 | n.s. | 5.90×10^-2^ | 7.21×10^-3^ | - | n.s. | n.s. | - |
|  |  |  | HI | 5.13 | n.s. | 5.90×10^-2^ | n.s. | - | n.s. | n.s. | - |
|  |  |  | PP | 5.17 | n.s. | 5.89×10^-2^ | n.s. | - | n.s. | n.s. | - |
|  |  |  | PB | 5.07 | n.s. | 5.90×10^-2^ | n.s. | - | n.s. | n.s. | - |
|  | R | Linear | ES | 5.07 | n.s. | 5.79×10^-2^ | n.s. | - | n.s. | n.s. | - |
|  |  |  | CP | 5.08 | n.s. | 5.76×10^-2^ | n.s. | - | n.s. | n.s. | - |
|  |  |  | HI | 5.06 | n.s. | 5.68×10^-2^ | n.s. | - | n.s. | n.s. | - |
|  |  |  | PP | 5.11 | n.s. | 5.76×10^-2^ | n.s. | - | -2.76×10^-2^ | 2.84×10^-3^ | - |
|  |  |  | PB | 4.95 | n.s. | 5.82×10^-2^ | 1.73×10^-2^ | - | n.s. | n.s. | - |
| Lobule Ⅴ | L | Linear | ES | 5.11 | n.s. | 6.62×10^-2^ | 9.16×10^-3^ | - | n.s. | n.s. | - |
|  |  |  | CP | 5.11 | n.s. | 6.65×10^-2^ | 9.41×10^-3^ | - | n.s. | n.s. | - |
|  |  |  | HI | 5.11 | n.s. | 6.68×10^-2^ | 9.46×10^-3^ | - | n.s. | n.s. | - |
|  |  |  | PP | 5.12 | n.s. | 6.62×10^-2^ | 8.20×10^-3^ | - | n.s. | n.s. | - |
|  |  |  | PB | 4.99 | n.s. | 6.83×10^-2^ | 1.86×10^-2^ | - | n.s. | n.s. | - |
|  | R | Linear | ES | 4.93 | n.s. | 9.09×10^-2^ | 1.17×10^-2^ | - | n.s. | n.s. | - |
|  |  |  | CP | 4.93 | n.s. | 9.07×10^-2^ | 1.19×10^-2^ | - | n.s. | n.s. | - |
|  |  |  | HI | 4.90 | n.s. | 9.02×10^-2^ | 1.44×10^-2^ | - | n.s. | n.s. | - |
|  |  |  | PP | 4.93 | n.s. | 9.06×10^-2^ | 1.19×10^-2^ | - | n.s. | n.s. | - |
|  |  |  | PB | 4.76 | n.s. | 9.21×10^-2^ | 2.72×10^-2^ | - | n.s. | n.s. | - |
| Lobule Ⅵ | L | Linear | ES | 5.14 | n.s. | 6.49×10^-2^ | 7.43×10^-3^ | - | n.s. | n.s. | - |
|  |  |  | CP | 5.15 | n.s. | 6.47×10^-2^ | n.s. | - | n.s. | n.s. | - |
|  |  |  | HI | 5.15 | n.s. | 6.45×10^-2^ | n.s. | - | n.s. | n.s. | - |
|  |  |  | PP | 5.16 | n.s. | 6.43×10^-2^ | n.s. | - | n.s. | n.s. | - |
|  |  |  | PB | 5.09 | n.s. | 6.53×10^-2^ | n.s. | - | n.s. | n.s. | - |
|  | R | Linear | ES | 5.10 | n.s. | 5.51×10^-2^ | n.s. | - | n.s. | n.s. | - |
|  |  |  | CP | 5.13 | n.s. | 5.49×10^-2^ | n.s. | - | n.s. | n.s. | - |
|  |  |  | HI | 5.12 | n.s. | 5.46×10^-2^ | n.s. | - | n.s. | n.s. | - |
|  |  |  | PP | 5.12 | n.s. | 5.47×10^-2^ | n.s. | - | n.s. | n.s. | - |
|  |  |  | PB | 5.05 | n.s. | 5.54×10^-2^ | n.s. | - | n.s. | n.s. | - |
| Lobule  Crus I | L | Linear | ES | 4.57 | n.s. | 1.21×10^-1^ | 2.23×10^-2^ | - | n.s. | n.s. | - |
|  |  |  | CP | 4.58 | n.s. | 1.21×10^-1^ | 2.14×10^-2^ | - | n.s. | n.s. | - |
|  |  |  | HI | 4.55 | n.s. | 1.21×10^-1^ | 2.44×10^-2^ | - | n.s. | n.s. | - |
|  |  |  | PP | 4.55 | n.s. | 1.21×10^-1^ | 2.44×10^-2^ | - | n.s. | n.s. | - |
|  |  |  | PB | 4.54 | n.s. | 1.21×10^-1^ | 2.43×10^-2^ | - | n.s. | n.s. | - |
|  | R | Linear | ES | 4.73 | n.s. | 1.08×10^-1^ | 1.12×10^-2^ | - | n.s. | n.s. | - |
|  |  |  | CP | 4.70 | n.s. | 1.08×10^-1^ | 1.35×10^-2^ | - | n.s. | n.s. | - |
|  |  |  | HI | 4.73 | n.s. | 1.07×10^-1^ | n.s. | - | n.s. | n.s. | - |
|  |  |  | PP | 4.74 | n.s. | 1.08×10^-1^ | 1.01×10^-2^ | - | n.s. | n.s. | - |
|  |  |  | PB | 4.62 | n.s. | 1.09×10^-1^ | n.s. | - | n.s. | n.s. | - |
| Lobule  Crus II | L | Linear | ES | 4.48 | n.s. | 9.28×10^-2^ | 3.09×10^-2^ | - | n.s. | n.s. | - |
|  |  |  | CP | 4.50 | n.s. | 9.35×10^-2^ | 2.89×10^-2^ | - | n.s. | n.s. | - |
|  |  |  | HI | 4.49 | n.s. | 9.32×10^-2^ | 2.94×10^-2^ | - | n.s. | n.s. | - |
|  |  |  | PP | 4.49 | n.s. | 9.21×10^-2^ | 2.93×10^-2^ | - | n.s. | n.s. | - |
|  |  |  | PB | 4.50 | n.s. | 9.37×10^-2^ | n.s. | - | n.s. | n.s. | - |
|  | R | Linear | ES | 4.75 | n.s. | 6.02×10^-2^ | 1.79×10^-2^ | - | n.s. | n.s. | - |
|  |  |  | CP | 4.74 | n.s. | 6.14×10^-2^ | 1.90×10^-2^ | - | n.s. | n.s. | - |
|  |  |  | HI | 4.74 | n.s. | 6.05×10^-2^ | 1.82×10^-2^ | - | n.s. | n.s. | - |
|  |  |  | PP | 4.75 | n.s. | 6.12×10^-2^ | 1.75×10^-2^ | - | n.s. | n.s. | - |
|  |  |  | PB | 4.59 | n.s. | 6.24×10^-2^ | 3.14×10^-2^ | - | n.s. | n.s. | - |
| Lobule ⅦB | L | Quadratic | ES | 5.13 | n.s. | 8.32×10^-2^ | n.s. | n.s. | n.s. | n.s. | n.s. |
|  |  |  | CP | 4.93 | n.s. | 8.41×10^-2^ | n.s. | n.s. | n.s. | n.s. | n.s. |
|  |  |  | HI | 5.27 | n.s. | 8.30×10^-2^ | n.s. | n.s. | n.s. | n.s. | n.s. |
|  |  |  | PP | 5.00 | n.s. | 8.41×10^-2^ | n.s. | n.s. | n.s. | n.s. | n.s. |
|  |  |  | PB | 5.81 | n.s. | 8.59×10^-2^ | -1.82×10^-1^ | 9.42×10^-3^ | n.s. | n.s. | n.s. |
|  | R | Linear | ES | 4.91 | n.s. | 9.17×10^-2^ | 8.10×10^-3^ | - | n.s. | n.s. | - |
|  |  |  | CP | 4.92 | n.s. | 9.19×10^-2^ | n.s. | - | n.s. | n.s. | - |
|  |  |  | HI | 4.92 | n.s. | 9.23×10^-2^ | n.s. | - | n.s. | n.s. | - |
|  |  |  | PP | 4.91 | n.s. | 9.05×10^-2^ | n.s. | - | n.s. | n.s. | - |
|  |  |  | PB | 4.77 | n.s. | 9.39×10^-2^ | n.s. | - | n.s. | n.s. | - |
| Lobule ⅧA | L | Linear | ES | 4.85 | n.s. | 6.96×10^-2^ | 9.56×10^-3^ | - | n.s. | n.s. | - |
|  |  |  | CP | 4.87 | n.s. | 6.91×10^-2^ | n.s. | - | n.s. | n.s. | - |
|  |  |  | HI | 4.83 | n.s. | 6.85×10^-2^ | 1.15×10^-2^ | - | n.s. | n.s. | - |
|  |  |  | PP | 4.87 | n.s. | 6.89×10^-2^ | n.s. | - | n.s. | n.s. | - |
|  |  |  | PB | 4.84 | n.s. | 6.97×10^-2^ | n.s. | - | n.s. | n.s. | - |
|  | R | Linear | ES | 4.75 | n.s. | 8.67×10^-2^ | 9.12×10^-3^ | - | n.s. | n.s. | - |
|  |  |  | CP | 4.74 | n.s. | 8.58×10^-2^ | n.s. | - | n.s. | n.s. | - |
|  |  |  | HI | 4.77 | n.s. | 8.58×10^-2^ | n.s. | - | n.s. | n.s. | - |
|  |  |  | PP | 4.75 | n.s. | 8.55×10^-2^ | n.s. | - | n.s. | n.s. | - |
|  |  |  | PB | 4.74 | n.s. | 8.55×10^-2^ | n.s. | - | n.s. | n.s. | - |
| Lobule ⅧB | L | Linear | ES | 4.78 | 2.75×10^-2^ | 1.15×10^-1^ | 1.95×10^-2^ | - | n.s. | n.s. | - |
|  |  |  | CP | 4.77 | 2.72×10^-2^ | 1.14×10^-1^ | 2.00×10^-2^ | - | n.s. | n.s. | - |
|  |  |  | HI | 4.76 | 2.73×10^-2^ | 1.14×10^-1^ | 2.09×10^-2^ | - | n.s. | n.s. | - |
|  |  |  | PP | 4.84 | 2.76×10^-2^ | 1.15×10^-1^ | 1.37×10^-2^ | - | n.s. | n.s. | - |
|  |  |  | PB | 4.74 | 2.77×10^-2^ | 1.15×10^-1^ | n.s. | - | n.s. | n.s. | - |
|  | R | None | ES | 4.84 | n.s. | 1.16×10^-1^ | n.s. | - | n.s. | - | - |
|  |  |  | CP | 4.83 | n.s. | 1.16×10^-1^ | n.s. | - | n.s. | - | - |
|  |  |  | HI | 4.84 | n.s. | 1.16×10^-1^ | n.s. | - | n.s. | - | - |
|  |  |  | PP | 4.84 | n.s. | 1.15×10^-1^ | n.s. | - | n.s. | - | - |
|  |  |  | PB | 4.87 | n.s. | 1.15×10^-1^ | n.s. | - | n.s. | - | - |
| Lobule Ⅸ | L | None | ES | 4.56 | n.s. | 9.82×10^-2^ | n.s. | - | n.s. | - | - |
|  |  |  | CP | 4.55 | n.s. | 9.86×10^-2^ | n.s. | - | n.s. | - | - |
|  |  |  | HI | 4.53 | n.s. | 9.68×10^-2^ | n.s. | - | n.s. | - | - |
|  |  |  | PP | 4.56 | n.s. | 1.00×10^-1^ | n.s. | - | n.s. | - | - |
|  |  |  | PB | 4.56 | n.s. | 9.82×10^-2^ | n.s. | - | n.s. | - | - |
|  | R | None | ES | 4.71 | n.s. | 9.91×10^-2^ | n.s. | - | n.s. | - | - |
|  |  |  | CP | 4.71 | n.s. | 9.91×10^-2^ | n.s. | - | n.s. | - | - |
|  |  |  | HI | 4.69 | n.s. | 9.81×10^-2^ | n.s. | - | n.s. | - | - |
|  |  |  | PP | 4.71 | n.s. | 9.98×10^-2^ | n.s. | - | n.s. | - | - |
|  |  |  | PB | 4.74 | n.s. | 9.73×10^-2^ | n.s. | - | n.s. | - | - |
| Lobule Ⅹ | L | Linear | ES | 3.34 | n.s. | 1.47×10^-1^ | 3.77×10^-2^ | - | n.s. | n.s. | - |
|  |  |  | CP | 3.36 | n.s. | 1.46×10^-1^ | 3.62×10^-2^ | - | n.s. | n.s. | - |
|  |  |  | HI | 3.35 | n.s. | 1.48×10^-1^ | 3.77×10^-2^ | - | n.s. | n.s. | - |
|  |  |  | PP | 3.44 | n.s. | 1.48×10^-1^ | 2.86×10^-2^ | - | n.s. | n.s. | - |
|  |  |  | PB | 3.20 | n.s. | 1.47×10^-1^ | 5.30×10^-2^ | - | n.s. | n.s. | - |
|  | R | Linear | ES | 2.77 | n.s. | 1.80×10^-1^ | 3.26×10^-2^ | - | n.s. | n.s. | - |
|  |  |  | CP | 2.64 | n.s. | 1.79×10^-1^ | 4.61×10^-2^ | - | n.s. | n.s. | - |
|  |  |  | HI | 2.80 | n.s. | 1.80×10^-1^ | n.s. | - | n.s. | n.s. | - |
|  |  |  | PP | 2.95 | n.s. | 1.80×10^-1^ | n.s. | - | -1.11×10^-1^ | 1.09×10^-2^ | - |
|  |  |  | PB | 2.71 | n.s. | 1.77×10^-1^ | n.s. | - | n.s. | n.s. | - |

The level of significance is 0.0065 after multiple comparisons correction, – = not applicable; n.s. = non-significant, HS = Hemisphere, L = left, R = right, EBP = emotional and behavioural problems, ES = Emotional Symptoms, CP =Conduct Problems , HI = Hyperactivity/Inattention, PP = Peer Relationship Problems, PB = Prosocial Behaviors

Table S3 Model parameters for fixed effects when including level of emotional and behavioural problems in the best fitting models for whole cerebellum and cerebeller subregion surface areas.

| Surface area | HS | Best fitting model | EBP Scales | Intercept | Site | Gender coefficient  β1 | Age coefficient  β2 | Age^2^ coefficient  β3 | EBP | Age×EBP coefficient  β5 | Age^2^×EBP coefficient  β6 |
| --- | --- | --- | --- | --- | --- | --- | --- | --- | --- | --- | --- |
| Cerebellum | L | Quadratic | ES | 9.01 | n.s. | 6.77×10^-1^ | 2.25×10^-1^ | -9.48×10^-3^ | n.s. | n.s. | n.s. |
|  |  |  | CP | 9.30 | n.s. | 6.78×10^-1^ | 1.69×10^-1^ | -6.93×10^-3^ | n.s. | n.s. | n.s. |
|  |  |  | HI | 9.32 | n.s. | 6.77×10^-1^ | 1.72×10^-1^ | -7.53×10^-3^ | n.s. | n.s. | n.s. |
|  |  |  | PP | 9.08 | n.s. | 6.80×10^-1^ | 2.13×10^-1^ | -8.99×10^-3^ | n.s. | n.s. | n.s. |
|  |  |  | PB | 8.22 | n.s. | 6.75×10^-1^ | 3.84×10^-1^ | -1.73×10^-2^ | n.s. | n.s. | n.s. |
|  | R | Quadratic | ES | 8.63 | n.s. | 7.74×10^-1^ | 2.61×10^-1^ | -1.02×10^-2^ | 3.07×10^-1^ | -5.79×10^-2^ | n.s. |
|  |  |  | CP | 9.26 | n.s. | 7.61×10^-1^ | 1.46×10^-1^ | n.s. | n.s. | n.s. | n.s. |
|  |  |  | HI | 9.16 | n.s. | 7.62×10^-1^ | 1.71×10^-1^ | -6.41×10^-3^ | n.s. | n.s. | n.s. |
|  |  |  | PP | 8.99 | n.s. | 7.63×10^-1^ | 2.01×10^-1^ | -7.77×10^-3^ | n.s. | n.s. | n.s. |
|  |  |  | PB | 8.56 | n.s. | 7.60×10^-1^ | 2.85×10^-1^ | n.s. | n.s. | n.s. | n.s. |
| Lobule Ⅰ-Ⅱ | L | Linear | ES | 1.14×10^-2^ | n.s. | n.s. | n.s. | - | n.s. | n.s. | - |
|  |  |  | CP | 1.13×10^-2^ | n.s. | n.s. | n.s. | - | n.s. | n.s. | - |
|  |  |  | HI | 1.16×10^-2^ | n.s. | n.s. | n.s. | - | n.s. | n.s. | - |
|  |  |  | PP | 1.10×10^-2^ | n.s. | n.s. | n.s. | - | n.s. | n.s. | - |
|  |  |  | PB | 1.07×10^-2^ | n.s. | n.s. | n.s. | - | n.s. | n.s. | - |
|  | R | None | ES | 1.70×10^-2^ | n.s. | n.s. | n.s. | - | n.s. | - | - |
|  |  |  | CP | 1.72×10^-2^ | n.s. | n.s. | n.s. | - | n.s. | - | - |
|  |  |  | HI | 1.73×10^-2^ | n.s. | n.s. | n.s. | - | n.s. | - | - |
|  |  |  | PP | 1.73×10^-2^ | n.s. | n.s. | n.s. | - | n.s. | - | - |
|  |  |  | PB | 1.75×10^-2^ | n.s. | n.s. | n.s. | - | n.s. | - | - |
| Lobule Ⅲ | L | Linear | ES | 1.34×10^-1^ | n.s. | 8.35×10^-3^ | 9.88×10^-4^ | - | n.s. | n.s. | - |
|  |  |  | CP | 1.37×10^-1^ | n.s. | 8.28×10^-3^ | n.s. | - | n.s. | n.s. | - |
|  |  |  | HI | 1.34×10^-1^ | n.s. | 8.25×10^-3^ | 9.40×10^-4^ | - | n.s. | n.s. | - |
|  |  |  | PP | 1.35×10^-1^ | n.s. | 8.31×10^-3^ | 8.86×10^-4^ | - | n.s. | n.s. | - |
|  |  |  | PB | 1.40×10^-1^ | n.s. | 8.34×10^-3^ | n.s. | - | n.s. | n.s. | - |
|  | R | Linear | ES | 1.38×10^-1^ | n.s. | 9.27×10^-3^ | 8.10×10^-4^ | - | n.s. | n.s. | - |
|  |  |  | CP | 1.38×10^-1^ | n.s. | 9.20×10^-3^ | n.s. | - | n.s. | n.s. | - |
|  |  |  | HI | 1.38×10^-1^ | n.s. | 9.25×10^-3^ | n.s. | - | n.s. | n.s. | - |
|  |  |  | PP | 1.37×10^-1^ | n.s. | 9.32×10^-3^ | 9.76×10^-4^ | - | n.s. | n.s. | - |
|  |  |  | PB | 1.41×10^-1^ | n.s. | 9.22×10^-3^ | n.s. | - | n.s. | n.s. | - |
| Lobule Ⅳ | L | Quadratic | ES | 3.71×10^-1^ | -1.14×10^-2^ | 3.57×10^-2^ | n.s. | n.s. | n.s. | n.s. | n.s. |
|  |  |  | CP | 3.94×10^-1^ | -1.14×10^-2^ | 3.58×10^-2^ | n.s. | n.s. | n.s. | n.s. | n.s. |
|  |  |  | HI | 3.56×10^-1^ | -1.15×10^-2^ | 3.57×10^-2^ | n.s. | n.s. | n.s. | n.s. | n.s. |
|  |  |  | PP | 3.78×10^-1^ | -1.15×10^-2^ | 3.58×10^-2^ | n.s. | n.s. | n.s. | n.s. | n.s. |
|  |  |  | PB | 3.66×10^-1^ | -1.15×10^-2^ | 3.59×10^-2^ | n.s. | n.s. | n.s. | n.s. | n.s. |
|  | R | Quadratic | ES | 3.54×10^-1^ | -1.05×10^-2^ | 4.15×10^-2^ | 1.17×10^-2^ | -4.56×10^-4^ | n.s. | n.s. | n.s. |
|  |  |  | CP | 3.48×10^-1^ | -1.05×10^-2^ | 4.16×10^-2^ | 1.34×10^-2^ | -5.53×10^-4^ | n.s. | n.s. | n.s. |
|  |  |  | HI | 3.36×10^-1^ | -1.04×10^-2^ | 4.18×10^-2^ | 1.67×10^-2^ | -7.50×10^-4^ | n.s. | n.s. | n.s. |
|  |  |  | PP | 3.43×10^-1^ | -1.05×10^-2^ | 4.18×10^-2^ | 1.41×10^-2^ | -5.67×10^-4^ | n.s. | n.s. | n.s. |
|  |  |  | PB | 3.75×10^-1^ | -1.05×10^-2^ | 4.18×10^-2^ | n.s. | n.s. | n.s. | n.s. | n.s. |
| Lobule Ⅴ | L | Linear | ES | 6.66×10^-1^ | n.s. | 3.73×10^-2^ | 5.55×10^-3^ | - | n.s. | n.s. | - |
|  |  |  | CP | 6.68×10^-1^ | n.s. | 3.73×10^-2^ | 5.32×10^-3^ | - | n.s. | n.s. | - |
|  |  |  | HI | 6.80×10^-1^ | n.s. | 3.73×10^-2^ | 4.14×10^-3^ | - | n.s. | n.s. | - |
|  |  |  | PP | 6.71×10^-1^ | n.s. | 3.75×10^-2^ | 5.13×10^-3^ | - | n.s. | n.s. | - |
|  |  |  | PB | 6.63×10^-1^ | n.s. | 3.74×10^-2^ | 5.73×10^-3^ | - | n.s. | n.s. | - |
|  | R | Quadratic | ES | 5.86×10^-1^ | n.s. | 4.59×10^-2^ | 1.63×10^-2^ | n.s. | n.s. | n.s. | n.s. |
|  |  |  | CP | 5.68×10^-1^ | n.s. | 4.59×10^-2^ | 2.00×10^-2^ | n.s. | n.s. | n.s. | n.s. |
|  |  |  | HI | 5.81×10^-1^ | n.s. | 4.62×10^-2^ | 1.82×10^-2^ | n.s. | n.s. | n.s. | n.s. |
|  |  |  | PP | 5.91×10^-1^ | n.s. | 4.62×10^-2^ | n.s. | n.s. | n.s. | n.s. | n.s. |
|  |  |  | PB | 5.08×10^-1^ | n.s. | 4.63×10^-2^ | n.s. | n.s. | n.s. | n.s. | n.s. |
| Lobule Ⅵ | L | Quadratic | ES | 1.30 | n.s. | 7.00×10^-2^ | 5.23×10^-2^ | -2.17×10^-3^ | n.s. | n.s. | n.s. |
|  |  |  | CP | 1.35 | n.s. | 6.99×10^-2^ | 4.13×10^-2^ | -1.67×10^-3^ | n.s. | n.s. | n.s. |
|  |  |  | HI | 1.33 | n.s. | 6.97×10^-2^ | 4.67×10^-2^ | -1.94×10^-3^ | n.s. | n.s. | n.s. |
|  |  |  | PP | 1.29 | n.s. | 7.02×10^-2^ | 5.30×10^-2^ | -2.21×10^-3^ | n.s. | n.s. | n.s. |
|  |  |  | PB | 1.24 | n.s. | 6.99×10^-2^ | n.s. | n.s. | n.s. | n.s. | n.s. |
|  | R | Linear | ES | 1.44 | n.s. | 5.51×10^-2^ | 1.29×10^-2^ | - | n.s. | n.s. | - |
|  |  |  | CP | 1.45 | n.s. | 5.53×10^-2^ | 1.21×10^-2^ | - | n.s. | n.s. | - |
|  |  |  | HI | 1.45 | n.s. | 5.56×10^-2^ | 1.21×10^-2^ | - | n.s. | n.s. | - |
|  |  |  | PP | 1.44 | n.s. | 5.53×10^-2^ | 1.25×10^-2^ | - | n.s. | n.s. | - |
|  |  |  | PB | 1.43 | n.s. | 5.52×10^-2^ | 1.37×10^-2^ | - | n.s. | n.s. | - |
| Lobule  Crus I | L | Quadratic | ES | 2.01 | n.s. | 1.30×10^-1^ | 4.62×10^-2^ | -2.34×10^-3^ | n.s. | n.s. | n.s. |
|  |  |  | CP | 2.09 | n.s. | 1.30×10^-1^ | n.s. | n.s. | n.s. | n.s. | n.s. |
|  |  |  | HI | 2.12 | n.s. | 1.30×10^-1^ | n.s. | n.s. | n.s. | n.s. | n.s. |
|  |  |  | PP | 2.05 | n.s. | 1.31×10^-1^ | n.s. | n.s. | n.s. | n.s. | n.s. |
|  |  |  | PB | 1.92 | n.s. | 1.30×10^-1^ | n.s. | n.s. | n.s. | n.s. | n.s. |
|  | R | Linear | ES | 2.12 | n.s. | 1.60×10^-1^ | 7.99×10^-3^ | - | n.s. | n.s. | - |
|  |  |  | CP | 2.14 | n.s. | 1.60×10^-1^ | n.s. | - | n.s. | n.s. | - |
|  |  |  | HI | 2.12 | n.s. | 1.60×10^-1^ | 7.99×10^-3^ | - | n.s. | n.s. | - |
|  |  |  | PP | 2.13 | n.s. | 1.60×10^-1^ | 7.60×10^-3^ | - | n.s. | n.s. | - |
|  |  |  | PB | 2.17 | n.s. | 1.60×10^-1^ | n.s. | - | n.s. | n.s. | - |
| Lobule  Crus II | L | Quadratic | ES | 1.54 | n.s. | 6.45×10^-2^ | n.s. | n.s. | n.s. | n.s. | n.s. |
|  |  |  | CP | 1.53 | n.s. | 6.50×10^-2^ | n.s. | n.s. | n.s. | n.s. | n.s. |
|  |  |  | HI | 1.38 | n.s. | 6.51×10^-2^ | n.s. | -2.83×10^-3^ | n.s. | n.s. | n.s. |
|  |  |  | PP | 1.46 | n.s. | 6.51×10^-2^ | n.s. | n.s. | n.s. | n.s. | n.s. |
|  |  |  | PB | 1.20 | n.s. | 6.48×10^-2^ | n.s. | n.s. | n.s. | n.s. | n.s. |
|  | R | Quadratic | ES | 1.34 | n.s. | 1.08×10^-1^ | 5.81×10^-2^ | -2.90×10^-3^ | n.s. | n.s. | n.s. |
|  |  |  | CP | 1.41 | n.s. | 1.09×10^-1^ | n.s. | n.s. | n.s. | n.s. | n.s. |
|  |  |  | HI | 1.34 | n.s. | 1.09×10^-1^ | 5.85×10^-2^ | -2.99×10^-3^ | n.s. | n.s. | n.s. |
|  |  |  | PP | 1.36 | n.s. | 1.08×10^-1^ | 5.13×10^-2^ | -2.51×10^-3^ | n.s. | n.s. | n.s. |
|  |  |  | PB | 1.21 | n.s. | 1.08×10^-1^ | n.s. | n.s. | n.s. | n.s. | n.s. |
| Lobule ⅦB | L | None | ES | 8.41×10^-1^ | n.s. | 5.78×10^-2^ | n.s. | - | n.s. | - | - |
|  |  |  | CP | 8.34×10^-1^ | n.s. | 5.81×10^-2^ | n.s. | - | n.s. | - | - |
|  |  |  | HI | 8.43×10^-1^ | n.s. | 5.87×10^-2^ | n.s. | - | n.s. | - | - |
|  |  |  | PP | 8.35×10^-1^ | n.s. | 5.82×10^-2^ | n.s. | - | n.s. | - | - |
|  |  |  | PB | 8.42×10^-1^ | n.s. | 5.76×10^-2^ | n.s. | - | n.s. | - | - |
|  | R | Linear | ES | 8.41×10^-1^ | n.s. | 6.78×10^-2^ | 3.85×10^-3^ | - | n.s. | n.s. | - |
|  |  |  | CP | 8.55×10^-1^ | n.s. | 6.84×10^-2^ | n.s. | - | n.s. | n.s. | - |
|  |  |  | HI | 8.44×10^-1^ | n.s. | 6.88×10^-2^ | n.s. | - | n.s. | n.s. | - |
|  |  |  | PP | 8.70×10^-1^ | n.s. | 6.82×10^-2^ | n.s. | - | -1.61×10^-2^ | 1.59×10^-3^ | - |
|  |  |  | PB | 8.13×10^-1^ | n.s. | 6.84×10^-2^ | n.s. | - | n.s. | n.s. | - |
| Lobule ⅧA | L | Linear | ES | 1.05 | n.s. | 1.23×10^-1^ | 6.51×10^-3^ | - | n.s. | n.s. | - |
|  |  |  | CP | 1.03 | n.s. | 1.24×10^-1^ | 7.44×10^-3^ | - | n.s. | n.s. | - |
|  |  |  | HI | 1.06 | n.s. | 1.24×10^-1^ | n.s. | - | n.s. | n.s. | - |
|  |  |  | PP | 1.04 | n.s. | 1.24×10^-1^ | 7.42×10^-3^ | - | n.s. | n.s. | - |
|  |  |  | PB | 1.08 | n.s. | 1.23×10^-1^ | n.s. | - | n.s. | n.s. | - |
|  | R | Linear | ES | 1.02 | n.s. | 1.26×10^-1^ | 7.50×10^-3^ | - | n.s. | n.s. | - |
|  |  |  | CP | 1.02 | n.s. | 1.25×10^-1^ | 7.11×10^-3^ | - | n.s. | n.s. | - |
|  |  |  | HI | 1.07 | n.s. | 1.26×10^-1^ | n.s. | - | -1.20×10^-2^ | n.s. | - |
|  |  |  | PP | 1.06 | n.s. | 1.25×10^-1^ | n.s. | - | -2.04×10^-2^ | n.s. | - |
|  |  |  | PB | 1.03 | n.s. | 1.26×10^-1^ | n.s. | - | n.s. | n.s. | - |
| Lobule ⅧB | L | Linear | ES | 6.49×10^-1^ | n.s. | 8.93×10^-2^ | 5.45×10^-3^ | - | n.s. | n.s. | - |
|  |  |  | CP | 6.63×10^-1^ | n.s. | 8.96×10^-2^ | 4.17×10^-3^ | - | n.s. | n.s. | - |
|  |  |  | HI | 6.65×10^-1^ | n.s. | 8.91×10^-2^ | n.s. | - | n.s. | n.s. | - |
|  |  |  | PP | 6.57×10^-1^ | n.s. | 8.96×10^-2^ | 4.67×10^-3^ | - | n.s. | n.s. | - |
|  |  |  | PB | 6.30×10^-1^ | n.s. | 8.96×10^-2^ | n.s. | - | n.s. | n.s. | - |
|  | R | Linear | ES | 6.42×10^-1^ | n.s. | 8.13×10^-2^ | 6.48×10^-3^ | - | n.s. | -1.42×10^-3^ | - |
|  |  |  | CP | 6.57×10^-1^ | n.s. | 8.14×10^-2^ | 5.12×10^-3^ | - | n.s. | n.s. | - |
|  |  |  | HI | 6.66×10^-1^ | n.s. | 8.13×10^-2^ | 4.04×10^-3^ | - | n.s. | n.s. | - |
|  |  |  | PP | 6.58×10^-1^ | n.s. | 8.13×10^-2^ | 4.79×10^-3^ | - | n.s. | n.s. | - |
|  |  |  | PB | 6.55×10^-1^ | n.s. | 8.14×10^-2^ | n.s. | - | n.s. | n.s. | - |
| Lobule Ⅸ | L | Linear | ES | 6.65×10^-1^ | ^-1^.67×10^-2^ | 4.88×10^-2^ | 2.87×10^-3^ | - | n.s. | n.s. | - |
|  |  |  | CP | 6.66×10^-1^ | -1.66×10^-2^ | 4.93×10^-2^ | 2.90×10^-3^ | - | n.s. | n.s. | - |
|  |  |  | HI | 6.71×10^-1^ | -1.66×10^-2^ | 4.91×10^-2^ | n.s. | - | n.s. | n.s. | - |
|  |  |  | PP | 6.58×10^-1^ | -1.67×10^-2^ | 4.92×10^-2^ | 3.57×10^-3^ | - | n.s. | n.s. | - |
|  |  |  | PB | 6.63×10^-1^ | -1.67×10^-2^ | 4.91×10^-2^ | n.s. | - | n.s. | n.s. | - |
|  | R | Linear | ES | 6.80×10^-1^ | n.s. | 5.46×10^-2^ | 3.87×10^-3^ | - | n.s. | n.s. | - |
|  |  |  | CP | 6.85×10^-1^ | n.s. | 5.48×10^-2^ | 3.34×10^-3^ | - | n.s. | n.s. | - |
|  |  |  | HI | 6.92×10^-1^ | n.s. | 5.48×10^-2^ | 2.66×10^-3^ | - | n.s. | n.s. | - |
|  |  |  | PP | 6.77×10^-1^ | n.s. | 5.46×10^-2^ | 4.08×10^-3^ | - | n.s. | n.s. | - |
|  |  |  | PB | 6.78×10^-1^ | n.s. | 5.48×10^-2^ | n.s. | - | n.s. | n.s. | - |
| Lobule Ⅹ | L | None | ES | 1.46×10^-1^ | -3.38×10^-3^ | 4.72×10^-3^ | n.s. | - | n.s. | - | - |
|  |  |  | CP | 1.46×10^-1^ | -3.37×10^-3^ | 4.76×10^-3^ | n.s. | - | n.s. | - | - |
|  |  |  | HI | 1.45×10^-1^ | -3.39×10^-3^ | 4.66×10^-3^ | n.s. | - | n.s. | - | - |
|  |  |  | PP | 1.46×10^-1^ | -3.38×10^-3^ | 4.78×10^-3^ | n.s. | - | n.s. | - | - |
|  |  |  | PB | 1.46×10^-1^ | -3.38×10^-3^ | 4.71×10^-3^ | n.s. | - | n.s. | - | - |
|  | R | None | ES | 1.78×10^-1^ | -3.63×10^-3^ | n.s. | n.s. | - | n.s. | - | - |
|  |  |  | CP | 1.78×10^-1^ | -3.62×10^-3^ | n.s. | n.s. | - | n.s. | - | - |
|  |  |  | HI | 1.77×10^-1^ | -3.64×10^-3^ | n.s. | n.s. | - | n.s. | - | - |
|  |  |  | PP | 1.78×10^-1^ | -3.63×10^-3^ | n.s. | n.s. | - | n.s. | - | - |
|  |  |  | PB | 1.77×10^-1^ | -3.63×10^-3^ | n.s. | n.s. | - | n.s. | - | - |

The level of significance is 0.0056 after multiple comparisons correction, – = not applicable; n.s. = non-significant, HS = Hemisphere, L = left, R = right, EBP = emotional and behavioural problems, ES = Emotional Symptoms, CP =Conduct Problems , HI = Hyperactivity/Inattention, PP = Peer Relationship Problems, PB = Prosocial Behaviors

Table S4 Model parameters for fixed effects when including level of emotional and behavioural problems in the best fitting models for whole cerebellum and cerebeller subregion gray matter volumes in boys.

| Gray matter volume | HS | Best fitting model | EBP Scales | Intercept | Site | Age coefficient  β2 | Age^2^ coefficient  β3 | Age^3^ coefficient  β3 | EBP | Age×EBP coefficient  β5 | Age^2^×EBP coefficient  β6 | Age^3^×EBP coefficient  β6 |
| --- | --- | --- | --- | --- | --- | --- | --- | --- | --- | --- | --- | --- |
| Cerebellum | L | Linear | ES | 5.16×10 | n.s. | 3.68×10^-1^ | - | - | n.s. | n.s. | - | - |
|  |  |  | CP | 5.15×10 | n.s. | 3.65×10^-1^ | - | - | n.s. | n.s. | - | - |
|  |  |  | HI | 5.20×10 | n.s. | 3.00×10^-1^ | - | - | -2.17×10^-1^ | 2.61×10^-2^ | - | - |
|  |  |  | PP | 5.15×10 | n.s. | 3.68×10^-1^ | - | - | n.s. | n.s. | - | - |
|  |  |  | PB | 5.11×10 | n.s. | 4.20×10^-1^ | - | - | n.s. | n.s. | - | - |
|  | R | Linear | ES | 5.11×10 | n.s. | 4.04×10^-1^ | - | - | n.s. | n.s. | - | - |
|  |  |  | CP | 5.09×10 | n.s. | 4.04×10^-1^ | - | - | n.s. | n.s. | - | - |
|  |  |  | HI | 5.13×10 | n.s. | 3.57×10^-1^ | - | - | n.s. | n.s. | - | - |
|  |  |  | PP | 5.16×10 | n.s. | 3.42×10^-1^ | - | - | n.s. | n.s. | - | - |
|  |  |  | PB | 5.00×10 | n.s. | 5.14×10^-1^ | - | - | n.s. | n.s. | - | - |
| Lobule Ⅰ-Ⅱ | L | None | ES | 2.81×10^-2^ | n.s. | n.s. | - | - | n.s. | - | - | - |
|  |  |  | CP | 2.75×10^-2^ | n.s. | n.s. | - | - | n.s. | - | - | - |
|  |  |  | HI | 2.74×10^-2^ | n.s. | n.s. | - | - | n.s. | - | - | - |
|  |  |  | PP | 2.81×10^-2^ | n.s. | n.s. | - | - | n.s. | - | - | - |
|  |  |  | PB | 2.74×10^-2^ | n.s. | n.s. | - | - | n.s. | - | - | - |
|  | R | None | ES | 3.81×10^-2^ | n.s. | n.s. | - | - | n.s. | - | - | - |
|  |  |  | CP | 3.84×10^-2^ | n.s. | n.s. | - | - | n.s. | - | - | - |
|  |  |  | HI | 3.85×10^-2^ | n.s. | n.s. | - | - | n.s. | - | - | - |
|  |  |  | PP | 3.93×10^-2^ | n.s. | n.s. | - | - | n.s. | - | - | - |
|  |  |  | PB | 3.72×10^-2^ | n.s. | n.s. | - | - | n.s. | - | - | - |
| Lobule Ⅲ | L | Linear | ES | 5.47×10^-1^ | n.s. | n.s. | - | - | n.s. | n.s. | - | - |
|  |  |  | CP | 5.43×10^-1^ | n.s. | n.s. | - | - | n.s. | n.s. | - | - |
|  |  |  | HI | 5.60×10^-1^ | n.s. | n.s. | - | - | n.s. | n.s. | - | - |
|  |  |  | PP | 5.47×10^-1^ | n.s. | n.s. | - | - | n.s. | n.s. | - | - |
|  |  |  | PB | 5.68×10^-1^ | n.s. | n.s. | - | - | n.s. | n.s. | - | - |
|  | R | Linear | ES | 5.32×10^-1^ | n.s. | n.s. | - | - | n.s. | n.s. | - | - |
|  |  |  | CP | 5.31×10^-1^ | n.s. | n.s. | - | - | n.s. | n.s. | - | - |
|  |  |  | HI | 5.57×10^-1^ | n.s. | n.s. | - | - | n.s. | n.s. | - | - |
|  |  |  | PP | 5.34×10^-1^ | n.s. | n.s. | - | - | n.s. | n.s. | - | - |
|  |  |  | PB | 5.32×10^-1^ | n.s. | n.s. | - | - | n.s. | n.s. | - | - |
| Lobule Ⅳ | L | Linear | ES | 2.33 | -8.21×10^-2^ | 1.17×10^-2^ | - | - | n.s. | n.s. | - | - |
|  |  |  | CP | 2.31 | -8.25×10^-2^ | 1.34×10^-2^ | - | - | n.s. | n.s. | - | - |
|  |  |  | HI | 2.28 | -8.26×10^-2^ | 1.70×10^-2^ | - | - | n.s. | n.s. | - | - |
|  |  |  | PP | 2.35 | -8.22×10^-2^ | n.s. | - | - | n.s. | n.s. | - | - |
|  |  |  | PB | 2.38 | -8.20×10^-2^ | n.s. | - | - | n.s. | n.s. | - | - |
|  | R | Linear | ES | 2.26 | n.s. | 1.74×10^-2^ | - | - | n.s. | n.s. | - | - |
|  |  |  | CP | 2.30 | n.s. | 1.39×10^-2^ | - | - | n.s. | n.s. | - | - |
|  |  |  | HI | 2.33 | n.s. | n.s. | - | - | n.s. | n.s. | - | - |
|  |  |  | PP | 2.29 | n.s. | 1.42×10^-2^ | - | - | n.s. | n.s. | - | - |
|  |  |  | PB | 2.30 | n.s. | n.s. | - | - | n.s. | n.s. | - | - |
| Lobule Ⅴ | L | Linear | ES | 3.57 | n.s. | 4.31×10^-2^ | - | - | n.s. | n.s. | - | - |
|  |  |  | CP | 3.64 | n.s. | 3.56×10^-2^ | - | - | n.s. | n.s. | - | - |
|  |  |  | HI | 3.64 | n.s. | 3.59×10^-2^ | - | - | n.s. | n.s. | - | - |
|  |  |  | PP | 3.59 | n.s. | 4.00×10^-2^ | - | - | n.s. | n.s. | - | - |
|  |  |  | PB | 3.53 | n.s. | 4.60×10^-2^ | - | - | n.s. | n.s. | - | - |
|  | R | Linear | ES | 3.54 | n.s. | 2.60×10^-2^ | - | - | n.s. | n.s. | - | - |
|  |  |  | CP | 3.52 | n.s. | 2.76×10^-2^ | - | - | n.s. | n.s. | - | - |
|  |  |  | HI | 3.59 | n.s. | 2.15×10^-2^ | - | - | n.s. | n.s. | - | - |
|  |  |  | PP | 3.55 | n.s. | 2.48×10^-2^ | - | - | n.s. | n.s. | - | - |
|  |  |  | PB | 3.59 | n.s. | n.s. | - | - | n.s. | n.s. | - | - |
| Lobule Ⅵ | L | Linear | ES | 8.28 | n.s. | 6.37×10^-2^ | - | - | n.s. | n.s. | - | - |
|  |  |  | CP | 8.25 | n.s. | 6.56×10^-2^ | - | - | n.s. | n.s. | - | - |
|  |  |  | HI | 8.35 | n.s. | 5.26×10^-2^ | - | - | n.s. | n.s. | - | - |
|  |  |  | PP | 8.25 | n.s. | 6.40×10^-2^ | - | - | n.s. | n.s. | - | - |
|  |  |  | PB | 8.04 | n.s. | 8.77×10^-2^ | - | - | n.s. | n.s. | - | - |
|  | R | Cubic | ES | 5.04 | n.s. | n.s. | n.s. | n.s. | n.s. | n.s. | n.s. | n.s. |
|  |  |  | CP | 6.43 | n.s. | n.s. | n.s. | n.s. | n.s. | n.s. | n.s. | n.s. |
|  |  |  | HI | 6.56 | n.s. | n.s. | n.s. | n.s. | n.s. | n.s. | n.s. | n.s. |
|  |  |  | PP | 7.14 | n.s. | n.s. | n.s. | n.s. | n.s. | n.s. | n.s. | n.s. |
|  |  |  | PB | n.s. | n.s. | 3.27 | -3.12×10^-1^ | 1.00×10^-2^ | n.s. | n.s. | n.s. | n.s. |
| Lobule  Crus I | L | Linear | ES | 1.11×10 | n.s. | 6.12×10^-2^ | - | - | n.s. | n.s. | - | - |
|  |  |  | CP | 1.12×10 | n.s. | 5.32×10^-2^ | - | - | n.s. | n.s. | - | - |
|  |  |  | HI | 1.12×10 | n.s. | 4.56×10^-2^ | - | - | n.s. | n.s. | - | - |
|  |  |  | PP | 1.10×10 | n.s. | 7.64×10^-2^ | - | - | n.s. | n.s. | - | - |
|  |  |  | PB | 1.12×10 | n.s. | n.s. | - | - | n.s. | n.s. | - | - |
|  | R | Linear | ES | 1.10×10 | n.s. | 9.07×10^-2^ | - | - | n.s. | n.s. | - | - |
|  |  |  | CP | 1.09×10 | n.s. | 9.11×10^-2^ | - | - | n.s. | n.s. | - | - |
|  |  |  | HI | 1.08×10 | n.s. | 1.04×10^-1^ | - | - | n.s. | n.s. | - | - |
|  |  |  | PP | 1.10×10 | n.s. | 8.38×10^-2^ | - | - | n.s. | n.s. | - | - |
|  |  |  | PB | 1.09×10 | n.s. | 9.39×10^-2^ | - | - | n.s. | n.s. | - | - |
| Lobule  Crus II | L | Linear | ES | 7.94 | n.s. | 4.44×10^-2^ | - | - | n.s. | n.s. | - | - |
|  |  |  | CP | 7.82 | n.s. | 5.47×10^-2^ | - | - | n.s. | n.s. | - | - |
|  |  |  | HI | 7.91 | n.s. | n.s. | - | - | n.s. | n.s. | - | - |
|  |  |  | PP | 7.91 | n.s. | n.s. | - | - | n.s. | n.s. | - | - |
|  |  |  | PB | 8.39 | n.s. | n.s. | - | - | n.s. | n.s. | - | - |
|  | R | Linear | ES | 8.43 | n.s. | n.s. | - | - | n.s. | n.s. | - | - |
|  |  |  | CP | 8.34 | n.s. | n.s. | - | - | n.s. | n.s. | - | - |
|  |  |  | HI | 8.32 | n.s. | n.s. | - | - | n.s. | n.s. | - | - |
|  |  |  | PP | 8.43 | n.s. | n.s. | - | - | n.s. | n.s. | - | - |
|  |  |  | PB | 8.58 | n.s. | n.s. | - | - | n.s. | n.s. | - | - |
| Lobule ⅦB | L | Linear | ES | 4.31 | n.s. | 3.08×10^-2^ | - | - | n.s. | n.s. | - | - |
|  |  |  | CP | 4.32 | n.s. | n.s. | - | - | n.s. | n.s. | - | - |
|  |  |  | HI | 4.45 | n.s. | n.s. | - | - | n.s. | n.s. | - | - |
|  |  |  | PP | 4.31 | n.s. | n.s. | - | - | n.s. | n.s. | - | - |
|  |  |  | PB | 4.27 | n.s. | n.s. | - | - | n.s. | n.s. | - | - |
|  | R | Linear | ES | 4.43 | n.s. | 4.10×10^-2^ | - | - | n.s. | n.s. | - | - |
|  |  |  | CP | 4.48 | n.s. | 3.52×10^-2^ | - | - | n.s. | n.s. | - | - |
|  |  |  | HI | 4.43 | n.s. | 4.26×10^-2^ | - | - | n.s. | n.s. | - | - |
|  |  |  | PP | 4.60 | n.s. | n.s. | - | - | n.s. | n.s. | - | - |
|  |  |  | PB | 4.15 | n.s. | 6.74×10^-2^ | - | - | n.s. | n.s. | - | - |
| Lobule ⅧA | L | Linear | ES | 5.76 | n.s. | 4.92×10^-2^ | - | - | n.s. | n.s. | - | - |
|  |  |  | CP | 5.78 | n.s. | 4.55×10^-2^ | - | - | n.s. | n.s. | - | - |
|  |  |  | HI | 5.82 | n.s. | 4.38×10^-2^ | - | - | n.s. | n.s. | - | - |
|  |  |  | PP | 5.72 | n.s. | 5.40×10^-2^ | - | - | n.s. | n.s. | - | - |
|  |  |  | PB | 5.75 | n.s. | n.s. | - | - | n.s. | n.s. | - | - |
|  | R | Linear | ES | 5.55 | n.s. | 4.36×10^-2^ | - | - | n.s. | n.s. | - | - |
|  |  |  | CP | 5.45 | n.s. | 4.91×10^-2^ | - | - | n.s. | n.s. | - | - |
|  |  |  | HI | 5.84 | n.s. | n.s. | - | - | n.s. | 9.42×10^-3^ | - | - |
|  |  |  | PP | 5.68 | n.s. | n.s. | - | - | n.s. | n.s. | - | - |
|  |  |  | PB | 5.20 | n.s. | n.s. | - | - | n.s. | n.s. | - | - |
| Lobule ⅧB | L | Linear | ES | 3.65 | n.s. | 4.30×10^-2^ | - | - | n.s. | n.s. | - | - |
|  |  |  | CP | 3.68 | n.s. | 4.03×10^-2^ | - | - | n.s. | n.s. | - | - |
|  |  |  | HI | 3.72 | n.s. | 3.29×10^-2^ | - | - | n.s. | n.s. | - | - |
|  |  |  | PP | 3.74 | n.s. | 3.23×10^-2^ | - | - | n.s. | n.s. | - | - |
|  |  |  | PB | 3.12 | n.s. | 9.73×10^-2^ | - | - | n.s. | n.s. | - | - |
|  | R | Linear | ES | 3.55 | n.s. | 3.82×10^-2^ | - | - | n.s. | n.s. | - | - |
|  |  |  | CP | 3.59 | n.s. | 3.46×10^-2^ | - | - | n.s. | n.s. | - | - |
|  |  |  | HI | 3.64 | n.s. | 2.88×10^-2^ | - | - | n.s. | n.s. | - | - |
|  |  |  | PP | 3.57 | n.s. | 3.39×10^-2^ | - | - | n.s. | n.s. | - | - |
|  |  |  | PB | 3.39 | n.s. | 5.82×10^-2^ | - | - | n.s. | n.s. | - | - |
| Lobule Ⅸ | L | Linear | ES | 3.33 | n.s. | 1.61×10^-2^ | - | - | n.s. | n.s. | - | - |
|  |  |  | CP | 3.26 | n.s. | 2.16×10^-2^ | - | - | n.s. | n.s. | - | - |
|  |  |  | HI | 3.40 | n.s. | n.s. | - | - | n.s. | n.s. | - | - |
|  |  |  | PP | 3.42 | n.s. | n.s. | - | - | n.s. | n.s. | - | - |
|  |  |  | PB | 3.11 | n.s. | 3.70×10^-2^ | - | - | n.s. | n.s. | - | - |
|  | R | Linear | ES | 3.49 | n.s. | 2.24×10^-2^ | - | - | n.s. | n.s. | - | - |
|  |  |  | CP | 3.51 | n.s. | 1.93×10^-2^ | - | - | n.s. | n.s. | - | - |
|  |  |  | HI | 3.60 | n.s. | n.s. | - | - | n.s. | n.s. | - | - |
|  |  |  | PP | 3.60 | n.s. | n.s. | - | - | n.s. | n.s. | - | - |
|  |  |  | PB | 3.30 | n.s. | 4.34×10^-2^ | - | - | n.s. | n.s. | - | - |
| Lobule Ⅹ | L | Linear | ES | 5.10×10^-1^ | n.s. | 9.31×10^-3^ | - | - | n.s. | n.s. | - | - |
|  |  |  | CP | 5.18×10^-1^ | n.s. | 8.39×10^-3^ | - | - | n.s. | n.s. | - | - |
|  |  |  | HI | 5.22×10^-1^ | n.s. | 7.83×10^-3^ | - | - | n.s. | n.s. | - | - |
|  |  |  | PP | 5.35×10^-1^ | n.s. | 7.02×10^-3^ | - | - | n.s. | n.s. | - | - |
|  |  |  | PB | 5.21×10^-1^ | n.s. | 8.75×10^-3^ | - | - | n.s. | n.s. | - | - |
|  | R | Linear | ES | 5.12×10^-1^ | n.s. | 7.58×10^-3^ | - | - | n.s. | n.s. | - | - |
|  |  |  | CP | 5.03×10^-1^ | n.s. | 8.52×10^-3^ | - | - | n.s. | n.s. | - | - |
|  |  |  | HI | 5.37×10^-1^ | n.s. | n.s. | - | - | n.s. | n.s. | - | - |
|  |  |  | PP | 5.42×10^-1^ | n.s. | 4.85×10^-3^ | - | - | n.s. | n.s. | - | - |
|  |  |  | PB | 4.62×10^-1^ | n.s. | 1.27×10^-2^ | - | - | n.s. | n.s. | - | - |

The level of significance is 0.0066 after multiple comparisons correction, – = not applicable; n.s. = non-significant, HS = Hemisphere, L = left, R = right, EBP = emotional and behavioural problems, ES = Emotional Symptoms, CP =Conduct Problems , HI = Hyperactivity/Inattention, PP = Peer Relationship Problems, PB = Prosocial Behaviors

Table S5 Model parameters for fixed effects when including level of emotional and behavioural problems in the best fitting models for whole cerebellum and cerebeller subregion cortical thicknesses in boys.

| Cortical thickess | HS | Best fitting model | EBP Scales | Intercept | Site | Age coefficient  β2 | Age^2^ coefficient  β3 | Age^3^ coefficient  β3 | EBP | Age×EBP coefficient  β5 | Age^2^×EBP coefficient  β6 | Age^3^×EBP coefficient  β6 |
| --- | --- | --- | --- | --- | --- | --- | --- | --- | --- | --- | --- | --- |
| Cerebellum | L | Linear | ES | 4.83 | n.s. | 1.77×10^-2^ | - | - | n.s. | n.s. | - | - |
|  |  |  | CP | 4.80 | n.s. | 1.97×10^-2^ | - | - | n.s. | n.s. | - | - |
|  |  |  | HI | 4.83 | n.s. | 1.73×10^-2^ | - | - | n.s. | n.s. | - | - |
|  |  |  | PP | 4.84 | n.s. | 1.60×10^-2^ | - | - | n.s. | n.s. | - | - |
|  |  |  | PB | 4.70 | n.s. | 3.07×10^-2^ | - | - | n.s. | n.s. | - | - |
|  | R | Linear | ES | 4.84 | n.s. | 1.40×10^-2^ | - | - | n.s. | n.s. | - | - |
|  |  |  | CP | 4.82 | n.s. | 1.57×10^-2^ | - | - | n.s. | n.s. | - | - |
|  |  |  | HI | 4.84 | n.s. | 1.31×10^-2^ | - | - | n.s. | n.s. | - | - |
|  |  |  | PP | 4.89 | n.s. | 9.29×10^-3^ | - | - | n.s. | n.s. | - | - |
|  |  |  | PB | 4.66 | n.s. | 3.31×10^-2^ | - | - | n.s. | n.s. | - | - |
| Lobule Ⅰ-Ⅱ | L | None | ES | 2.36 | 9.46×10^-2^ | n.s. | - | - | n.s. | - | - | - |
|  |  |  | CP | 2.34 | 9.19×10^-2^ | n.s. | - | - | n.s. | - | - | - |
|  |  |  | HI | 2.32 | 9.21×10^-2^ | n.s. | - | - | n.s. | - | - | - |
|  |  |  | PP | 2.37 | 9.39×10^-2^ | n.s. | - | - | n.s. | - | - | - |
|  |  |  | PB | 2.38 | 9.49×10^-2^ | n.s. | - | - | n.s. | - | - | - |
|  | R | None | ES | 2.33 | 1.10×10^-1^ | n.s. | - | - | n.s. | - | - | - |
|  |  |  | CP | 2.31 | 1.09×10^-1^ | n.s. | - | - | n.s. | - | - | - |
|  |  |  | HI | 2.26 | 1.07×10^-1^ | n.s. | - | - | n.s. | - | - | - |
|  |  |  | PP | 2.33 | 1.10×10^-1^ | n.s. | - | - | n.s. | - | - | - |
|  |  |  | PB | 2.27 | 1.11×10^-1^ | n.s. | - | - | n.s. | - | - | - |
| Lobule Ⅲ | L | None | ES | 3.87 | n.s. | n.s. | - | - | n.s. | - | - | - |
|  |  |  | CP | 3.85 | n.s. | n.s. | - | - | n.s. | - | - | - |
|  |  |  | HI | 3.90 | n.s. | n.s. | - | - | n.s. | - | - | - |
|  |  |  | PP | 3.84 | n.s. | n.s. | - | - | n.s. | - | - | - |
|  |  |  | PB | 3.85 | n.s. | n.s. | - | - | n.s. | - | - | - |
|  | R | None | ES | 3.68 | n.s. | n.s. | - | - | n.s. | - | - | - |
|  |  |  | CP | 3.66 | n.s. | n.s. | - | - | n.s. | - | - | - |
|  |  |  | HI | 3.67 | n.s. | n.s. | - | - | n.s. | - | - | - |
|  |  |  | PP | 3.66 | n.s. | n.s. | - | - | n.s. | - | - | - |
|  |  |  | PB | 3.65 | n.s. | n.s. | - | - | n.s. | - | - | - |
| Lobule Ⅳ | L | Linear | ES | 5.21 | n.s. | n.s. | - | - | n.s. | n.s. | - | - |
|  |  |  | CP | 5.20 | n.s. | n.s. | - | - | n.s. | n.s. | - | - |
|  |  |  | HI | 5.21 | n.s. | n.s. | - | - | n.s. | n.s. | - | - |
|  |  |  | PP | 5.26 | n.s. | n.s. | - | - | n.s. | 4.02×10^-3^ | - | - |
|  |  |  | PB | 4.96 | n.s. | 3.19×10^-2^ | - | - | 3.23×10^-2^ | -3.43×10^-3^ | - | - |
|  | R | Linear | ES | 5.13 | n.s. | n.s. | - | - | n.s. | n.s. | - | - |
|  |  |  | CP | 5.16 | n.s. | n.s. | - | - | n.s. | n.s. | - | - |
|  |  |  | HI | 5.13 | n.s. | n.s. | - | - | n.s. | n.s. | - | - |
|  |  |  | PP | 5.18 | n.s. | n.s. | - | - | -4.02×10^-2^ | 4.20×10^-3^ | - | - |
|  |  |  | PB | 4.878 | n.s. | 3.01×10^-2^ | - | - | 3.12×10^-2^ | -3.16×10^-3^ | - | - |
| Lobule Ⅴ | L | Linear | ES | 5.18 | n.s. | 1.01×10^-2^ | - | - | n.s. | n.s. | - | - |
|  |  |  | CP | 5.16 | n.s. | 1.14×10^-2^ | - | - | n.s. | n.s. | - | - |
|  |  |  | HI | 5.18 | n.s. | n.s. | - | - | n.s. | n.s. | - | - |
|  |  |  | PP | 5.20 | n.s. | n.s. | - | - | n.s. | n.s. | - | - |
|  |  |  | PB | 4.94 | n.s. | 3.24×10^-2^ | - | - | 2.94×10^-2^ | n.s. | - | - |
|  | R | Linear | ES | 5.04 | n.s. | 1.04×10^-2^ | - | - | n.s. | n.s. | - | - |
|  |  |  | CP | 5.04 | n.s. | n.s. | - | - | n.s. | n.s. | - | - |
|  |  |  | HI | 5.03 | n.s. | n.s. | - | - | n.s. | n.s. | - | - |
|  |  |  | PP | 5.07 | n.s. | n.s. | - | - | n.s. | n.s. | - | - |
|  |  |  | PB | 4.78 | n.s. | 3.50×10^-2^ | - | - | 3.39×10^-2^ | n.s. | - | - |
| Lobule Ⅵ | L | Linear | ES | 5.18 | n.s. | 1.05×10^-2^ | - | - | n.s. | n.s. | - | - |
|  |  |  | CP | 5.15 | n.s. | 1.19×10^-2^ | - | - | n.s. | n.s. | - | - |
|  |  |  | HI | 5.19 | n.s. | n.s. | - | - | n.s. | n.s. | - | - |
|  |  |  | PP | 5.21 | n.s. | n.s. | - | - | n.s. | n.s. | - | - |
|  |  |  | PB | 5.05 | n.s. | 2.37×10^-2^ | - | - | n.s. | n.s. | - | - |
|  | R | Linear | ES | 5.11 | n.s. | 9.27×10^-3^ | - | - | n.s. | n.s. | - | - |
|  |  |  | CP | 5.17 | n.s. | n.s. | - | - | n.s. | n.s. | - | - |
|  |  |  | HI | 5.12 | n.s. | n.s. | - | - | n.s. | n.s. | - | - |
|  |  |  | PP | 5.13 | n.s. | n.s. | - | - | n.s. | n.s. | - | - |
|  |  |  | PB | 4.92 | n.s. | 2.89×10^-2^ | - | - | 2.66×10^-2^ | -2.73×10^-3^ | - | - |
| Lobule  Crus I | L | Linear | ES | 4.68 | n.s. | 2.48×10^-2^ | - | - | n.s. | n.s. | - | - |
|  |  |  | CP | 4.64 | n.s. | 2.75×10^-2^ | - | - | n.s. | n.s. | - | - |
|  |  |  | HI | 4.65 | n.s. | 2.66×10^-2^ | - | - | n.s. | n.s. | - | - |
|  |  |  | PP | 4.62 | n.s. | 2.98×10^-2^ | - | - | n.s. | n.s. | - | - |
|  |  |  | PB | 4.57 | n.s. | 3.55×10^-2^ | - | - | n.s. | n.s. | - | - |
|  | R | Linear | ES | 4.76 | n.s. | 1.76×10^-2^ | - | - | n.s. | n.s. | - | - |
|  |  |  | CP | 4.70 | n.s. | 2.26×10^-2^ | - | - | n.s. | n.s. | - | - |
|  |  |  | HI | 4.75 | n.s. | 1.69×10^-2^ | - | - | n.s. | n.s. | - | - |
|  |  |  | PP | 4.80 | n.s. | n.s. | - | - | n.s. | n.s. | - | - |
|  |  |  | PB | 4.59 | n.s. | 3.55×10^-2^ | - | - | n.s. | n.s. | - | - |
| Lobule  Crus II | L | Linear | ES | 4.53 | n.s. | 3.51×10^-2^ | - | - | n.s. | n.s. | - | - |
|  |  |  | CP | 4.50 | n.s. | 3.75×10^-2^ | - | - | n.s. | n.s. | - | - |
|  |  |  | HI | 4.56 | n.s. | 3.23×10^-2^ | - | - | n.s. | n.s. | - | - |
|  |  |  | PP | 4.48 | n.s. | 3.87×10^-2^ | - | - | n.s. | n.s. | - | - |
|  |  |  | PB | 4.58 | n.s. | n.s. | - | - | n.s. | n.s. | - | - |
|  | R | Linear | ES | 4.74 | n.s. | 2.49×10^-2^ | - | - | n.s. | n.s. | - | - |
|  |  |  | CP | 4.74 | n.s. | 2.45×10^-2^ | - | - | n.s. | n.s. | - | - |
|  |  |  | HI | 4.74 | n.s. | 2.37×10^-2^ | - | - | n.s. | n.s. | - | - |
|  |  |  | PP | 4.75 | n.s. | 2.32×10^-2^ | - | - | n.s. | n.s. | - | - |
|  |  |  | PB | 4.61 | n.s. | 4.02×10^-2^ | - | - | n.s. | n.s. | - | - |
| Lobule ⅦB | L | Linear | ES | 4.91 | n.s. | 1.63×10^-2^ | - | - | n.s. | n.s. | - | - |
|  |  |  | CP | 4.91 | n.s. | 1.64×10^-2^ | - | - | n.s. | n.s. | - | - |
|  |  |  | HI | 4.93 | n.s. | n.s. | - | - | n.s. | n.s. | - | - |
|  |  |  | PP | 4.90 | n.s. | 1.69×10^-2^ | - | - | n.s. | n.s. | - | - |
|  |  |  | PB | 4.80 | n.s. | n.s. | - | - | n.s. | n.s. | - | - |
|  | R | Linear | ES | 4.97 | n.s. | 1.17×10^-2^ | - | - | n.s. | n.s. | - | - |
|  |  |  | CP | 4.90 | n.s. | 1.82×10^-2^ | - | - | n.s. | n.s. | - | - |
|  |  |  | HI | 4.93 | n.s. | 1.52×10^-2^ | - | - | n.s. | n.s. | - | - |
|  |  |  | PP | 5.01 | n.s. | n.s. | - | - | n.s. | n.s. | - | - |
|  |  |  | PB | 4.72 | n.s. | 3.48×10^-2^ | - | - | n.s. | n.s. | - | - |
| Lobule ⅧA | L | Linear | ES | 4.91 | n.s. | 1.14×10^-2^ | - | - | n.s. | n.s. | - | - |
|  |  |  | CP | 4.90 | n.s. | n.s. | - | - | n.s. | n.s. | - | - |
|  |  |  | HI | 4.88 | n.s. | n.s. | - | - | n.s. | n.s. | - | - |
|  |  |  | PP | 4.94 | n.s. | n.s. | - | - | n.s. | n.s. | - | - |
|  |  |  | PB | 4.82 | n.s. | n.s. | - | - | n.s. | n.s. | - | - |
|  | R | Linear | ES | 4.79 | n.s. | 1.47×10^-2^ | - | - | n.s. | n.s. | - | - |
|  |  |  | CP | 4.75 | n.s. | 1.81×10^-2^ | - | - | n.s. | n.s. | - | - |
|  |  |  | HI | 4.80 | n.s. | n.s. | - | - | n.s. | n.s. | - | - |
|  |  |  | PP | 4.83 | n.s. | n.s. | - | - | n.s. | n.s. | - | - |
|  |  |  | PB | 4.73 | n.s. | n.s. | - | - | n.s. | n.s. | - | - |
| Lobule ⅧB | L | Linear | ES | 4.92 | n.s. | 1.79×10^-2^ | - | - | n.s. | n.s. | - | - |
|  |  |  | CP | 4.85 | n.s. | 2.34×10^-2^ | - | - | n.s. | n.s. | - | - |
|  |  |  | HI | 4.90 | n.s. | 1.92×10^-2^ | - | - | n.s. | n.s. | - | - |
|  |  |  | PP | 5.01 | n.s. | n.s. | - | - | n.s. | n.s. | - | - |
|  |  |  | PB | 4.69 | n.s. | 4.31×10^-2^ | - | - | n.s. | n.s. | - | - |
|  | R | None | ES | 4.92 | n.s. | n.s. | - | - | n.s. | - | - | - |
|  |  |  | CP | 4.91 | n.s. | n.s. | - | - | n.s. | - | - | - |
|  |  |  | HI | 4.91 | n.s. | n.s. | - | - | n.s. | - | - | - |
|  |  |  | PP | 4.92 | n.s. | n.s. | - | - | n.s. | - | - | - |
|  |  |  | PB | 4.95 | n.s. | n.s. | - | - | n.s. | - | - | - |
| Lobule Ⅸ | L | None | ES | 4.68 | n.s. | n.s. | - | - | n.s. | - | - | - |
|  |  |  | CP | 4.66 | n.s. | n.s. | - | - | n.s. | - | - | - |
|  |  |  | HI | 4.65 | n.s. | n.s. | - | - | n.s. | - | - | - |
|  |  |  | PP | 4.68 | n.s. | n.s. | - | - | n.s. | - | - | - |
|  |  |  | PB | 4.68 | n.s. | n.s. | - | - | n.s. | - | - | - |
|  | R | None | ES | 4.75 | n.s. | n.s. | - | - | n.s. | - | - | - |
|  |  |  | CP | 4.76 | n.s. | n.s. | - | - | n.s. | - | - | - |
|  |  |  | HI | 4.76 | n.s. | n.s. | - | - | n.s. | - | - | - |
|  |  |  | PP | 4.78 | n.s. | n.s. | - | - | n.s. | - | - | - |
|  |  |  | PB | 4.80 | n.s. | n.s. | - | - | n.s. | - | - | - |
| Lobule Ⅹ | L | Linear | ES | 3.52 | n.s. | 3.63×10^-2^ | - | - | n.s. | n.s. | - | - |
|  |  |  | CP | 3.49 | n.s. | 3.99×10^-2^ | - | - | n.s. | n.s. | - | - |
|  |  |  | HI | 3.42 | n.s. | 4.70×10^-2^ | - | - | n.s. | n.s. | - | - |
|  |  |  | PP | 3.69 | n.s. | n.s. | - | - | n.s. | n.s. | - | - |
|  |  |  | PB | 3.33 | n.s. | n.s. | - | - | n.s. | n.s. | - | - |
|  | R | Linear | ES | 2.85 | n.s. | 4.32×10^-2^ | - | - | n.s. | n.s. | - | - |
|  |  |  | CP | 2.70 | n.s. | 6.04×10^-2^ | - | - | n.s. | n.s. | - | - |
|  |  |  | HI | 2.95 | n.s. | n.s. | - | - | n.s. | n.s. | - | - |
|  |  |  | PP | 3.26 | n.s. | n.s. | - | - | -1.92×10^-1^ | 1.89×10^-2^ | - | - |
|  |  |  | PB | 2.42 | n.s. | 9.22×10^-2^ | - | - | n.s. | n.s. | - | - |

The level of significance is 0.0065 after multiple comparisons correction, – = not applicable; n.s. = non-significant, HS = Hemisphere, L = left, R = right, EBP = emotional and behavioural problems, ES = Emotional Symptoms, CP =Conduct Problems , HI = Hyperactivity/Inattention, PP = Peer Relationship Problems, PB = Prosocial Behaviors

Table S6 Model parameters for fixed effects when including level of emotional and behavioural problems in the best fitting models for whole cerebellum and cerebeller subregion surface areas in boys.

| Surface area | HS | Best fitting model | EBP Scales | Intercept | Site | Age coefficient  β2 | Age^2^ coefficient  β3 | Age^3^ coefficient  β3 | EBP | Age×EBP coefficient  β5 | Age^2^×EBP coefficient  β6 | Age^3^×EBP coefficient  β6 |
| --- | --- | --- | --- | --- | --- | --- | --- | --- | --- | --- | --- | --- |
| Cerebellum | L | Quadratic | ES | 9.83 | n.s. | 2.04×10^-1^ | -8.22×10^-3^ | - | n.s. | n.s. | n.s. | - |
|  |  |  | CP | 1.06×10 | n.s. | n.s. | n.s. | - | n.s. | n.s. | n.s. | - |
|  |  |  | HI | 1.03×10 | n.s. | n.s. | n.s. | - | n.s. | n.s. | n.s. | - |
|  |  |  | PP | 1.02×10 | n.s. | n.s. | n.s. | - | n.s. | n.s. | n.s. | - |
|  |  |  | PB | 8.23 | n.s. | 5.33×10^-1^ | -2.51×10^-2^ | - | n.s. | -5.36×10^-2^ | 2.69×10^-3^ | - |
|  | R | Linear | ES | 1.05×10 | n.s. | 5.17×10^-2^ | - | - | n.s. | n.s. | - | - |
|  |  |  | CP | 1.06×10 | n.s. | 4.34×10^-2^ | - | - | n.s. | n.s. | - | - |
|  |  |  | HI | 1.06×10 | n.s. | 4.33×10^-2^ | - | - | n.s. | n.s. | - | - |
|  |  |  | PP | 1.06×10 | n.s. | 5.00×10^-2^ | - | - | n.s. | n.s. | - | - |
|  |  |  | PB | 1.07×10 | n.s. | n.s. | - | - | n.s. | n.s. | - | - |
| Lobule Ⅰ-Ⅱ | L | None | ES | 1.20×10^-2^ | n.s. | n.s. | - | - | n.s. | - | - | - |
|  |  |  | CP | 1.19×10^-2^ | n.s. | n.s. | - | - | n.s. | - | - | - |
|  |  |  | HI | 1.20×10^-2^ | n.s. | n.s. | - | - | n.s. | - | - | - |
|  |  |  | PP | 1.20×10^-2^ | n.s. | n.s. | - | - | n.s. | - | - | - |
|  |  |  | PB | 1.15×10^-2^ | n.s. | n.s. | - | - | n.s. | - | - | - |
|  | R | None | ES | 1.70×10^-2^ | n.s. | n.s. | - | - | n.s. | - | - | - |
|  |  |  | CP | 1.73×10^-2^ | n.s. | n.s. | - | - | n.s. | - | - | - |
|  |  |  | HI | 1.76×10^-2^ | n.s. | n.s. | - | - | n.s. | - | - | - |
|  |  |  | PP | 1.74×10^-2^ | n.s. | n.s. | - | - | n.s. | - | - | - |
|  |  |  | PB | 1.68×10^-2^ | n.s. | n.s. | - | - | n.s. | - | - | - |
| Lobule Ⅲ | L | Linear | ES | 1.41×10^-1^ | n.s. | 1.02×10^-3^ | - | - | n.s. | n.s. | - | - |
|  |  |  | CP | 1.47×10^-1^ | n.s. | n.s. | - | - | n.s. | n.s. | - | - |
|  |  |  | HI | 1.39×10^-1^ | n.s. | n.s. | - | - | n.s. | n.s. | - | - |
|  |  |  | PP | 1.43×10^-1^ | n.s. | n.s. | - | - | n.s. | n.s. | - | - |
|  |  |  | PB | 1.47×10^-1^ | n.s. | n.s. | - | - | n.s. | n.s. | - | - |
|  | R | Linear | ES | 1.44×10^-1^ | n.s. | 9.93×10^-4^ | - | - | n.s. | n.s. | - | - |
|  |  |  | CP | 1.50×10^-1^ | n.s. | n.s. | - | - | n.s. | n.s. | - | - |
|  |  |  | HI | 1.47×10^-1^ | n.s. | n.s. | - | - | n.s. | n.s. | - | - |
|  |  |  | PP | 1.46×10^-1^ | n.s. | n.s. | - | - | n.s. | n.s. | - | - |
|  |  |  | PB | 1.50×10^-1^ | n.s. | n.s. | - | - | n.s. | n.s. | - | - |
| Lobule Ⅳ | L | Linear | ES | 4.48×10^-1^ | -1.53×10^-2^ | 1.76×10^-3^ | - | - | n.s. | n.s. | - | - |
|  |  |  | CP | 4.43×10^-1^ | -1.53×10^-2^ | 2.21×10^-3^ | - | - | n.s. | n.s. | - | - |
|  |  |  | HI | 4.37×10^-1^ | -1.54×10^-2^ | 2.72×10^-3^ | - | - | n.s. | n.s. | - | - |
|  |  |  | PP | 4.45×10^-1^ | -1.53×10^-2^ | n.s. | - | - | n.s. | n.s. | - | - |
|  |  |  | PB | 4.73×10^-1^ | -1.52×10^-2^ | n.s. | - | - | n.s. | n.s. | - | - |
|  | R | Linear | ES | 4.39×10^-1^ | -1.33×10^-2^ | 3.08×10^-3^ | - | - | n.s. | n.s. | - | - |
|  |  |  | CP | 4.43×10^-1^ | -1.32×10^-2^ | 2.72×10^-3^ | - | - | n.s. | n.s. | - | - |
|  |  |  | HI | 4.50×10^-1^ | -1.32×10^-2^ | n.s. | - | - | n.s. | n.s. | - | - |
|  |  |  | PP | 4.39×10^-1^ | -1.33×10^-2^ | 3.19×10^-3^ | - | - | n.s. | n.s. | - | - |
|  |  |  | PB | 4.63×10^-1^ | -1.31×10^-2^ | n.s. | - | - | n.s. | n.s. | - | - |
| Lobule Ⅴ | L | Linear | ES | 6.92×10^-1^ | n.s. | 6.55×10^-3^ | - | - | n.s. | n.s. | - | - |
|  |  |  | CP | 7.05×10^-1^ | n.s. | 5.31×10^-3^ | - | - | n.s. | n.s. | - | - |
|  |  |  | HI | 7.03×10^-1^ | n.s. | 5.48×10^-3^ | - | - | n.s. | n.s. | - | - |
|  |  |  | PP | 6.94×10^-1^ | n.s. | 6.32×10^-3^ | - | - | n.s. | n.s. | - | - |
|  |  |  | PB | 7.09×10^-1^ | n.s. | n.s. | - | - | n.s. | n.s. | - | - |
|  | R | Linear | ES | 7.02×10^-1^ | n.s. | 3.72×10^-3^ | - | - | n.s. | n.s. | - | - |
|  |  |  | CP | 6.98×10^-1^ | n.s. | 4.05×10^-3^ | - | - | n.s. | n.s. | - | - |
|  |  |  | HI | 7.11×10^-1^ | n.s. | n.s. | - | - | n.s. | n.s. | - | - |
|  |  |  | PP | 7.00×10^-1^ | n.s. | 3.93×10^-3^ | - | - | n.s. | n.s. | - | - |
|  |  |  | PB | 7.32×10^-1^ | n.s. | n.s. | - | - | n.s. | n.s. | - | - |
| Lobule Ⅵ | L | Quadratic | ES | 1.44 | n.s. | 4.12×10^-2^ | -1.55×10^-3^ | - | n.s. | n.s. | n.s. | - |
|  |  |  | CP | 1.52 | n.s. | n.s. | n.s. | - | n.s. | n.s. | n.s. | - |
|  |  |  | HI | 1.48 | n.s. | n.s. | n.s. | - | n.s. | n.s. | n.s. | - |
|  |  |  | PP | 1.44 | n.s. | n.s. | n.s. | - | n.s. | n.s. | n.s. | - |
|  |  |  | PB | 1.24 | n.s. | n.s. | n.s. | - | n.s. | n.s. | n.s. | - |
|  | R | Linear | ES | 1.50 | n.s. | 1.38×10^-2^ | - | - | n.s. | n.s. | - | - |
|  |  |  | CP | 1.49 | n.s. | 1.45×10^-2^ | - | - | n.s. | n.s. | - | - |
|  |  |  | HI | 1.49 | n.s. | 1.47×10^-2^ | - | - | n.s. | n.s. | - | - |
|  |  |  | PP | 1.49 | n.s. | 1.45×10^-2^ | - | - | n.s. | n.s. | - | - |
|  |  |  | PB | 1.50 | n.s. | 1.31×10^-2^ | - | - | n.s. | n.s. | - | - |
| Lobule  Crus I | L | None | ES | 2.39 | n.s. | n.s. | - | - | n.s. | - | - | - |
|  |  |  | CP | 2.39 | n.s. | n.s. | - | - | n.s. | - | - | - |
|  |  |  | HI | 2.38 | n.s. | n.s. | - | - | n.s. | - | - | - |
|  |  |  | PP | 2.39 | n.s. | n.s. | - | - | n.s. | - | - | - |
|  |  |  | PB | 2.37 | n.s. | n.s. | - | - | n.s. | - | - | - |
|  | R | Linear | ES | 2.30 | n.s. | 1.10×10^-2^ | - | - | n.s. | n.s. | - | - |
|  |  |  | CP | 2.33 | n.s. | n.s. | - | - | n.s. | n.s. | - | - |
|  |  |  | HI | 2.27 | n.s. | 1.28×10^-2^ | - | - | n.s. | n.s. | - | - |
|  |  |  | PP | 2.29 | n.s. | 1.16×10^-2^ | - | - | n.s. | n.s. | - | - |
|  |  |  | PB | 2.36 | n.s. | n.s. | - | - | n.s. | n.s. | - | - |
| Lobule  Crus II | L | None | ES | 1.75 | n.s. | n.s. | - | - | n.s. | - | - | - |
|  |  |  | CP | 1.75 | n.s. | n.s. | - | - | n.s. | - | - | - |
|  |  |  | HI | 1.73 | n.s. | n.s. | - | - | n.s. | - | - | - |
|  |  |  | PP | 1.74 | n.s. | n.s. | - | - | n.s. | - | - | - |
|  |  |  | PB | 1.77 | n.s. | n.s. | - | - | n.s. | - | - | - |
|  | R | None | ES | 1.78 | n.s. | n.s. | - | - | n.s. | - | - | - |
|  |  |  | CP | 1.78 | n.s. | n.s. | - | - | n.s. | - | - | - |
|  |  |  | HI | 1.76 | n.s. | n.s. | - | - | n.s. | - | - | - |
|  |  |  | PP | 1.77 | n.s. | n.s. | - | - | n.s. | - | - | - |
|  |  |  | PB | 1.77 | n.s. | n.s. | - | - | n.s. | - | - | - |
| Lobule ⅦB | L | None | ES | 8.89×10^-1^ | n.s. | n.s. | - | - | n.s. | - | - | - |
|  |  |  | CP | 8.90×10^-1^ | n.s. | n.s. | - | - | n.s. | - | - | - |
|  |  |  | HI | 8.92×10^-1^ | n.s. | n.s. | - | - | n.s. | - | - | - |
|  |  |  | PP | 8.87×10^-1^ | n.s. | n.s. | - | - | n.s. | - | - | - |
|  |  |  | PB | 8.94×10^-1^ | n.s. | n.s. | - | - | n.s. | - | - | - |
|  | R | Linear | ES | 8.96×10^-1^ | n.s. | 5.46×10^-3^ | - | - | n.s. | n.s. | - | - |
|  |  |  | CP | 9.23×10^-1^ | n.s. | n.s. | - | - | n.s. | n.s. | - | - |
|  |  |  | HI | 9.01×10^-1^ | n.s. | n.s. | - | - | n.s. | n.s. | - | - |
|  |  |  | PP | 9.21×10^-1^ | n.s. | n.s. | - | - | n.s. | n.s. | - | - |
|  |  |  | PB | 8.76×10^-1^ | n.s. | n.s. | - | - | n.s. | n.s. | - | - |
| Lobule ⅧA | L | Linear | ES | 1.17 | n.s. | 6.99×10^-3^ | - | - | n.s. | n.s. | - | - |
|  |  |  | CP | 1.18 | n.s. | n.s. | - | - | n.s. | n.s. | - | - |
|  |  |  | HI | 1.19 | n.s. | n.s. | - | - | n.s. | n.s. | - | - |
|  |  |  | PP | 1.16 | n.s. | 8.80×10^-3^ | - | - | n.s. | n.s. | - | - |
|  |  |  | PB | 1.20 | n.s. | n.s. | - | - | n.s. | n.s. | - | - |
|  | R | Linear | ES | 1.15 | n.s. | 5.96×10^-3^ | - | - | n.s. | n.s. | - | - |
|  |  |  | CP | 1.15 | n.s. | n.s. | - | - | n.s. | n.s. | - | - |
|  |  |  | HI | 1.21 | n.s. | n.s. | - | - | -1.81×10^-2^ | 1.78×10^-3^ | - | - |
|  |  |  | PP | 1.17 | n.s. | n.s. | - | - | n.s. | n.s. | - | - |
|  |  |  | PB | 1.10 | n.s. | n.s. | - | - | n.s. | n.s. | - | - |
| Lobule ⅧB | L | Linear | ES | 7.44×10^-1^ | n.s. | 5.47×10^-3^ | - | - | n.s. | n.s. | - | - |
|  |  |  | CP | 7.65×10^-1^ | n.s. | n.s. | - | - | n.s. | n.s. | - | - |
|  |  |  | HI | 7.60×10^-1^ | n.s. | n.s. | - | - | n.s. | n.s. | - | - |
|  |  |  | PP | 7.53×10^-1^ | n.s. | n.s. | - | - | n.s. | n.s. | - | - |
|  |  |  | PB | 6.82×10^-1^ | n.s. | n.s. | - | - | n.s. | n.s. | - | - |
|  | R | Linear | ES | 7.20×10^-1^ | n.s. | 6.76×10^-3^ | - | - | n.s. | n.s. | - | - |
|  |  |  | CP | 7.44×10^-1^ | n.s. | 4.61×10^-3^ | - | - | n.s. | n.s. | - | - |
|  |  |  | HI | 7.46×10^-1^ | n.s. | n.s. | - | - | n.s. | n.s. | - | - |
|  |  |  | PP | 7.26×10^-1^ | n.s. | 6.14×10^-3^ | - | - | n.s. | n.s. | - | - |
|  |  |  | PB | 7.07×10^-1^ | n.s. | n.s. | - | - | n.s. | n.s. | - | - |
| Lobule Ⅸ | L | Linear | ES | 7.25×10^-1^ | -2.61×10^-2^ | 3.23×10^-3^ | - | - | n.s. | n.s. | - | - |
|  |  |  | CP | 7.22×10^-1^ | -2.58×10^-2^ | 3.73×10^-3^ | - | - | n.s. | n.s. | - | - |
|  |  |  | HI | 7.34×10^-1^ | -2.59×10^-2^ | n.s. | - | - | n.s. | n.s. | - | - |
|  |  |  | PP | 7.18×10^-1^ | -2.60×10^-2^ | 4.07×10^-3^ | - | - | n.s. | n.s. | - | - |
|  |  |  | PB | 7.49×10^-1^ | -2.59×10^-2^ | n.s. | - | - | n.s. | n.s. | - | - |
|  | R | Linear | ES | 7.47×10^-1^ | -2.48×10^-2^ | 4.02×10^-3^ | - | - | n.s. | n.s. | - | - |
|  |  |  | CP | 7.53×10^-1^ | -2.45×10^-2^ | 3.48×10^-3^ | - | - | n.s. | n.s. | - | - |
|  |  |  | HI | 7.59×10^-1^ | -2.45×10^-2^ | n.s. | - | - | n.s. | n.s. | - | - |
|  |  |  | PP | 7.43×10^-1^ | -2.47×10^-2^ | 4.20×10^-3^ | - | - | n.s. | n.s. | - | - |
|  |  |  | PB | 7.59×10^-1^ | -2.47×10^-2^ | n.s. | - | - | n.s. | n.s. | - | - |
| Lobule Ⅹ | L | None | ES | 1.50×10^-1^ | -3.99×10^-3^ | n.s. | - | - | n.s. | - | - | - |
|  |  |  | CP | 1.50×10^-1^ | -4.00×10^-3^ | n.s. | - | - | n.s. | - | - | - |
|  |  |  | HI | 1.50×10^-1^ | -4.02×10^-3^ | n.s. | - | - | n.s. | - | - | - |
|  |  |  | PP | 1.51×10^-1^ | -3.99×10^-3^ | n.s. | - | - | n.s. | - | - | - |
|  |  |  | PB | 1.52×10^-1^ | -4.00×10^-3^ | n.s. | - | - | n.s. | - | - | - |
|  | R | None | ES | 1.82×10^-1^ | n.s. | n.s. | - | - | n.s. | - | - | - |
|  |  |  | CP | 1.82×10^-1^ | n.s. | n.s. | - | - | n.s. | - | - | - |
|  |  |  | HI | 1.82×10^-1^ | n.s. | n.s. | - | - | n.s. | - | - | - |
|  |  |  | PP | 1.81×10^-1^ | n.s. | n.s. | - | - | n.s. | - | - | - |
|  |  |  | PB | 1.81×10^-1^ | n.s. | n.s. | - | - | n.s. | - | - | - |

The level of significance is 0.0056 after multiple comparisons correction, – = not applicable; n.s. = non-significant, HS = Hemisphere, L = left, R = right, EBP = emotional and behavioural problems, ES = Emotional Symptoms, CP =Conduct Problems , HI = Hyperactivity/Inattention, PP = Peer Relationship Problems, PB = Prosocial Behaviors

Table S7 Model parameters for fixed effects when including level of emotional and behavioural problems in the best fitting models for whole cerebellum and cerebeller subregion gray matter volumes in girls.

| Gray matter volume | HS | Best fitting model | EBP Scales | Intercept | Site | Age coefficient  β2 | Age^2^ coefficient  β3 | Age^3^ coefficient  β3 | EBP | Age×EBP coefficient  β5 | Age^2^×EBP coefficient  β6 | Age^3^×EBP coefficient  β6 |
| --- | --- | --- | --- | --- | --- | --- | --- | --- | --- | --- | --- | --- |
| Cerebellum | L | Cubic | ES | 5.10×10 | n.s. | n.s. | n.s. | n.s. | n.s. | n.s. | n.s. | n.s. |
|  |  |  | CP | 7.18×10 | n.s. | -8.14 | 9.26×10^-1^ | -3.30×10^-2^ | -8.99 | 2.87 | -2.99×10^-1^ | 1.02×10^-2^ |
|  |  |  | HI | 5.40×10 | n.s. | n.s. | n.s. | n.s. | n.s. | n.s. | n.s. | n.s. |
|  |  |  | PP | 5.43×10 | n.s. | n.s. | n.s. | -1.55×10^-2^ | n.s. | n.s. | n.s. | n.s. |
|  |  |  | PB | n.s. | n.s. | n.s. | n.s. | n.s. | n.s. | n.s. | n.s. | n.s. |
|  | R | Cubic | ES | 5.39×10 | n.s. | n.s. | n.s. | -1.48×10^-2^ | n.s. | n.s. | n.s. | n.s. |
|  |  |  | CP | 7.38×10 | n.s. | -8.79 | 9.80×10^-1^ | -3.43×10^-2^ | -1.00×10 | 3.11 | -3.15×10^-1^ | 1.04×10^-2^ |
|  |  |  | HI | 5.82×10 | n.s. | n.s. | n.s. | n.s. | n.s. | n.s. | n.s. | n.s. |
|  |  |  | PP | 4.97×10 | n.s. | n.s. | n.s. | n.s. | n.s. | n.s. | n.s. | n.s. |
|  |  |  | PB | n.s. | n.s. | n.s. | n.s. | n.s. | n.s. | n.s. | n.s. | n.s. |
| Lobule Ⅰ-Ⅱ | L | None | ES | 2.65×10^-2^ | n.s. | n.s. | - | - | n.s. | - | - | - |
|  |  |  | CP | 2.71×10^-2^ | n.s. | n.s. | - | - | n.s. | - | - | - |
|  |  |  | HI | 2.73×10^-2^ | n.s. | n.s. | - | - | n.s. | - | - | - |
|  |  |  | PP | 2.61×10^-2^ | n.s. | n.s. | - | - | n.s. | - | - | - |
|  |  |  | PB | 2.90×10^-2^ | n.s. | n.s. | - | - | n.s. | - | - | - |
|  | R | None | ES | 3.91×10^-2^ | n.s. | n.s. | - | - | n.s. | - | - | - |
|  |  |  | CP | 3.91×10^-2^ | n.s. | n.s. | - | - | n.s. | - | - | - |
|  |  |  | HI | 4.00×10^-2^ | n.s. | n.s. | - | - | n.s. | - | - | - |
|  |  |  | PP | 3.86×10^-2^ | n.s. | n.s. | - | - | n.s. | - | - | - |
|  |  |  | PB | 4.13×10^-2^ | n.s. | n.s. | - | - | n.s. | - | - | - |
| Lobule Ⅲ | L | Linear | ES | 4.89×10^-1^ | n.s. | 5.91×10^-3^ | - | - | n.s. | n.s. | - | - |
|  |  |  | CP | 4.83×10^-1^ | n.s. | 6.07×10^-3^ | - | - | n.s. | n.s. | - | - |
|  |  |  | HI | 4.94×10^-1^ | n.s. | n.s. | - | - | n.s. | n.s. | - | - |
|  |  |  | PP | 4.86×10^-1^ | n.s. | 6.13×10^-3^ | - | - | n.s. | n.s. | - | - |
|  |  |  | PB | 5.46×10^-1^ | n.s. | n.s. | - | - | n.s. | n.s. | - | - |
|  | R | Linear | ES | 4.85×10^-1^ | n.s. | 4.53×10^-3^ | - | - | n.s. | n.s. | - | - |
|  |  |  | CP | 4.70×10^-1^ | n.s. | 5.37×10^-3^ | - | - | n.s. | n.s. | - | - |
|  |  |  | HI | 4.85×10^-1^ | n.s. | n.s. | - | - | n.s. | n.s. | - | - |
|  |  |  | PP | 4.93×10^-1^ | n.s. | n.s. | - | - | n.s. | n.s. | - | - |
|  |  |  | PB | 4.96×10^-1^ | n.s. | n.s. | - | - | n.s. | n.s. | - | - |
| Lobule Ⅳ | L | Quadratic | ES | 1.76 | n.s. | 7.95×10^-2^ | -3.64×10^-3^ | - | n.s. | n.s. | n.s. | - |
|  |  |  | CP | 1.68 | n.s. | 9.21×10^-2^ | -4.09×10^-3^ | - | n.s. | n.s. | n.s. | - |
|  |  |  | HI | 1.66 | n.s. | 9.41×10^-2^ | n.s. | - | n.s. | n.s. | n.s. | - |
|  |  |  | PP | 1.79 | n.s. | n.s. | n.s. | - | n.s. | n.s. | n.s. | - |
|  |  |  | PB | 1.77 | n.s. | n.s. | n.s. | - | n.s. | n.s. | n.s. | - |
|  | R | Quadratic | ES | 1.73 | n.s. | 7.55×10^-2^ | -3.23×10^-3^ | - | n.s. | n.s. | n.s. | - |
|  |  |  | CP | 1.55 | n.s. | 1.12×10^-1^ | -5.00×10^-3^ | - | n.s. | n.s. | n.s. | - |
|  |  |  | HI | 1.74 | n.s. | n.s. | n.s. | - | n.s. | n.s. | n.s. | - |
|  |  |  | PP | 1.77 | n.s. | n.s. | n.s. | - | n.s. | n.s. | n.s. | - |
|  |  |  | PB | 1.99 | n.s. | n.s. | n.s. | - | n.s. | n.s. | n.s. | - |
| Lobule Ⅴ | L | Linear | ES | 3.48 | n.s. | 2.75×10^-2^ | - | - | n.s. | n.s. | - | - |
|  |  |  | CP | 3.42 | n.s. | 3.22×10^-2^ | - | - | n.s. | n.s. | - | - |
|  |  |  | HI | 3.55 | n.s. | 2.07×10^-2^ | - | - | -4.31×10^-2^ | 4.31×10^-3^ | - | - |
|  |  |  | PP | 3.50 | n.s. | 2.66×10^-2^ | - | - | n.s. | n.s. | - | - |
|  |  |  | PB | 3.37 | n.s. | 3.61×10^-2^ | - | - | n.s. | n.s. | - | - |
|  | R | Linear | ES | 3.14 | n.s. | 2.90×10^-2^ | - | - | n.s. | n.s. | - | - |
|  |  |  | CP | 3.13 | n.s. | 2.97×10^-2^ | - | - | n.s. | n.s. | - | - |
|  |  |  | HI | 3.14 | n.s. | 3.00×10^-2^ | - | - | n.s. | n.s. | - | - |
|  |  |  | PP | 3.16 | n.s. | 2.75×10^-2^ | - | - | n.s. | n.s. | - | - |
|  |  |  | PB | 2.94 | n.s. | 4.04×10^-2^ | - | - | n.s. | n.s. | - | - |
| Lobule Ⅵ | L | Cubic | ES | 9.12 | n.s. | n.s. | n.s. | n.s. | n.s. | n.s. | n.s. | n.s. |
|  |  |  | CP | 1.29×10 | n.s. | -1.70 | 1.92×10^-1^ | -6.88×10^-3^ | -2.15 | 6.68×10^-1^ | -6.86×10^-2^ | 2.32×10^-3^ |
|  |  |  | HI | 6.72 | n.s. | n.s. | n.s. | n.s. | n.s. | n.s. | n.s. | n.s. |
|  |  |  | PP | 7.46 | n.s. | n.s. | n.s. | n.s. | n.s. | n.s. | n.s. | n.s. |
|  |  |  | PB | 1.22×10 | n.s. | n.s. | n.s. | n.s. | n.s. | n.s. | n.s . | n.s. |
|  | R | Cubic | ES | 7.46 | n.s. | n.s. | n.s. | n.s. | n.s. | n.s. | n.s. | n.s. |
|  |  |  | CP | 1.30×10 | n.s. | -1.85 | 2.09×10^-1^ | -7.44×10^-3^ | -2.22 | 7.13×10^-1^ | -7.48×10^-2^ | 2.57×10^-3^ |
|  |  |  | HI | 7.62 | n.s. | n.s. | n.s. | n.s. | n.s. | n.s. | n.s. | n.s. |
|  |  |  | PP | 7.68 | n.s. | n.s. | n.s. | n.s. | n.s. | n.s. | n.s. | n.s. |
|  |  |  | PB | n.s. | n.s. | n.s. | n.s. | n.s. | n.s. | n.s. | n.s. | n.s. |
| Lobule  Crus I | L | Cubic | ES | 1.18×10 | n.s. | n.s. | n.s. | n.s. | n.s. | n.s. | n.s. | n.s. |
|  |  |  | CP | 1.87×10 | n.s. | -2.79 | 3.05×10^-1^ | -1.07×10^-2^ | n.s. | 1.01 | -1.04×10^-1^ | 3.46×10^-3^ |
|  |  |  | HI | 1.42×10 | n.s. | n.s. | n.s. | n.s. | n.s. | n.s. | n.s. | n.s. |
|  |  |  | PP | 1.44×10 | n.s. | n.s. | n.s. | -6.42×10^-3^ | n.s. | n.s. | n.s. | n.s. |
|  |  |  | PB | n.s. | n.s. | n.s. | n.s. | n.s. | n.s. | n.s. | n.s. | n.s. |
|  | R | Quadratic | ES | 7.20 | n.s. | 6.02×10^-1^ | -2.70×10^-2^ | - | n.s. | n.s. | n.s. | - |
|  |  |  | CP | 7.30 | n.s. | 5.87×10^-1^ | -2.66×10^-2^ | - | n.s. | n.s. | n.s. | - |
|  |  |  | HI | 7.11 | n.s. | 6.19×10^-1^ | -2.76×10^-2^ | - | n.s. | n.s. | n.s. | - |
|  |  |  | PP | 6.59 | n.s. | 7.23×10^-1^ | -3.32×10^-2^ | - | n.s. | -1.15×10^-1^ | 5.83×10^-3^ | - |
|  |  |  | PB | 7.70 | n.s. | n.s. | n.s. | - | n.s. | n.s. | n.s. | - |
| Lobule  Crus II | L | Quadratic | ES | 5.35 | n.s. | 4.35×10^-1^ | -1.96×10^-2^ | - | n.s. | n.s. | n.s. | - |
|  |  |  | CP | 4.93 | n.s. | 5.51×10^-1^ | -2.64×10^-2^ | - | n.s. | n.s. | n.s. | - |
|  |  |  | HI | 4.81 | n.s. | 5.53×10^-1^ | -2.54×10^-2^ | - | n.s. | n.s. | n.s. | - |
|  |  |  | PP | 4.72 | n.s. | 5.73×10^-1^ | -2.70×10^-2^ | - | n.s. | n.s. | n.s. | - |
|  |  |  | PB | 7.75 | n.s. | n.s. | n.s. | - | n.s. | n.s. | n.s. | - |
|  | R | Quadratic | ES | 5.34 | n.s. | 4.95×10^-1^ | -2.34×10^-2^ | - | n.s. | n.s. | n.s. | - |
|  |  |  | CP | 5.29 | n.s. | 5.26×10^-1^ | -2.58×10^-2^ | - | n.s. | n.s. | n.s. | - |
|  |  |  | HI | 5.18 | n.s. | 5.50×10^-1^ | -2.69×10^-2^ | - | n.s. | n.s. | n.s. | - |
|  |  |  | PP | 5.40 | n.s. | 4.64×10^-1^ | -2.10×10^-2^ | - | n.s. | n.s. | n.s. | - |
|  |  |  | PB | 5.61 | n.s. | n.s. | n.s. | - | n.s. | n.s. | n.s. | - |
| Lobule ⅦB | L | None | ES | 4.21 | n.s. | n.s. | - | - | n.s. | - | - | - |
|  |  |  | CP | 4.13 | n.s. | n.s. | - | - | n.s. | - | - | - |
|  |  |  | HI | 4.24 | n.s. | n.s. | - | - | n.s. | - | - | - |
|  |  |  | PP | 4.16 | n.s. | n.s. | - | - | n.s. | - | - | - |
|  |  |  | PB | 4.17 | n.s. | n.s. | - | - | n.s. | - | - | - |
|  | R | None | ES | 4.25 | n.s. | n.s. | - | - | n.s. | - | - | - |
|  |  |  | CP | 4.22 | n.s. | n.s. | - | - | n.s. | - | - | - |
|  |  |  | HI | 4.25 | n.s. | n.s. | - | - | n.s. | - | - | - |
|  |  |  | PP | 4.20 | n.s. | n.s. | - | - | n.s. | - | - | - |
|  |  |  | PB | 4.14 | n.s. | n.s. | - | - | n.s. | - | - | - |
| Lobule ⅧA | L | Linear | ES | 5.04 | n.s. | 4.18×10^-2^ | - | - | n.s. | n.s. | - | - |
|  |  |  | CP | 4.89 | n.s. | 5.24×10^-2^ | - | - | n.s. | n.s. | - | - |
|  |  |  | HI | 5.01 | n.s. | 4.48×10^-2^ | - | - | n.s. | n.s. | - | - |
|  |  |  | PP | 5.02 | n.s. | 4.22×10^-2^ | - | - | n.s. | n.s. | - | - |
|  |  |  | PB | 5.50 | n.s. | n.s. | - | - | n.s. | n.s. | - | - |
|  | R | Linear | ES | 4.82 | n.s. | 5.48×10^-2^ | - | - | n.s. | n.s. | - | - |
|  |  |  | CP | 4.80 | n.s. | 5.52×10^-2^ | - | - | n.s. | n.s. | - | - |
|  |  |  | HI | 4.94 | n.s. | 4.22×10^-2^ | - | - | n.s. | n.s. | - | - |
|  |  |  | PP | 4.94 | n.s. | 4.00×10^-2^ | - | - | n.s. | n.s. | - | - |
|  |  |  | PB | 5.30 | n.s. | n.s. | - | - | n.s. | n.s. | - | - |
| Lobule ⅧB | L | Linear | ES | 2.98 | n.s. | 4.88×10^-2^ | - | - | n.s. | n.s. | - | - |
|  |  |  | CP | 3.07 | n.s. | 3.78×10^-2^ | - | - | n.s. | n.s. | - | - |
|  |  |  | HI | 3.01 | n.s. | 4.38×10^-2^ | - | - | n.s. | n.s. | - | - |
|  |  |  | PP | 3.07 | n.s. | 4.16×10^-2^ | - | - | n.s. | n.s. | - | - |
|  |  |  | PB | 3.36 | n.s. | n.s. | - | - | n.s. | n.s. | - | - |
|  | R | Linear | ES | 3.11 | n.s. | 3.41×10^-2^ | - | - | n.s. | n.s. | - | - |
|  |  |  | CP | 3.15 | n.s. | 3.03×10^-2^ | - | - | n.s. | n.s. | - | - |
|  |  |  | HI | 3.20 | n.s. | n.s. | - | - | n.s. | n.s. | - | - |
|  |  |  | PP | 3.23 | n.s. | n.s. | - | - | n.s. | n.s. | - | - |
|  |  |  | PB | 3.37 | n.s. | n.s. | - | - | n.s. | n.s. | - | - |
| Lobule Ⅸ | L | Quadratic | ES | 2.50 | n.s. | n.s. | n.s. | - | n.s. | n.s. | n.s. | - |
|  |  |  | CP | 2.34 | n.s. | 1.27×10^-1^ | n.s. | - | n.s. | n.s. | n.s. | - |
|  |  |  | HI | 2.33 | n.s. | n.s. | n.s. | - | n.s. | n.s. | n.s. | - |
|  |  |  | PP | 2.13 | n.s. | 1.69×10^-1^ | -7.64×10^-3^ | - | n.s. | n.s. | n.s. | - |
|  |  |  | PB | 2.33 | n.s. | n.s. | n.s. | - | n.s. | n.s. | n.s. | - |
|  | R | Linear | ES | 3.13 | n.s. | n.s. | - | - | n.s. | n.s. | - | - |
|  |  |  | CP | 3.14 | n.s. | n.s. | - | - | n.s. | n.s. | - | - |
|  |  |  | HI | 3.09 | n.s. | n.s. | - | - | n.s. | n.s. | - | - |
|  |  |  | PP | 3.13 | n.s. | n.s. | - | - | n.s. | n.s. | - | - |
|  |  |  | PB | 3.03 | n.s. | n.s. | - | - | n.s. | n.s. | - | - |
| Lobule Ⅹ | L | Linear | ES | 4.83×10^-1^ | n.s. | 6.67×10^-3^ | - | - | n.s. | n.s. | - | - |
|  |  |  | CP | 4.90×10^-1^ | n.s. | 5.59×10^-3^ | - | - | n.s. | n.s. | - | - |
|  |  |  | HI | 4.64×10^-1^ | n.s. | 8.37×10^-3^ | - | - | n.s. | n.s. | - | - |
|  |  |  | PP | 4.92×10^-1^ | n.s. | 6.01×10^-3^ | - | - | n.s. | n.s. | - | - |
|  |  |  | PB | 4.21×10^-1^ | n.s. | 1.33×10^-2^ | - | - | n.s. | n.s. | - | - |
|  | R | Linear | ES | 4.88×10^-1^ | n.s. | 5.15×10^-3^ | - | - | n.s. | n.s. | - | - |
|  |  |  | CP | 4.74×10^-1^ | n.s. | 6.24×10^-3^ | - | - | n.s. | n.s. | - | - |
|  |  |  | HI | 4.65×10^-1^ | n.s. | 6.93×10^-3^ | - | - | n.s. | n.s. | - | - |
|  |  |  | PP | 4.91×10^-1^ | n.s. | 5.00×10^-3^ | - | - | n.s. | n.s. | - | - |
|  |  |  | PB | 5.07×10^-1^ | n.s. | n.s. | - | - | n.s. | n.s. | - | - |

The level of significance is 0.0066 after multiple comparisons correction, – = not applicable; n.s. = non-significant, HS = Hemisphere, L = left, R = right, EBP = emotional and behavioural problems, ES = Emotional Symptoms, CP =Conduct Problems , HI = Hyperactivity/Inattention, PP = Peer Relationship Problems, PB = Prosocial Behaviors

Table S8 Model parameters for fixed effects when including level of emotional and behavioural problems in the best fitting models for whole cerebellum and cerebeller subregion cortical thicknesses in girls.

| Cortical thickess | HS | Best fitting model | EBP Scales | Intercept | Site | Age coefficient  β2 | Age^2^ coefficient  β3 | Age^3^ coefficient  β3 | EBP | Age×EBP coefficient  β5 | Age^2^×EBP coefficient  β6 | Age^3^×EBP coefficient  β6 |
| --- | --- | --- | --- | --- | --- | --- | --- | --- | --- | --- | --- | --- |
| Cerebellum | L | Linear | ES | 4.79 | n.s. | 1.14×10^-2^ | - | - | n.s. | n.s. | - | - |
|  |  |  | CP | 4.81 | n.s. | 9.86×10^-3^ | - | - | n.s. | n.s. | - | - |
|  |  |  | HI | 4.76 | n.s. | 1.53×10^-2^ | - | - | n.s. | n.s. | - | - |
|  |  |  | PP | 4.79 | n.s. | 1.12×10^-2^ | - | - | n.s. | n.s. | - | - |
|  |  |  | PB | 4.86 | n.s. | n.s. | - | - | n.s. | n.s. | - | - |
|  | R | None | ES | 4.86 | n.s. | n.s. | - | - | n.s. | - | - | - |
|  |  |  | CP | 4.87 | n.s. | n.s. | - | - | n.s. | - | - | - |
|  |  |  | HI | 4.87 | n.s. | n.s. | - | - | n.s. | - | - | - |
|  |  |  | PP | 4.86 | n.s. | n.s. | - | - | n.s. | - | - | - |
|  |  |  | PB | 4.85 | n.s. | n.s. | - | - | n.s. | - | - | - |
| Lobule Ⅰ-Ⅱ | L | None | ES | 2.45 | n.s. | n.s. | - | - | n.s. | - | - | - |
|  |  |  | CP | 2.39 | n.s. | n.s. | - | - | n.s. | - | - | - |
|  |  |  | HI | 2.49 | n.s. | n.s. | - | - | n.s. | - | - | - |
|  |  |  | PP | 2.38 | n.s. | n.s. | - | - | n.s. | - | - | - |
|  |  |  | PB | 2.41 | n.s. | n.s. | - | - | n.s. | - | - | - |
|  | R | None | ES | 2.28 | n.s. | n.s. | - | - | n.s. | - | - | - |
|  |  |  | CP | 2.25 | n.s. | n.s. | - | - | n.s. | - | - | - |
|  |  |  | HI | 2.35 | n.s. | n.s. | - | - | n.s. | - | - | - |
|  |  |  | PP | 2.23 | n.s. | n.s. | - | - | n.s. | - | - | - |
|  |  |  | PB | 2.27 | n.s. | n.s. | - | - | n.s. | - | - | - |
| Lobule Ⅲ | L | None | ES | 3.66 | n.s. | n.s. | - | - | n.s. | - | - | - |
|  |  |  | CP | 3.64 | n.s. | n.s. | - | - | n.s. | - | - | - |
|  |  |  | HI | 3.66 | n.s. | n.s. | - | - | n.s. | - | - | - |
|  |  |  | PP | 3.65 | n.s. | n.s. | - | - | n.s. | - | - | - |
|  |  |  | PB | 3.71 | n.s. | n.s. | - | - | n.s. | - | - | - |
|  | R | None | ES | 3.51 | n.s. | n.s. | - | - | n.s. | - | - | - |
|  |  |  | CP | 3.50 | n.s. | n.s. | - | - | n.s. | - | - | - |
|  |  |  | HI | 3.54 | n.s. | n.s. | - | - | n.s. | - | - | - |
|  |  |  | PP | 3.52 | n.s. | n.s. | - | - | n.s. | - | - | - |
|  |  |  | PB | 3.60 | n.s. | n.s. | - | - | n.s. | - | - | - |
| Lobule Ⅳ | L | Linear | ES | 5.11 | n.s. | 8.94×10^-3^ | - | - | n.s. | n.s. | - | - |
|  |  |  | CP | 5.11 | n.s. | 8.73×10^-3^ | - | - | n.s. | n.s. | - | - |
|  |  |  | HI | 5.11 | n.s. | n.s. | - | - | n.s. | n.s. | - | - |
|  |  |  | PP | 5.12 | n.s. | n.s. | - | - | n.s. | n.s. | - | - |
|  |  |  | PB | 5.28 | n.s. | n.s. | - | - | n.s. | n.s. | - | - |
|  | R | Linear | ES | 5.06 | n.s. | n.s. | - | - | n.s. | n.s. | - | - |
|  |  |  | CP | 5.06 | n.s. | n.s. | - | - | n.s. | n.s. | - | - |
|  |  |  | HI | 5.03 | n.s. | n.s. | - | - | n.s. | n.s. | - | - |
|  |  |  | PP | 5.08 | n.s. | n.s. | - | - | n.s. | n.s. | - | - |
|  |  |  | PB | 5.10 | n.s. | n.s. | - | - | n.s. | n.s. | - | - |
| Lobule Ⅴ | L | Linear | ES | 5.12 | n.s. | n.s. | - | - | n.s. | n.s. | - | - |
|  |  |  | CP | 5.12 | n.s. | n.s. | - | - | n.s. | n.s. | - | - |
|  |  |  | HI | 5.09 | n.s. | 1.06×10^-2^ | - | - | n.s. | n.s. | - | - |
|  |  |  | PP | 5.09 | n.s. | 9.81×10^-3^ | - | - | n.s. | n.s. | - | - |
|  |  |  | PB | 5.14 | n.s. | n.s. | - | - | n.s. | n.s. | - | - |
|  | R | Linear | ES | 4.90 | n.s. | 1.33×10^-2^ | - | - | n.s. | n.s. | - | - |
|  |  |  | CP | 4.90 | n.s. | 1.40×10^-2^ | - | - | n.s. | n.s. | - | - |
|  |  |  | HI | 4.84 | n.s. | 1.87×10^-2^ | - | - | n.s. | n.s. | - | - |
|  |  |  | PP | 4.86 | n.s. | 1.76×10^-2^ | - | - | n.s. | n.s. | - | - |
|  |  |  | PB | 4.86 | n.s. | n.s. | - | - | n.s. | n.s. | - | - |
| Lobule Ⅵ | L | None | ES | 5.18 | n.s. | n.s. | - | - | n.s. | - | - | - |
|  |  |  | CP | 5.18 | n.s. | n.s. | - | - | n.s. | - | - | - |
|  |  |  | HI | 5.18 | n.s. | n.s. | - | - | n.s. | - | - | - |
|  |  |  | PP | 5.17 | n.s. | n.s. | - | - | n.s. | - | - | - |
|  |  |  | PB | 5.17 | n.s. | n.s. | - | - | n.s. | - | - | - |
|  | R | None | ES | 5.14 | n.s. | n.s. | - | - | n.s. | - | - | - |
|  |  |  | CP | 5.15 | n.s. | n.s. | - | - | n.s. | - | - | - |
|  |  |  | HI | 5.16 | n.s. | n.s. | - | - | n.s. | - | - | - |
|  |  |  | PP | 5.15 | n.s. | n.s. | - | - | n.s. | - | - | - |
|  |  |  | PB | 5.13 | n.s. | n.s. | - | - | n.s. | - | - | - |
| Lobule  Crus I | L | Linear | ES | 4.61 | n.s. | 1.83×10^-2^ | - | - | n.s. | n.s. | - | - |
|  |  |  | CP | 4.65 | n.s. | 1.56×10^-2^ | - | - | n.s. | n.s. | - | - |
|  |  |  | HI | 4.56 | n.s. | 2.37×10^-2^ | - | - | n.s. | n.s. | - | - |
|  |  |  | PP | 4.60 | n.s. | 1.85×10^-2^ | - | - | n.s. | n.s. | - | - |
|  |  |  | PB | 4.68 | n.s. | n.s. | - | - | n.s. | n.s. | - | - |
|  | R | None | ES | 4.80 | n.s. | n.s. | - | - | n.s. | - | - | - |
|  |  |  | CP | 4.80 | n.s. | n.s. | - | - | n.s. | - | - | - |
|  |  |  | HI | 4.80 | n.s. | n.s. | - | - | n.s. | - | - | - |
|  |  |  | PP | 4.78 | n.s. | n.s. | - | - | n.s. | - | - | - |
|  |  |  | PB | 4.77 | n.s. | n.s. | - | - | n.s. | - | - | - |
| Lobule  Crus II | L | Linear | ES | 4.53 | n.s. | 2.46×10^-2^ | - | - | n.s. | n.s. | - | - |
|  |  |  | CP | 4.59 | n.s. | 2.07×10^-2^ | - | - | n.s. | n.s. | - | - |
|  |  |  | HI | 4.50 | n.s. | 2.94×10^-2^ | - | - | n.s. | n.s. | - | - |
|  |  |  | PP | 4.59 | n.s. | 1.87×10^-2^ | - | - | n.s. | n.s. | - | - |
|  |  |  | PB | 4.53 | n.s. | n.s. | - | - | n.s. | n.s. | - | - |
|  | R | Linear | ES | 4.85 | n.s. | n.s. | - | - | n.s. | n.s. | - | - |
|  |  |  | CP | 4.80 | n.s. | n.s. | - | - | n.s. | n.s. | - | - |
|  |  |  | HI | 4.77 | n.s. | n.s. | - | - | n.s. | n.s. | - | - |
|  |  |  | PP | 4.80 | n.s. | n.s. | - | - | n.s. | n.s. | - | - |
|  |  |  | PB | 4.63 | n.s. | n.s. | - | - | n.s. | n.s. | - | - |
| Lobule ⅦB | L | None | ES | 4.95 | n.s. | n.s. | - | - | n.s. | - | - | - |
|  |  |  | CP | 4.95 | n.s. | n.s. | - | - | n.s. | - | - | - |
|  |  |  | HI | 4.95 | n.s. | n.s. | - | - | n.s. | - | - | - |
|  |  |  | PP | 4.94 | n.s. | n.s. | - | - | n.s. | - | - | - |
|  |  |  | PB | 4.92 | n.s. | n.s. | - | - | n.s. | - | - | - |
|  | R | None | ES | 4.95 | n.s. | n.s. | - | - | n.s. | - | - | - |
|  |  |  | CP | 4.96 | n.s. | n.s. | - | - | n.s. | - | - | - |
|  |  |  | HI | 4.98 | n.s. | n.s. | - | - | n.s. | - | - | - |
|  |  |  | PP | 4.94 | n.s. | n.s. | - | - | n.s. | - | - | - |
|  |  |  | PB | 4.93 | n.s. | n.s. | - | - | n.s. | - | - | - |
| Lobule ⅧA | L | None | ES | 4.89 | n.s. | n.s. | - | - | n.s. | - | - | - |
|  |  |  | CP | 4.88 | n.s. | n.s. | - | - | n.s. | - | - | - |
|  |  |  | HI | 4.88 | n.s. | n.s. | - | - | n.s. | - | - | - |
|  |  |  | PP | 4.88 | n.s. | n.s. | - | - | n.s. | - | - | - |
|  |  |  | PB | 4.88 | n.s. | n.s. | - | - | n.s. | - | - | - |
|  | R | None | ES | 4.80 | n.s. | n.s. | - | - | n.s. | - | - | - |
|  |  |  | CP | 4.80 | n.s. | n.s. | - | - | n.s. | - | - | - |
|  |  |  | HI | 4.81 | n.s. | n.s. | - | - | n.s. | - | - | - |
|  |  |  | PP | 4.79 | n.s. | n.s. | - | - | n.s. | - | - | - |
|  |  |  | PB | 4.84 | n.s. | n.s. | - | - | n.s. | - | - | - |
| Lobule ⅧB | L | Linear | ES | 4.75 | n.s. | 2.18×10^-2^ | - | - | n.s. | n.s. | - | - |
|  |  |  | CP | 4.79 | n.s. | 1.74×10^-2^ | - | - | n.s. | n.s. | - | - |
|  |  |  | HI | 4.72 | n.s. | 2.38×10^-2^ | - | - | n.s. | n.s. | - | - |
|  |  |  | PP | 4.76 | n.s. | 2.22×10^-2^ | - | - | n.s. | n.s. | - | - |
|  |  |  | PB | 4.94 | n.s. | n.s. | - | - | n.s. | n.s. | - | - |
|  | R | None | ES | 4.89 | n.s. | n.s. | - | - | n.s. | - | - | - |
|  |  |  | CP | 4.88 | n.s. | n.s. | - | - | n.s. | - | - | - |
|  |  |  | HI | 4.89 | n.s. | n.s. | - | - | n.s. | - | - | - |
|  |  |  | PP | 4.88 | n.s. | n.s. | - | - | n.s. | - | - | - |
|  |  |  | PB | 4.90 | n.s. | n.s. | - | - | n.s. | - | - | - |
| Lobule Ⅸ | L | None | ES | 4.55 | n.s. | n.s. | - | - | n.s. | - | - | - |
|  |  |  | CP | 4.53 | n.s. | n.s. | - | - | n.s. | - | - | - |
|  |  |  | HI | 4.50 | n.s. | n.s. | - | - | n.s. | - | - | - |
|  |  |  | PP | 4.55 | n.s. | n.s. | - | - | n.s. | - | - | - |
|  |  |  | PB | 4.53 | n.s. | n.s. | - | - | n.s. | - | - | - |
|  | R | None | ES | 4.77 | n.s. | -1.62×10^-2^ | - | - | n.s. | - | - | - |
|  |  |  | CP | 4.77 | n.s. | -1.53×10^-2^ | - | - | n.s. | - | - | - |
|  |  |  | HI | 4.74 | n.s. | n.s. | - | - | n.s. | - | - | - |
|  |  |  | PP | 4.75 | n.s. | n.s. | - | - | n.s. | - | - | - |
|  |  |  | PB | 4.79 | n.s. | -1.49×10^-2^ | - | - | n.s. | - | - | - |
| Lobule Ⅹ | L | Linear | ES | 3.30 | n.s. | 3.90×10^-2^ | - | - | n.s. | n.s. | - | - |
|  |  |  | CP | 3.38 | n.s. | n.s. | - | - | n.s. | n.s. | - | - |
|  |  |  | HI | 3.46 | n.s. | n.s. | - | - | n.s. | n.s. | - | - |
|  |  |  | PP | 3.32 | n.s. | 3.99×10^-2^ | - | - | n.s. | n.s. | - | - |
|  |  |  | PB | 3.21 | n.s. | n.s. | - | - | n.s. | n.s. | - | - |
|  | R | Linear | ES | 2.90 | n.s. | n.s. | - | - | n.s. | n.s. | - | - |
|  |  |  | CP | 2.77 | n.s. | n.s. | - | - | n.s. | n.s. | - | - |
|  |  |  | HI | 2.82 | n.s. | n.s. | - | - | n.s. | n.s. | - | - |
|  |  |  | PP | 2.78 | n.s. | n.s. | - | - | n.s. | n.s. | - | - |
|  |  |  | PB | 3.30 | n.s. | n.s. | - | - | n.s. | n.s. | - | - |

The level of significance is 0.0065 after multiple comparisons correction, – = not applicable; n.s. = non-significant, HS = Hemisphere, L = left, R = right, EBP = emotional and behavioural problems, ES = Emotional Symptoms, CP =Conduct Problems , HI = Hyperactivity/Inattention, PP = Peer Relationship Problems, PB = Prosocial Behaviors

Table S9 Model parameters for fixed effects when including level of emotional and behavioural problems in the best fitting models for whole cerebellum and cerebeller subregion surface areas in girls.

| Surface area | HS | Best fitting model | EBP Scales | Intercept | Site | Age coefficient  β2 | Age^2^ coefficient  β3 | Age^3^ coefficient  β3 | EBP | Age×EBP coefficient  β5 | Age^2^×EBP coefficient  β6 | Age^3^×EBP coefficient  β6 |
| --- | --- | --- | --- | --- | --- | --- | --- | --- | --- | --- | --- | --- |
| Cerebellum | L | Quadratic | ES | 8.47 | n.s. | 3.20×10^-1^ | -1.45×10^-2^ | - | n.s. | n.s. | n.s. | - |
|  |  |  | CP | 8.49 | n.s. | 3.16×10^-1^ | -1.45×10^-2^ | - | n.s. | n.s. | n.s. | - |
|  |  |  | HI | 8.70 | n.s. | 2.87×10^-1^ | -1.35×10^-2^ | - | n.s. | n.s. | n.s. | - |
|  |  |  | PP | 8.39 | n.s. | 3.40×10^-1^ | -1.56×10^-2^ | - | n.s. | n.s. | n.s. | - |
|  |  |  | PB | 8.45 | n.s. | n.s. | n.s. | - | n.s. | n.s. | n.s. | - |
|  | R | Cubic | ES | 1.11×10 | n.s. | n.s. | n.s. | -2.79×10^-3^ | n.s. | n.s. | n.s. | n.s. |
|  |  |  | CP | 1.21×10 | n.s. | n.s. | n.s. | -3.56×10^-3^ | n.s. | n.s. | n.s. | n.s. |
|  |  |  | HI | 1.21×10 | n.s. | n.s. | n.s. | -3.33×10^-3^ | n.s. | n.s. | n.s. | n.s. |
|  |  |  | PP | 1.09×10 | n.s. | n.s. | n.s. | n.s. | n.s. | n.s. | n.s. | n.s. |
|  |  |  | PB | 1.40×10 | n.s. | n.s. | n.s. | n.s. | n.s. | n.s. | n.s. | n.s. |
| Lobule Ⅰ-Ⅱ | L | None | ES | 1.09×10^-2^ | n.s. | n.s. | - | - | n.s. | - | - | - |
|  |  |  | CP | 1.15×10^-2^ | n.s. | n.s. | - | - | n.s. | - | - | - |
|  |  |  | HI | 1.11×10^-2^ | n.s. | n.s. | - | - | n.s. | - | - | - |
|  |  |  | PP | 1.12×10^-2^ | n.s. | n.s. | - | - | n.s. | - | - | - |
|  |  |  | PB | 1.24×10^-2^ | n.s. | n.s. | - | - | n.s. | - | - | - |
|  | R | None | ES | 1.72×10^-2^ | n.s. | n.s. | - | - | n.s. | - | - | - |
|  |  |  | CP | 1.73×10^-2^ | n.s. | n.s. | - | - | n.s. | - | - | - |
|  |  |  | HI | 1.69×10^-2^ | n.s. | n.s. | - | - | n.s. | - | - | - |
|  |  |  | PP | 1.73×10^-2^ | n.s. | n.s. | - | - | n.s. | - | - | - |
|  |  |  | PB | 1.86×10^-2^ | n.s. | n.s. | - | - | n.s. | - | - | - |
| Lobule Ⅲ | L | Linear | ES | 1.35×10^-1^ | n.s. | 9.53×10^-4^ | - | - | n.s. | n.s. | - | - |
|  |  |  | CP | 1.36×10^-1^ | n.s. | n.s. | - | - | n.s. | n.s. | - | - |
|  |  |  | HI | 1.38×10^-1^ | n.s. | n.s. | - | - | n.s. | n.s. | - | - |
|  |  |  | PP | 1.35×10^-1^ | n.s. | n.s. | - | - | n.s. | n.s. | - | - |
|  |  |  | PB | 1.43×10^-1^ | n.s. | n.s. | - | - | n.s. | n.s. | - | - |
|  | R | None | ES | 1.40×10^-1^ | n.s. | n.s. | - | - | n.s. | - | - | - |
|  |  |  | CP | 1.37×10^-1^ | n.s. | 8.70×10^-4^ | - | - | n.s. | - | - | - |
|  |  |  | HI | 1.40×10^-1^ | n.s. | n.s. | - | - | n.s. | - | - | - |
|  |  |  | PP | 1.39×10^-1^ | n.s. | n.s. | - | - | n.s. | - | - | - |
|  |  |  | PB | 1.41×10^-1^ | n.s. | n.s. | - | - | n.s. | - | - | - |
| Lobule Ⅳ | L | Quadratic | ES | 3.56×10^-1^ | n.s. | n.s. | n.s. | - | n.s. | n.s. | n.s. | - |
|  |  |  | CP | 3.31×10^-1^ | n.s. | 1.71×10^-2^ | -8.04×10^-4^ | - | n.s. | n.s. | n.s. | - |
|  |  |  | HI | 3.29×10^-1^ | n.s. | n.s. | n.s. | - | n.s. | n.s. | n.s. | - |
|  |  |  | PP | 3.53×10^-1^ | n.s. | n.s. | n.s. | - | n.s. | n.s. | n.s. | - |
|  |  |  | PB | 3.56×10^-1^ | n.s. | n.s. | n.s. | - | n.s. | n.s. | n.s. | - |
|  | R | Quadratic | ES | 3.35×10^-1^ | n.s. | 1.59×10^-2^ | -7.28×10^-4^ | - | n.s. | n.s. | n.s. | - |
|  |  |  | CP | 2.99×10^-1^ | n.s. | 2.33×10^-2^ | -1.09×10^-3^ | - | n.s. | n.s. | n.s. | - |
|  |  |  | HI | 3.32×10^-1^ | n.s. | 1.81×10^-2^ | -8.71×10^-4^ | - | n.s. | n.s. | n.s. | - |
|  |  |  | PP | 3.27×10^-1^ | n.s. | 1.73×10^-2^ | -7.71×10^-4^ | - | n.s. | n.s. | n.s. | - |
|  |  |  | PB | 4.30×10^-1^ | n.s. | n.s. | n.s. | - | n.s. | n.s. | n.s. | - |
| Lobule Ⅴ | L | Linear | ES | 6.79×10^-1^ | n.s. | 4.27×10^-3^ | - | - | n.s. | n.s. | - | - |
|  |  |  | CP | 6.65×10^-1^ | n.s. | 5.44×10^-3^ | - | - | n.s. | n.s. | - | - |
|  |  |  | HI | 6.98×10^-1^ | n.s. | n.s. | - | - | -8.87×10^-3^ | 9.21×10^-4^ | - | - |
|  |  |  | PP | 6.92×10^-1^ | n.s. | 3.18×10^-3^ | - | - | -1.32×10^-2^ | n.s. | - | - |
|  |  |  | PB | 6.52×10^-1^ | n.s. | 6.94×10^-3^ | - | - | n.s. | n.s. | - | - |
|  | R | Quadratic | ES | 5.58×10^-1^ | n.s. | 2.08×10^-2^ | n.s. | - | n.s. | n.s. | n.s. | - |
|  |  |  | CP | 5.36×10^-1^ | n.s. | 2.56×10^-2^ | n.s. | - | n.s. | n.s. | n.s. | - |
|  |  |  | HI | 5.79×10^-1^ | n.s. | n.s. | n.s. | - | n.s. | n.s. | n.s. | - |
|  |  |  | PP | 5.86×10^-1^ | n.s. | n.s. | n.s. | - | n.s. | n.s. | n.s. | - |
|  |  |  | PB | 6.39×10^-1^ | n.s. | n.s. | n.s. | - | n.s. | n.s. | n.s. | - |
| Lobule Ⅵ | L | Quadratic | ES | 1.20 | n.s. | 6.89×10^-2^ | -3.10×10^-3^ | - | n.s. | n.s. | n.s. | - |
|  |  |  | CP | 1.23 | n.s. | 6.39×10^-2^ | -2.96×10^-3^ | - | n.s. | n.s. | n.s. | - |
|  |  |  | HI | 1.18 | n.s. | 7.30×10^-2^ | -3.34×10^-3^ | - | n.s. | n.s. | n.s. | - |
|  |  |  | PP | 1.18 | n.s. | 7.37×10^-2^ | -3.41×10^-3^ | - | n.s. | n.s. | n.s. | - |
|  |  |  | PB | 1.35 | n.s. | n.s. | n.s. | - | n.s. | n.s. | n.s. | - |
|  | R | Quadratic | ES | 1.17 | n.s. | 6.37×10^-2^ | -2.51×10^-3^ | - | n.s. | n.s. | n.s. | - |
|  |  |  | CP | 1.21 | n.s. | 5.91×10^-2^ | -2.44×10^-3^ | - | n.s. | n.s. | n.s. | - |
|  |  |  | HI | 1.25 | n.s. | 5.08×10^-2^ | -1.97×10^-3^ | - | n.s. | n.s. | n.s. | - |
|  |  |  | PP | 1.22 | n.s. | 5.59×10^-2^ | -2.24×10^-3^ | - | n.s. | n.s. | n.s. | - |
|  |  |  | PB | 1.36 | n.s. | n.s. | n.s. | - | n.s. | n.s. | n.s. | - |
| Lobule  Crus I | L | Quadratic | ES | 1.79 | n.s. | 8.86×10^-2^ | -4.49×10^-3^ | - | n.s. | n.s. | n.s. | - |
|  |  |  | CP | 1.86 | n.s. | n.s. | n.s. | - | n.s. | n.s. | n.s. | - |
|  |  |  | HI | 1.90 | n.s. | n.s. | n.s. | - | n.s. | n.s. | n.s. | - |
|  |  |  | PP | 1.82 | n.s. | 8.23×10^-2^ | -4.12×10^-3^ | - | n.s. | n.s. | n.s. | - |
|  |  |  | PB | 1.80 | n.s. | n.s. | n.s. | - | n.s. | n.s. | n.s. | - |
|  | R | Cubic | ES | 2.32 | n.s. | n.s. | n.s. | n.s. | n.s. | n.s. | n.s. | n.s. |
|  |  |  | CP | 2.17 | n.s. | n.s. | n.s. | n.s. | n.s. | n.s. | n.s. | n.s. |
|  |  |  | HI | 2.46 | n.s. | n.s. | n.s. | n.s. | n.s. | n.s. | n.s. | n.s. |
|  |  |  | PP | 2.03 | n.s. | n.s. | n.s. | n.s. | n.s. | n.s. | n.s. | n.s. |
|  |  |  | PB | 6.19 | n.s. | -1.25 | 1.26×10^-1^ | -4.12×10^-3^ | -4.61×10^-1^ | n.s. | n.s. | n.s. |
| Lobule  Crus II | L | Quadratic | ES | 1.35 | n.s. | n.s. | n.s. | - | n.s. | n.s. | n.s. | - |
|  |  |  | CP | 1.30 | n.s. | n.s. | -3.47×10^-3^ | - | n.s. | n.s. | n.s. | - |
|  |  |  | HI | 1.19 | n.s. | 9.01×10^-2^ | -4.63×10^-3^ | - | n.s. | n.s. | n.s. | - |
|  |  |  | PP | 1.22 | n.s. | 7.96×10^-2^ | -3.96×10^-3^ | - | n.s. | n.s. | n.s. | - |
|  |  |  | PB | 1.41 | n.s. | n.s. | n.s. | - | n.s. | n.s. | n.s. | - |
|  | R | Quadratic | ES | 1.20 | n.s. | 8.05×10^-2^ | -3.87×10^-3^ | - | n.s. | n.s. | n.s. | - |
|  |  |  | CP | 1.24 | n.s. | n.s. | -3.96×10^-3^ | - | n.s. | n.s. | n.s. | - |
|  |  |  | HI | 1.22 | n.s. | n.s. | -4.49×10^-3^ | - | n.s. | n.s. | n.s. | - |
|  |  |  | PP | 1.24 | n.s. | n.s. | n.s. | - | n.s. | n.s. | n.s. | - |
|  |  |  | PB | 1.21 | n.s. | n.s. | n.s. | - | n.s. | n.s. | n.s. | - |
| Lobule ⅦB | L | None | ES | 8.53×10^-1^ | n.s. | n.s. | - | - | n.s. | - | - | - |
|  |  |  | CP | 8.36×10^-1^ | n.s. | n.s. | - | - | n.s. | - | - | - |
|  |  |  | HI | 8.57×10^-1^ | n.s. | n.s. | - | - | n.s. | - | - | - |
|  |  |  | PP | 8.43×10^-1^ | n.s. | n.s. | - | - | n.s. | - | - | - |
|  |  |  | PB | 8.48×10^-1^ | n.s. | n.s. | - | - | n.s. | - | - | - |
|  | R | None | ES | 8.58×10^-1^ | n.s. | n.s. | - | - | n.s. | - | - | - |
|  |  |  | CP | 8.51×10^-1^ | n.s. | n.s. | - | - | n.s. | - | - | - |
|  |  |  | HI | 8.53×10^-1^ | n.s. | n.s. | - | - | n.s. | - | - | - |
|  |  |  | PP | 8.53×10^-1^ | n.s. | n.s. | - | - | n.s. | - | - | - |
|  |  |  | PB | 8.38×10^-1^ | n.s. | n.s. | - | - | n.s. | - | - | - |
| Lobule ⅧA | L | Linear | ES | 1.05 | n.s. | n.s. | - | - | n.s. | n.s. | - | - |
|  |  |  | CP | 1.01 | n.s. | 8.21×10^-3^ | - | - | n.s. | n.s. | - | - |
|  |  |  | HI | 1.05 | n.s. | n.s. | - | - | n.s. | n.s. | - | - |
|  |  |  | PP | 1.04 | n.s. | n.s. | - | - | n.s. | n.s. | - | - |
|  |  |  | PB | 1.11 | n.s. | n.s. | - | - | n.s. | n.s. | - | - |
|  | R | Linear | ES | 1.01 | n.s. | 9.84×10^-3^ | - | - | n.s. | n.s. | - | - |
|  |  |  | CP | 1.01 | n.s. | 9.88×10^-3^ | - | - | n.s. | n.s. | - | - |
|  |  |  | HI | 1.04 | n.s. | n.s. | - | - | n.s. | n.s. | - | - |
|  |  |  | PP | 1.05 | n.s. | n.s. | - | - | n.s. | n.s. | - | - |
|  |  |  | PB | 1.09 | n.s. | n.s. | - | - | n.s. | n.s. | - | - |
| Lobule ⅧB | L | Linear | ES | 6.42×10^-1^ | n.s. | 5.50×10^-3^ | - | - | n.s. | n.s. | - | - |
|  |  |  | CP | 6.53×10^-1^ | n.s. | n.s. | - | - | n.s. | n.s. | - | - |
|  |  |  | HI | 6.58×10^-1^ | n.s. | n.s. | - | - | n.s. | n.s. | - | - |
|  |  |  | PP | 6.49×10^-1^ | n.s. | 4.84×10^-3^ | - | - | n.s. | n.s. | - | - |
|  |  |  | PB | 6.79×10^-1^ | n.s. | n.s. | - | - | n.s. | n.s. | - | - |
|  | R | Linear | ES | 6.45×10^-1^ | n.s. | 6.15×10^-3^ | - | - | n.s. | n.s. | - | - |
|  |  |  | CP | 6.51×10^-1^ | n.s. | 5.68×10^-3^ | - | - | n.s. | n.s. | - | - |
|  |  |  | HI | 6.64×10^-1^ | n.s. | n.s. | - | - | n.s. | n.s. | - | - |
|  |  |  | PP | 6.72×10^-1^ | n.s. | n.s. | - | - | n.s. | n.s. | - | - |
|  |  |  | PB | 6.94×10^-1^ | n.s. | n.s. | - | - | n.s. | n.s. | - | - |
| Lobule Ⅸ | L | Quadratic | ES | 5.11×10^-1^ | n.s. | 3.02×10^-2^ | -1.33×10^-3^ | - | n.s. | n.s. | n.s. | - |
|  |  |  | CP | 4.96×10^-1^ | n.s. | 3.43×10^-2^ | -1.59×10^-3^ | - | n.s. | n.s. | n.s. | - |
|  |  |  | HI | 5.36×10^-1^ | n.s. | n.s. | n.s. | - | n.s. | n.s. | n.s. | - |
|  |  |  | PP | 4.63×10^-1^ | n.s. | 3.94×10^-2^ | -1.78×10^-3^ | - | n.s. | n.s. | n.s. | - |
|  |  |  | PB | 4.39×10^-1^ | n.s. | n.s. | n.s. | - | n.s. | n.s. | n.s. | - |
|  | R | Quadratic | ES | 5.50×10^-1^ | n.s. | 2.65×10^-2^ | -1.10×10^-3^ | - | n.s. | n.s. | n.s. | - |
|  |  |  | CP | 6.18×10^-1^ | n.s. | n.s. | n.s. | - | n.s. | n.s. | n.s. | - |
|  |  |  | HI | 6.21×10^-1^ | n.s. | n.s. | n.s. | - | n.s. | n.s. | n.s. | - |
|  |  |  | PP | 5.74×10^-1^ | n.s. | n.s. | n.s. | - | n.s. | n.s. | n.s. | - |
|  |  |  | PB | 4.75×10^-1^ | n.s. | n.s. | n.s. | - | n.s. | n.s. | n.s. | - |
| Lobule Ⅹ | L | None | ES | 1.47×10^-1^ | n.s. | n.s. | - | - | n.s. | - | - | - |
|  |  |  | CP | 1.47×10^-1^ | n.s. | n.s. | - | - | n.s. | - | - | - |
|  |  |  | HI | 1.45×10^-1^ | n.s. | n.s. | - | - | n.s. | - | - | - |
|  |  |  | PP | 1.46×10^-1^ | n.s. | n.s. | - | - | n.s. | - | - | - |
|  |  |  | PB | 1.45×10^-1^ | n.s. | n.s. | - | - | n.s. | - | - | - |
|  | R | None | ES | 1.75×10^-1^ | n.s. | n.s. | - | - | n.s. | - | - | - |
|  |  |  | CP | 1.76×10^-1^ | n.s. | n.s. | - | - | n.s. | - | - | - |
|  |  |  | HI | 1.73×10^-1^ | n.s. | n.s. | - | - | n.s. | - | - | - |
|  |  |  | PP | 1.76×10^-1^ | n.s. | n.s. | - | - | n.s. | - | - | - |
|  |  |  | PB | 1.75×10^-1^ | n.s. | n.s. | - | - | n.s. | - | - | - |

The level of significance is 0.0056 after multiple comparisons correction, – = not applicable; n.s. = non-significant, HS = Hemisphere, L = left, R = right, EBP = emotional and behavioural problems, ES = Emotional Symptoms, CP =Conduct Problems , HI = Hyperactivity/Inattention, PP = Peer Relationship Problems, PB = Prosocial Behaviors
